# General trends of chromosomal evolution in Aphidococca (Insecta, Homoptera, Aphidinea + Coccinea)

**DOI:** 10.3897/CompCytogen.v9i3.4930

**Published:** 2015-07-09

**Authors:** Ilya A. Gavrilov-Zimin, Andrey V. Stekolshchikov, D.C. Gautam

**Affiliations:** 1 Zoological Institute, Russian Academy of Sciences, Universitetskaya nab. 1, St. Petersburg 199034, Russia; 2 Department of Bio-Sciences, Himachal Pradesh University, Shimla, India

**Keywords:** Aphids, scale insects, chromosome numbers, genetic systems, evolution, phylogeny

## Abstract

Parallel trends of chromosomal evolution in Aphidococca are discussed, based on the catalogue of chromosomal numbers and genetic systems of scale insects by Gavrilov (2007) and the new catalogue for aphids provided in the present paper. To date chromosome numbers have been reported for 482 species of scale insects and for 1039 species of aphids, thus respectively comprising about 6% and 24% of the total number of species. Such characters as low modal numbers of chromosomes, heterochromatinization of part of chromosomes, production of only two sperm instead of four from each primary spermatocyte, physiological sex determination, "larval" meiosis, wide distribution of parthenogenesis and chromosomal races are considered as a result of homologous parallel changes of the initial genotype of Aphidococca ancestors. From a cytogenetic point of view, these characters separate Aphidococca from all other groups of Paraneoptera insects and in this sense can be considered as additional taxonomic characters. In contrast to available paleontological data the authors doubt that Coccinea with their very diverse (and partly primitive) genetic systems may have originated later then Aphidinea with their very specialised and unified genetic system.

## Introduction

**Figure 1.**
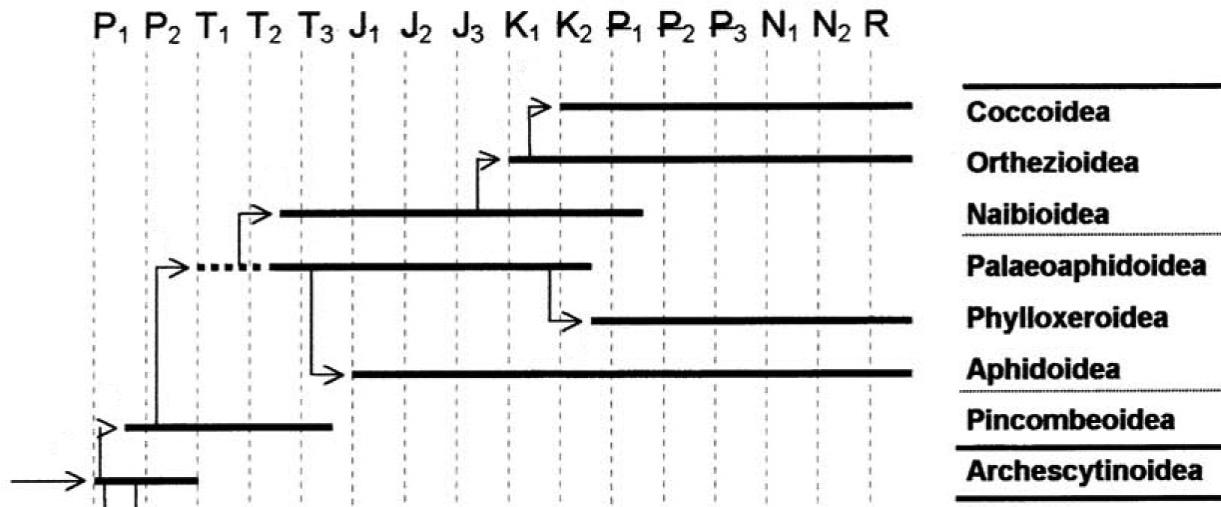
Phylogeny of Aphidococca placed on geochronological scale (after Shcherbakov and Popov 2002). Time periods: **P_1_, P_2_** Early (Lower) and Late (Upper) Permian **T_1_, T_2_, T_3_** Early, Middle and Late Triassic **J_1_, J_2_, J_3_** Early, Middle and Late Jurassic **K_1_, K_2_** Early and Late Cretaceous ₽**_1_** Palaeocene ₽**_2_** Eocene ₽**_3_** Oligocene **N_1_** Miocene **N_2_** Pliocene **R** present time (Holocene).

The name Aphidococca was recently introduced by Kluge (2010) for the taxon combining two closely related groups of Homoptera insects, aphids and scale insects. According to the paleontological data (see for example, Shcherbakov and Popov 2002, Shcherbakov 2007) scale insects (Coccinea) could originate from ancient aphids (Aphidinea) or aphid-like ancestors in the Triassic (Fig. 1). The close relationship of both groups is well supported by numerous morphological, anatomical, embryological, cytogenetic, physiological and other characters and, as it seems, is not disputed by any modern taxonomists. In the framework of cladistic taxonomy, aphids and scale insects are considered as sister groups (see for example, Wojciechowski 1992, Gullan and Cook 2007 and others) originating from a common ancestor. However, various theoretical generalizations and attempts at analysis of any biological characters of aphids and scale insects are usually done separately for these groups. Below we shall try to analyze aphids and scale insects as a united group which can be exactly contrasted to other related groups of Paraneoptera insects with particular regard to their cytogenetics.

At present, about 5000 species of aphids and 8000 species of scale insects have been recorded from all over the world (Favret and Eades in on-line "Aphid species file" database: http://aphid.speciesfile.org, Ben-Dov et al. in on-line "ScaleNet" database: http://www.sel.barc.usda.gov/scalenet/scalenet.htm). There is no general agreement on the higher classification within both groups; the number of accepted families and their relationships are disputed in the papers of different modern authors. In general, the opposite tendencies (splitting vs. integration) of the families take place in scale insect and aphid modern systematics. Thus, some modern coccidologists (for example, Hodgson 2014) accept till 33 extant families of scale insects in contrast to the 15-19 "large" traditionally accepted families (Danzig 1986, Danzig and Gavrilov-Zimin 2014), whereas the last taxonomic catalogue of aphids (Remaudière and Remaudière 1997) places all recent "true aphids" in the single family Aphididae, in contrast to the acceptance of 6-13 true aphid families by some other authors in addition to two families of "not true aphids", Adelgidae and Phylloxeridae (Börner 1952, Shaposhnikov 1964, Heie 1987, Heie and Wegierek 2009a, b). These opposite tendencies in the systematics of scale insects and aphids reflect, to our mind, the generally higher biological diversity of scale insects, which demonstrate more patterns of morphological, cytogenetic, physiological, and ecological specialization than aphids. Here, for further discussions we shall follow the system and nomenclature of Paraneoptera accepted recently in Gavrilov-Zimin and Danzig (2012) and Danzig and Gavrilov-Zimin (2014):

Phylogenetic line **Paraneoptera Martynov, 1923** (including 7 orders: Zoraptera, Copeognatha, Parasita, Thysanoptera, Homoptera, Coleorrhyncha, Heteroptera)

Cohort **Hemiptera Linnaeus, 1758** (= Condylognatha Börner, 1904, non Hemiptera auct.)

Superorder **Thysanoptera Haliday, 1836**

Superorder **Arthroidignatha Spinola, 1850** (= Hemiptera auct. non Linnaeus, 1758; = Rhynchota auct. non Burmeister, 1835)

Order **Coleorrhyncha Meyers & China, 1929**

Order **Heteroptera Latreille, 1810** (= Hemiptera auct. non Linnaeus, 1758)

Order **Homoptera auct. non Latreille, 1810**

Suborder **Cicadinea Batsch, 1789**

Suborder **Psyllinea Latreille, 1807**

Suborder **Aleyrodinea Newman, 1834**

Suborder **Aphidinea Latreille, 1802**

Superfamily **Adelgoidea Annand, 1928**

Superfamily **Phylloxeroidea Herrich-Schaeffer in Koch, 1854**

Superfamily **Aphidoidea Latreille, 1802**

Suborder **Coccinea Fallén, 1814** (= Coccoidea auct., Gallinsecta De Geer, 1776)

Superfamily **Orthezioidea Amyot & Serville, 1843** (=Paleococcoidea Borchsenius, 1950; = Archeococcidea Bodenheimer, 1952)

Superfamily **Coccoidea Fallén, 1814** (=Neococcoidea Borchsenius, 1950; = Neococcidea Bodenheimer, 1952)

Within the scale insects we recognize 19 extant familes (Table 1). Within the aphids we follow the system of Shaposhnikov (1964, 1985) with minor changes (taking into account some conclusions of Heie and Wegierek 2009a, b) (see Table 3), and accept 15 recent families.

**Table 1.** Variation of diploid chromosome number in 19 families of scale insects. **Kv** (index of karyotypic variability is provided only for the most studied families).

Family	Number of nominal taxa		Number of studied taxa		Range of variability	Kv	Modal chromosome numbers
	Genera	Species	Genera	Species			
Ortheziidae	22	202	3	3	14–18	-	-
Carayonemidae	4	4	-	-	-	-	-
Margarodidae s.l.	77	442	20	33	4–40	0.21	4, 6
Xenococcidae	3	33	-	-	-	-	-
Phenacoleachiidae	1	1	1	1	8	-	-
Pseudococcidae	279	2281	47	129	8–64	0.08	10
Eriococcidae	91	657	18	96	4–192	0.41	18
Kermesidae	10	90	1	2	26	-	-
Dactylopiidae	1	10	1	7	10–16	0.28	10
Asterolecaniidae	39	393	4	4	6–24	-	-
Stictococcidae	3	17	-	-	-	-	-
Micrococcidae	2	8	-	-	-	-	-
Aclerdidae	5	58	1	3	16–18	-	-
Coccidae	171	1133	27	50	10–36	0.22	16, 18
Kerriidae	9	102	2	4	18–20	-	-
Beesoniidae	6	16	-	-	-	-	-
Conchaspididae	4	30	1	1	12	-	-
Phoenicococcidae s.l. (including Halimococcidae)	6	22	5	7	10–18	-	10
Diaspididae	405	2479	68	141	6–18	0.04	8
**Total**	**1138**	**7978**	**199**	**482**	**4–192**		**8, 10, 18**

In the present paper we shall try to summarize data on chromosomal numbers, karyotypes and genetic systems of Aphidococca, mainly with regard to the evolutionary significance of these data, and try to demonstrate some previously neglected parallel tendences in the chromosomal evolution of aphids and scale insects. Two catalogues of chromosomal numbers and genetic systems are used as the basis for this discussion – the catalogue recently published by the first author (Gavrilov 2007) for scale insects, and a catalogue for aphids, compiled in the present paper from numerous scattered publications on aphid cytogenetics, the main sources being the tables in Kuznetsova and Shaposhnikov (1973), Blackman (1980, 1986) and the data from the monographs of Blackman and Eastop (1994, 2006) as well as from the on-line compilation of these monographs (Blackman and Eastop 2015: http://www.aphidsonworldsplants.info). We hope the combined catalogue will be useful for all collegues irrespective of any of our theoretical speculations.

## Chromosome numbers

To date chromosome numbers have been reported for 482 species of scale insects belonging to 14 of the 19 known families and for 1039 species of aphids belonging to 14 families (all of those accepted here for the recent aphid fauna) (Tables 1–4), thus respectively comprising about 6% and 24% of the total number of coccid and aphid species. Thus, the greater knowledge of aphid karyotype diversity in contrast to that of scale insects is obvious at the species level as well as for the higher taxa in both these groups.

**Table 2.** Additions to the Gavrilov’s (2007) catalogue of chromosome numbers and genetic systems of scale insects. (**H** – heterochromatinization of one haploid set of chromosomes without details of genetic system; **P(o)** – obligatory pathenogenesis).

Taxon	2n	Genetic system	Reference
Fam. Pseudococcidae			
*Balanococcus boratynskii* Williams, 1962	10	Lecanoid	Gavrilov and Trapeznikova 2010 [Belgorod Prov., Russia]
*Brevennia operta* (Borchsenius, 1949)	10	?	Gavrilov-Zimin 2011 [Turkey]
*Peliococcopsis priesneri* (Laing, 1936)	10	Lecanoid	Gavrilov-Zimin 2011 [Turkey]
*Phenacoccus hordei* (Lindeman, 1886)	10	Lecanoid	Gavrilov and Trapeznikova 2010 [Belgorod Prov., Russia]
*Phenacoccus specificus* Matesova, 1960	10	?	Gavrilov-Zimin 2011 [Turkey]
*Phenacoccus peruvianus* Granada de Willink, 2007	10	Lecanoid	Gavrilov and Trapeznikova 2010 [Portugal]
*Phenacoccus phenacoccoides* (Kiritshenko, 1932)	10+Bs	Lecanoid	Gavrilov-Zimin 2011 [Turkey]
Phenacoccus prope avenae Borchsenius, 1949	10	Lecanoid	Gavrilov and Trapeznikova 2010 [Portugal]
*Phenacoccus tergrigorianae* Borchsenius, 1956	10	Lecanoid	Gavrilov-Zimin 2011 [Turkey]
*Puto superbus* (Leonardi, 1907)	16/17	XX/X0	Gavrilov-Zimin 2011 [Turkey]
*Rhizoecus halophilus* (Hardy, 1868)	10	Lecanoid	Gavrilov and Trapeznikova 2010 [Bulgaria]
*Trabutina crassispinosa* Borchsenius, 1941	16	?	Gavrilov-Zimin 2011 [Turkey]
*Trionymus artemisiarum* (Borchsenius, 1949)	10	Lecanoid	Gavrilov-Zimin 2011 [Turkey]
*Trionymus haancheni* MkKenzie, 1960	16	Lecanoid	Gavrilov and Trapeznikova 2007 [USA]
*Trionymus radicum* (Newstead, 1895)	10	Lecanoid	Gavrilov and Trapeznikova 2010 [Bulgaria]
**Fam. Eriococcidae**			
*Acanthococcus lactucae* Borchsenius, 1949	16	?Comstockioid	Gavrilov-Zimin 2011 [Turkey]
**Fam. Kermesidae**			
*Kermes roboris* (Fourcroy, 1785)	26	?Comstockioid	Gavrilov-Zimin 2011 [Turkey]
**Fam. Aclerdidae**			
*Aclerda pseudozoysiae* Gavrilov-Zimin, 2012	16	H	Gavrilov-Zimin 2012 [New Guinea, Indonesia]
*Aclerda takahashii* Kuwana, 1932	18	P(o)	Gavrilov-Zimin 2012 [Sulawesi, Indonesiai]
**Fam. Coccidae**			
*Phyllostroma myrtilli* (Kaltenbach, 1874)	16	P, deuterotoky	Gavrilov and Trapeznikova 2008 [Bulgaria]
*Lecanopsis turcica* (Bodenheimer, 1951)	18	H	Gavrilov-Zimin 2011 [Turkey]
*Acanthopulvinaria orientalis* (Nasonov, 1908)	18 16	H H	Gavrilov 2007 [Astrakhan, Russia] Gavrilov-Zimin 2011 [Turkey]
*Anapulvinaria pistaciae* (Bodenheimer, 1926)	16?	H	Gavrilov-Zimin 2011 [Turkey]

The smallest chromosome number is the same for aphids and for scale insects, 2n=4, and known in species of the tribe Iceryini (Coccinea: Margarodidae), in the genus *Apiomorpha* Rübsaamen, 1894 (Coccinea: Eriococcidae) (Hughes-Schrader 1925, 1930, 1948, 1963) and in the genus *Amphorophora* Buckton, 1876 (Aphidinea: Aphididae) (Blackman 1985). The greatest numbers are 2n=72 (in *Amphorophora sensoriata* Mason, 1923) (Blackman 1980) and 2n≈192 (in *Apiomorpha macqueeni* Froggatt, 1929 (Cook 2000). It is interesting that, both in aphids and in scale insects, the entire range of variation of chromosome number for the suborders is found in one genus in each group – *Amphorophora* in Aphidinea and *Apiomorpha* in Coccinea.

The range of diploid number variability, 2n=4-192, demonstrated by Aphidococca is wider than in any other group of Paraneoptera, including even such huge groups as Cicadinea and Heteroptera. Thus, for the groups of Homoptera nearest to Aphidococca the following diploid chromosome numbers have been reported: Aleyrodinea, 2n=20-26 (Blackman and Cahill 1998, but only a few species were studied until now); Psyllinea, 2n=8-26 (Maryańska-Nadachowska 2002); Cicadinea, 2n=8-38 (Emeljanov and Kirillova 1989, 1991). For Heteroptera the range of variability reported is 2n=6-80 (Ueshima 1979, Kuznetsova et al. 2011), for Thysanoptera 2n=20-106 (Brio et al. 2010), for Parasita (Mallophaga + Anoplura) 2n=4-24 (Golub and Nokkala 2004), and for the most ancient and primitive Paraneoptera group, Copeognatha – 2n=14-30 (Golub and Nokkala 2009).

Modal chromosomal numbers of Aphidococca as a whole, 2n=8, 10, 12, 18 are lower (with a small overlap) than in other Homoptera, and most other Paraneoptera groups that have been sufficiently studied to provide reliable data. Thus, comparable modal numbers are 2n=26 for Psyllinea (Maryańska-Nadachowska 2002), 2n=18, 20, 22, 26, 30 for Cicadinea (Emeljanov and Kirillova 1989, 1991), 2n= 14, 22, 26, 28, 34 for Heteroptera (Ueshima 1979, Kuznetsova et al. 2011), and 2n=18 for Copeognatha (Golub and Nokkala 2004). Aleyrodinea and Thysanoptera are too poorly studied for reliable comparison, but for both these groups there are no recorded chromosome numbers lower than 2n=20. What can be a reason for the comparatively low modal numbers of Aphidococca? It is well known that there is no direct correlation between chromosomal number and complexity of an organism. On the other hand, if we look for the most general character that Aphidococca share with another group with low chromosomal numbers, the Parasita, but not with other Paraneoptera groups, we shall see that the tendency for lower modal numbers within the Paraneoptera correlates with a tendency to larvalization of imaginal structures or neoteny, with reduction of the number of postembryonic stages to three—five in Aphidococca and Parasita, in comparison with the six developmental stages usually found in most Paraneoptera.

The karyotype diversity within Aphidococca families can be characterized by a simple index of karyotypic variability (Kv) which is equal to the quantity of different diploid chromosome numbers in the taxon, divided by the number of cytogenetically studied species in this taxon. For example, in the family Diaspididae (Coccinea) six variants (2n= 6, 8, 10, 12, 16, 18) of the chromosomal number are known for 141 studied species. So, for Diaspididae, Kv is equal 6/141=0.04. Of course, Kv, based on the present available data may be changed when more species are studied, but it seems this change will not be very significant. Thus, if we calculate Kv for aphid families based on the old catalogue of Kuznetsova and Shaposhnikov (1973), we obtain values similar to those based on the present catalogue (Table 3), although the number of species studied has meanwhile increased 3–4 times. It is easy to see that Kv is smallest in the largest families of Aphidococca which include numerous poorly identified (recently diverged?) species: Aphididae (0.03), Diaspididae (0.04), Pseudococcidae (0.08). On the contrary, ancient families with a limited number of recent species show comparatively large Kv-s: Adelgidae (0.22), Phylloxeridae (0.45), Margarodidae s.l. (0.21). High Kv-s in some other families, for example, Eriococcidae (0.45) or Thelaxidae (0.66), are connected mainly with enormous variability of chromosomal number not in the family as a whole, but in one of the genera (*Apiomorpha* and *Glyphina* Koch, 1856 respectively).

**Table 3.** Variation of diploid chromosome number in 14 families of aphids.

Family	Number of nominal taxa		Number of studied taxa		Range of variability	Kv	Modal chromo-some numbers
	Genera	Species	Genera	Species			
Adelgidae	7	69	7	18	16–24	0.22	-
Phylloxeridae	8	73	4	10	6–12	0.45	-
Eriosomatidae	53	369	28	85	6–38	0.16	10, 12, 20
Mindaridae	1	9	1	2	8–12	0.30	-
Lachnidae	18	401	11	73	6-c.60	0.20	10, 12, 14
Hormaphididae	44	221	9	26	8- c.50	-	12
Thelaxidae	4	18	3	8	8–56	0.66	-
Tamaliidae	1	6	-	-	-	-	-
Aiceonidae	1	18	1	1	18	-	-
Anoeciidae	2	30	1	7	6–12	0.50	-
Phloeomyzidae	1	1	1	1	10	-	-
Greenideidae	16	178	6	21	7–40	0.36	-
Drepanosiphidae	92	573	48	141	6-c. 48	0.09	8, 14, 18
Chaitophoridae	13	178	4	39	(4?) 6–40	-	-
Aphididae	273	3033	120	605	4–72	0.03	8, 10, 12
**Total**	**534**	**5177**	**243**	**1039**	**4–72**		

In the higher (above family level) taxonomic groups of Paraneoptera the utility of Kv index is currently limited by the low percentage of studied species and by limited variation of chromosomal number itself, because there are thousands of species in these higher taxa, whereas chromosomal numbers higher than 2n=60 are very rare and higher than 2n=192 are unknown.

## Intrageneric and intraspecific chromosomal variability

A typical Aphidococca karyotype has rod-like chromosomes whose number is more or less stable within a genus (with some notable exeptions which will be discussed below). For example, in the species-rich genus *Aphis* Linnaeus, 1758, the diploid chromosome number in majority of studied species is eight (2n=8) with only a few exceptions. Moreover, most of the species in the young and large tribe Aphidini of the family Aphididae have 2n=8, and the same situation applies to the youngest and largest family of scale insects, Diaspididae, the overwhelming majority of species of which also have 2n=8. On the other hand, many genera of Aphidococca demonstrate significant or even extraordinary variation of chromosome number, and, moreover, several diploid numbers can be found in the same nominal species. The most impressive example of such variation is in the scale insect genus *Apiomorpha* with its 42 diploid numbers, ranging from 2n=4 to 2n≈192 in 47 studied species (Hughes-Schrader 1925, 1930, 1948, 1963, Cook 2000, 2001). A number of aphid genera, for example, *Phylloxera* Boyer de Fonscolombe, 1834, *Glyphina* Koch, 1856, *Forda* von Heyden, 1837, *Tetraneura* Hartig, 1841, *Cinara* Curtis, 1835, *Lachnus* Burmeister, 1835, *Trama* von Heyden, 1837, *Amphorophora* Buckton, 1876, *Euceraphis* Walker, 1870, *Chaitophorus* Koch, 1854 and others also demonstrate a great variability in diploid number both between and within nominal species (see Table 4).

**Figure 2.**
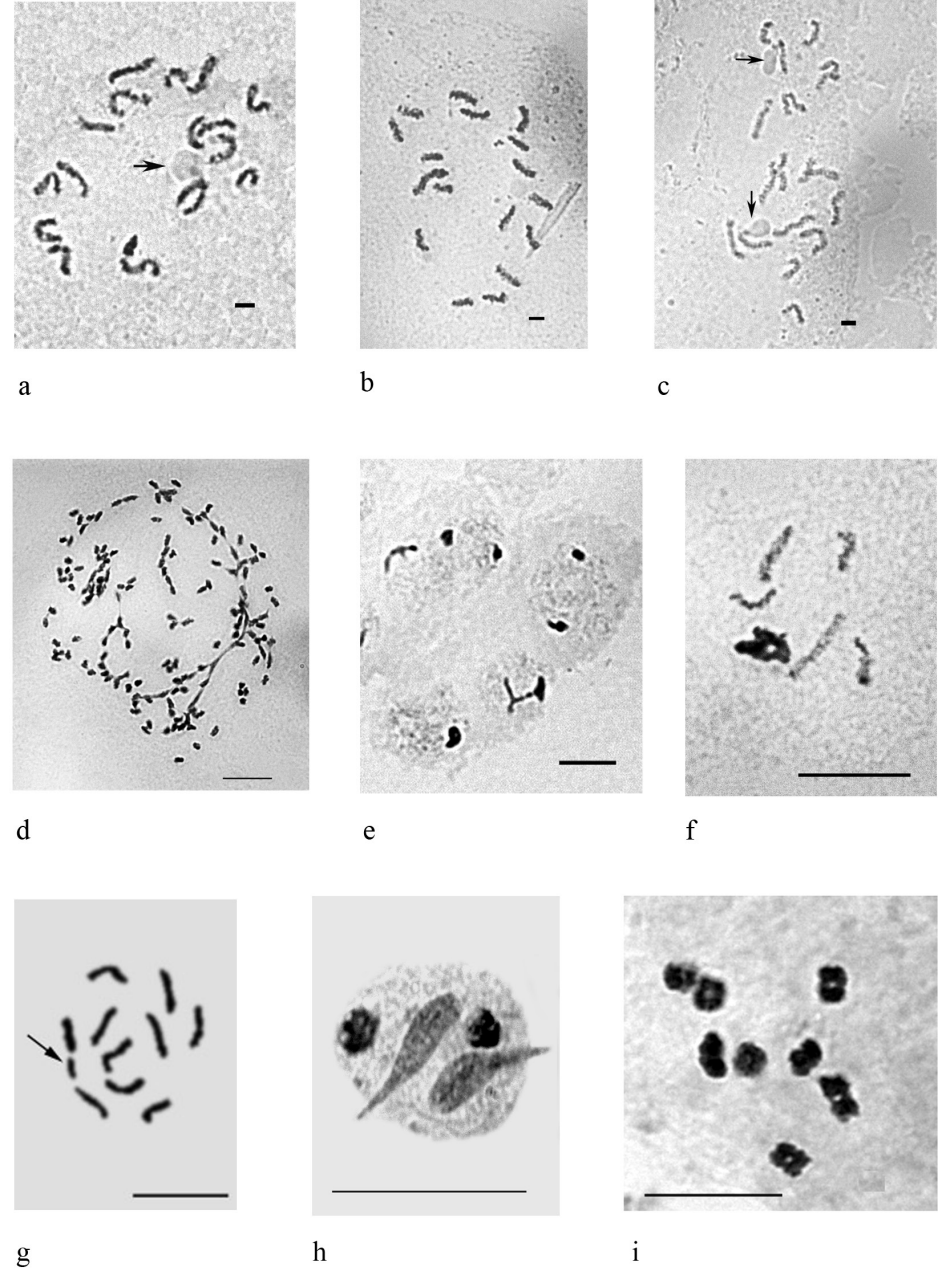
Mitotic and meiotic chromosomes of different scale insects. **a–c** *Puto superbus* (Leonardi, 1907), **a** cell of female embryo, 2n=18 **b–c** cells of male embryo, 2n=17, chromosomes with NORs are arrowed **d** *Heliococcus sulci* Goux, 1934, polyploid cell, about 140 chromosomes with numerous agglutinations **e** *Peliococcopsis priesneri* (Laing, 1936), male embryonic cells at interphase stage with one haploid set heterochomatinized **f** *Planococcus vovae* (Nasonov, 1908), male embryonic cell with one haploid set heterochomatinized **g** *Dysmicoccus multivorus* (Kiritshenko, 1936), embryonic cell with 2n=10 + B, additional chromosomal element arrowed **h** *Chloropulvinaria aurantii* (Cockerell, 1896), 2n=26, spermatid **i** *Protopulvinaria pyriformis* (Cockerell, 1894), 2n=16, oogonial metaphase I. Bar = 10 µm.

Polyploidy is a very rare phenomenon in Aphidococca as in other Paraneoptera and probably does not play a significant role in the evolution of the group. For scale insects a polyploid (triploid) karyotype was reported for *Physokermes hemicryphus* (Dalman, 1826) from the family Coccidae (Nur 1979), but theoretically may be found to occur in some other species of soft scales, felt scales or mealybugs which have chromosome numbers three or four times those of species known to be diploid in the same genera. In aphids polyploid species are not known at all, but several cases of polyploidization in parthenogenetic populations have been reported (see discussion in Blackman 1987). On the other hand, females usually have highly polyploid cells (Fig. 2d) in bacteriomes, peculiar organs which include intracellular symbiotic bacteria.

Accessory chromosomal elements have been found in several species of mealybugs (Pseudococcidae) (Nur et al. 1987, Gavrilov 2007) (Fig. 2g), in one species of the Margarodidae (Hughes-Schrader 1942), in two species of soft scales (Coccidae) (Gavrilov 2007) and in some armored scales (Diaspididae) (Brown 1960). Blackman (1980, 1990) noted presumed B-chromosomes in numerous aphid species from different families, especially in anholocyclic populations, and these B-chromosomes are probably relicts of multiple X-chromosomes.

## Evolution of genetic systems

**Figure 3.**
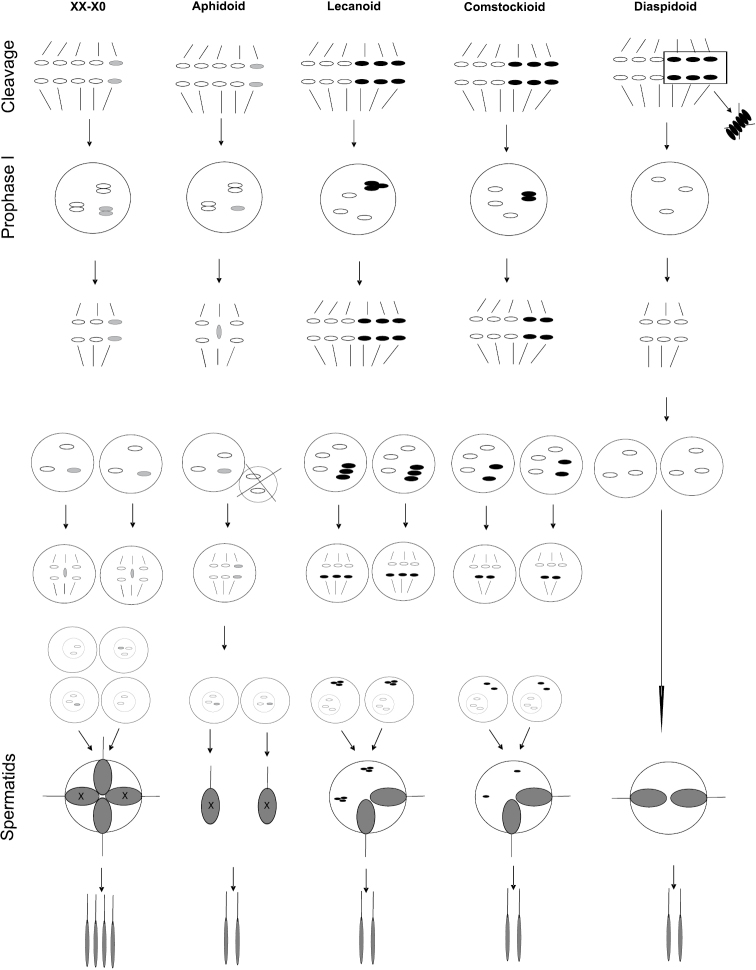
Meiosis and spermatogenesis encountered in different genetic systems of Aphidococca.

In contrast to other Paraneoptera, all Aphidococca have spermatocyte and oocyte meiosis in larvae or in neotenic females (which are in fact equivalent to larvae as in scale insects) and demonstrate a multiplicity of very different and unique genetic systems, which are probably based on an original XX-X0 system, considered by Blackman (1995) as ancestral for all Paraneoptera insects (Fig. 3). In species possessing this system, the sex of the progeny is determined during spermatogenesis. Spermatozoa with X-chromosomes produce females and spermatozoa without X-chromosomes produce males. This usual type of XX-X0 spermatogenesis (similar to that of Copeognatha, for example) is known in some primitive scale insects (some Margarodidae, Ortheziidae, genus *Puto* Signoret, 1875 (Pseudococcidae)) (Hughes-Schrader 1931, 1942, 1944, 1955, Brown and Cleveland 1968) with only one peculiar character – spermatocytes fuse to form a quadrinucleate spermatid (Fig. 3). This fusion can be considered as a unique apomorphy of Coccinea. In some genera of Margarodidae, such as *Aspidoproctus* Newstead, 1901, *Protortonia* Townsend, 1898, *Llaveia* Signoret, 1876, *Llaveiela* Morrison, 1927, *Nautococus* Vayssière, 1939 (all from the subfamily Monophlebinae) XX-X0 spermatogenesis is also complicated by the enclosure of meotic prophase I chromosomes in peculiar separate vesicles, instead of a single nuclear membrane. This phenomenon was discovered by F. Schrader and S. Hughes-Schrader and was comprehensively reviewed by Hughes-Schrader (1948). Moreover, it is interesting to note that in *Protortonia* (Coccinea: Margarodidae), in the second meiotic division, all chromosomes form a chain stretched between the two poles of the cell (Schrader 1931), which is similar to the well-known example of chain formation in plants of the genus *Oenothera* Linnaeus, 1753 (Onagraceae) and some other plants and animals (White 1973).

In most cases, species with the XX-X0 system have ony one pair of sex chromosomes in their karyotypes. For example, females of *Porphyrophora polonica* (Linnaeus, 1758) (Coccinea: Margarodidae) have 2n=12+XX and males have 2n=12+X. However examples of multiple sex chromosomes are also known. Thus, species of the family Adelgidae (Aphidinea) have up to four pairs of X chromosomes, and some species of the families Phylloxeridae, Eriosomatidae, Lachnidae and Drepanosiphidae (Aphidinea) have one-two pairs of sex chromosomes (see Table 4). In scale insects, only *Matsucoccus gallicolus* Morrison, 1939 (Margarodidae) has a multiple sex chromosome system with 6 pairs of X chromosomes (2n=28+12X in females and 2n=28+6X in males), which probably evolved as a result of fragmentation of an initial pair of X chromosomes (Hughes-Schrader 1948) and it seems the number of sex chromosomes in this species is the highest known in Insecta. Multiple sex chromosomes are also known in Cicadinea and Heteroptera and can be probably considered as a non-unique apomorpic character in different genera of proboscidian insects (Arthroidignatha). This character is not known in studied Copeognatha (Golub and Nokkala 2009), which is considered as an ancestor group for proboscidians.

Hales (1989) reported a peculiar fusion of multiple X chromosomes with autosomes (X_1_+A, X_1_, X_2_+A, X_2_) in somatic cells of *Schoutedenia lutea* (van der Goot, 1917) (Aphidinea, Greenideidae), that demonstrates a special genetic system unknown in other aphids and in Paraneoptera as a whole, but this phenomenon needs further investigation.

Hovewer, in the majority of studied scale insects and in all studied aphids sex determination is not brought about by stochastic combination of male and female chromosome sets during fertilization, because male and female gametes in most Aphidococca are cytogenetically identical and physiological sex determination takes place. Thus, in all studied Aphidinea, gametogenesis is of a unified type and based on an XX-X0 mechanism, but has unique features which are probably unknown in any other animals with XX-X0. One of the secondary spermatocytes (which includes autosomes only) is smaller in size and degenerates just after anaphase I. The second, larger spermatocyte gives origin to two sperms; both with one X-chromosome (see Manicardi et al. 2015 and our Fig. 2). Thus, aphid males give rise only to female-producing sperm, and sexual females also produce only female-producing oocytes, so that all sexually-produced progeny are female. On the other hand, parthenogenetic females can produce embryos which are either XX or X0, using a special cytological mechanism in which the X-chromosome is lost in some of the oocytes (Orlando 1974, Blackman and Hales 1986). Thus, sex of progeny is totally dependent on the physiology of the parthenogenetic female, which starts to produce sexuales under certain environmental conditions. This mode of gametogenesis is closely connected with cyclic parthenogenesis and is undoubtedly a unique apomorphy of Aphidinea. In general we suggest that the genetic system of aphids could be termed the Aphidoid system for the uniformity with the names of the genetic systems of scale insects (see below).

The majority of scale insects (almost whole superfamily Coccoidea) and aphids of the tribe Tramini (Lachnidae) demonstrate specific heterochromatinization of part of chromosomes in their diploid set. The species of scale insects with Lecanoid, Comstockioid, and Diaspidoid genetic systems feature obligate heterochromatinization of the paternal set of chromosomes in the males (Fig. 2e–f). Paternal genome heterochromatinization (PGH) is known in some groups of insects (see reviews of White 1973 and Normark 2003), but in each of these groups PGH has specific characters and forms unique genetic systems. The coccid species with systems Lecanoid, Comstockioid, and Diaspidoid can be purely sexual with identical male and female gametes, or demonstrate diploid arrhenotoky and deuterotoky in addition to heterochromatinization of the paternal set of chromosomes. In all these cases the sex of the progeny depends on rather enigmatic physiological processes occurring inside the female, as in the Aphidoid system.

In the Lecanoid system, the heterochromatic chromosome set exists during all stages of the male life cycle. During meiosis in the male, the chromosomes do not pair and separate equationally during the first division. During the second division, two metaphase plates are formed, and the heterochromatic and euchromatic chromosomes then segregate to the opposite poles (Hughes-Schrader 1948, Nur 1980). As a result of meiosis, quadrinucleate spermatids are formed, but only the nuclei of maternal origin produce sperm (Fig. 3).

In the Comstockioid system, the heterochromatic set is partly (as separate chromosomes) eliminated during embryogenesis and different cells of the same tissue may differ in chromosome number. According to the number of eliminated chromosomes, several variants of the Comstockiella system are known: CL^I^ – Comstockioid-Lecanoid intermediates, C^varH^ – Comstockioid with one pair of paternal chromosomes, retained in different cysts, C^C^ – complete Comstockioid. The course of spermatogenesis varies among the different taxa, depending on the number of non-eliminated heterochromatic chromosomes. If all these chromosomes are destroyed, the second division is absent (Brown 1965, 1967, Nur 1980).

In the Diaspidoid system, the heterochromatic set has been completely lost, and adult males are haploid. Hence, spermatogenesis consists of a single equational division (Brown 1965, 1967, Nur 1980, 1982).

In the aphid tribe Tramini (Lachnidae), almost all studied populations reproduce by thelytokous parthenogenesis and sex chromosomes have not been identified (Blackman 1980, 1990, Blackman et al. 2000). Some of the chromosomes in the diploid set demonstrate heterochromatinization and even aggregation of heterochromatic elements in somatic cells until late prophase (Blackman 1980), thus resembling the Lecanoid-Comstockioid genetic system in scale insects. However, heterochomatic chromosomes in Tramini can vary significantly in number between populations and do not constitute a haploid set. These heterochromatic elements of Tramini are similar to B-chromosomes, and Blackman et al. (2000) suggest that they may be derived from ancestral redundant X chromosomes.

In the tribe Fordini (Aphidinea: Eriosomatidae), germ-line and somatic cells have radically different chromosome numbers (Blackman 1980). Unfortunately this very interesting phenomenon has not been additionally studied.

*Hermaphroditism* and *Haplo-diploidy* are known only in species of the tribe Iceryini (Coccinea: Margarodidae) (Hughes-Schrader 1948, 1963). The hermaphrodites are diploid and similar to females in their morphology and mode of life. During embryogenesis the gonads of these insects do not undergo sexual differentiation. Later, in the crawlers, haploid nuclei appear in the gonads and form the central testicular part of a hermaphroditic gland. The haploid nuclei appear as a result of degeneration and elimination of one set of chromosomes. The peripheral ovarian part of the gland is diploid and formed a little later. Fertilization takes place either in the ovarian part or in the cavity of the ovo-testis. Fertilized eggs always develop into female-like hermaphrodites, which usually reproduce by self-fertilization. However, the hermaphrodites may also copulate with accidental haploid males, which sometimes develop from unfertilized eggs (Hughes-Schrader 1948). *Haplo-diploidy* is known in Iceryini scale insects only and is in fact a result of haploid arrhenotoky as in other insects with haploid males. Fertilized eggs produce diploid females and unfertilized eggs produce haploid males (Hughes-Schrader 1948).

To date, species with heteromorphic sex chromosomes (genetic system XX/XY, neo-XX/XY) have not been found among Aphidococca in contrast to larger groups of Paraneoptera: Cicadinea + Heteroptera, where these systems are very common and to Psyllinea + Copeognatha, where XX/XY (or neo-XX/XY) system is known in several species. On the other hand, in some species of scale insects, such as *Newsteadia* sp., *Praelongorthezia praelonga* (Douglas, 1891) (both from Ortheziidae), *Lachnodius eucalypti* (Maskell, 1892) (Eriococcidae), and *Stictococcus* sp. (Stictococcidae), both females and males have the same number of chromosomes, but without distinct sex chromosomes or peculiar heterochromatinization of the paternal set (as in the unique coccid systems Lecanoid, Comstockioid, and Diaspidoid). Thus, the Australian felt scale *Lachnodius eucalypti*, having 2n=18 in both females and males (Brown, 1967, 1977, Nur, 1980), is especially noteworthy. In other studied species of the genus *Lachnodius* Maskell, 1896 and in the family Eriococcidae as a whole, the Comstockioid system has been discovered, but in males of *Lachnodius eucalypti* heterochromatinization of the paternal set is absent. The 2n-2n system probably evolved in scale insects more than once and from different ancestral systems: from the system with heterochromatinization in *Lachnodius eucalypti* and *Stictococcus* sp. and from the XX-X0 system in *Praelongorthezia praelonga* (Nur 1980). Meiosis in *Lachnodius eucalypti* comprises one reductional division only (Brown and Chandra 1977), whereas in *Praelongorthezia praelonga* it comprises two divisions without an inverted meiotic sequence (Brown 1958).

## Parthenogenesis

It seems that absolutely all aphid species and many scale insects can produce their progeny by parthenogenesis. In aphids the parthenogenesis can be cyclic (with alternation of thelytoky and deuterotoky – the apomorphic condition for Aphidinea) or anholocyclic (with continuous thelytoky). In scale insects no examples of cyclic parthenogenesis are known and parthenogenesis can be thelytokous, deuterotokous or arrhenotokous. On the other hand, there are probably a few obligatory thelytokous species of scale insects, such as *Protopulvinaria pyriformis* (Cockerell, 1894) and *Eupulvinaria peregrina* Borchsenius, 1953 (Gavrilov and Trapeznikova 2008), which never produce males in any population or geographical region. A great many species, often reported as thelytokous (see, for example, Nur 1990 for the review), in reality combine thelytokous reproduction with amphimixis, producing males amphimictically or parthenogenetically (diploid arrhenotoky and deuterotoky), and these males have, as usual for scale insects, paternal genome heterochromatinization. Some species variously manifest thelytokous and sexual lineages in different geographical regions or on different host plants (Nur 1990). Haploid arrenotoky (noted above for Icerini) is connected with haplo-diploidy and can be interpreted as facultative, rather than obligatory parthenogenesis.

Unfortunately it is impossible to say now exactly how many scale insects species are able to reproduce by parthenogenesis, and this ignorance hampers a detailed comparison of scale insects and aphids in this respect.

## Conclusion

Finally we can underline the following parallel trends in the evolution of Aphidinea and Coccinea:

1) Low modal numbers of chromosomes.

2) Heterochromatinization of part of chromosomes.

3) Production of only two sperms instead of four from each primary spermatocyte.

4) Physiological sex determination.

5) "Larval" meiosis.

6) Widely distributed parthenogenesis.

7) Intraspecific chromosomal races (some of which may be cryptic species).

We consider that at least some of these tendencies may be regarded as additional taxonomic characters, which support the erection of Aphidococca as a higher category differing radically from other Homoptera and more widely from all Paraneoptera groups.

A comparison of cytogenetic data between the two groups of Aphidococca shows that Coccinea demonstrate much more diverse cytogenetic characteristics than Aphidinea. From the cytogenetic point of view Coccinea seem to be more primitive, including specialized (in most families) as well as ancient plesiomorphic characters (in some families): a simple XX-X0 genetic system with production of 4 sperms from one primary spermatocyte, chromosomal (not physiological) sex determination, simple bisexual reproduction, and later initiation of meiosis, i.e. characters which have been lost completely in all studied aphids. This deduction contradicts the current interpretation of paleontological data (discussed in the Introduction). It is difficult to imagine that the diverse (and partly primitive) cytogenetic mechanisms of scale insects could have originated from the very specialised and unified Aphidoid genetic system. We therefore suppose that the ancient scale insects originated at least not later than ancient aphids. The contradiction with the paleontological record may be explained by the well-known incompleteness of this record and the very limited number of taxonomic characters for fossil groups (mainly wing venation in ancient Aphidococca), which results in a very subjective identification of fossil insects. Thus, for example, fossil Naibiidae were described by Shcherbakov (1990, 2007) as most ancient, four-winged scale insects, but the same group is considered to be aphids by some aphidologists (see, for example, Wojciechowski 1992). The Lower Jurassic *Mesococcus asiaticus* Becker-Megdisova, 1960, which demonstrates the unique facies of a neotenic scale insect female, was considered by the original author as an ancient scale insect, similar to modern Monophlebinae, but was excluded from scale insects (and not placed in any other group!) by Koteja 1990.

**Table 4.** Chromosome numbers and genetic systems of Aphidinea. **P(c)** – cyclic parthenogenesis, **P(o)** – obligatory parthenogenesis in anholocyclic species, **B** – additional chromosomes.

Taxon	Life cycle	2n ♀/♂	Genetic system, ♀/♂	References and collecting data
**Superfam. PHYLLOXEROIDEA** **Fam. Adelgidae**				
*Adelges geniculatus* (Ratzeburg, 1844)	P(o)	20		Steffan 1968b [Germany; Canada]
*Adelges laricis* Vallot, 1836	P(c)	22, 21, 20/18	2(X_1_X_2_X_3_X_4_)/ X_1_X_2_X_3_X_4_0	Frolowa 1924 (as *Chermes strobilobius*) [Moscow, Russia]
		20/18	2(X_1_X_2_)/X_1_X_2_0	Steffan 1968b [Germany]
*Adelges tardus* (Dreyfus, 1888)	P(o)	20		Steffan 1968b [Germany]
*Aphrastasia pectinatae* (Cholodkovsky, 1888)	P(c)	20/18	2(X_1_X_2_)/ X_1_X_2_0	Frolowa 1924 (as *Chermes*) [Moscow, Russia]
*Cholodkovskya viridana* (Cholodkovsky, 1888)	P(o)	24		Steffan 1968b [Germany]
*Dreyfusia nordmannianae* (Eckstein, 1890)	P(c)	22		Steffan 1968b [Germany]
*Gillettella cooleyi* (Gillette, 1907)	P(c)	22/20	2(X_1_X_2_)/ X_1_X_2_0	Steffan 1968a, b [Germany; Canada]
*Gillettella coweni* (Gillette, 1907)	P(o)	22		Steffan 1968a, b [Canada]
*Pineus boerneri* Annand, 1928	P(o)	16		Blackman and Eastop 1994 [Hawaii, USA]
		17		Blackman and Eastop 1994 [California, USA; Africa; Australia; New Zealand]
*Pineus cembrae* (Cholodkovsky, 1888)	P(c)	18		Blackman and Eastop 1994 [?]
*Pineus orientalis* (Dreyfus, 1889)	P(c)	20		Blackman and Eastop 1994 [?]
*Pineus pineoides* (Cholodkovsky, 1903)	P(o)	17		Blackman and Eastop 1994 [?]
		22		Steffan 1968b [Germany; Canada]
*Pineus pini* (Goeze, 1778)	P(o)	19		Blackman and Eastop 1994 [New Zeland]
		20		Blackman and Eastop 1994, Blackman et al. 1995 [Europe]
		21		Blackman and Eastop 1994, Blackman et al. 1995 [Australia]
		22		Steffan 1968b [Germany]
*Pineus similis* (Gillette, 1907)	P(o)	22		Steffan 1968b [Canada]
*Pineus strobi* (Hartig, 1839)	P(o)	20		Blackman and Eastop 1994 [?]
		22		Steffan 1968b [Germany; Canada]
Pineus (Pineoides) pinifoliae (Fitch, 1858)	P(c)	22		Steffan 1968b [Canada]
*Sacchiphantes abietis* (Linnaeus, 1758)	P(o)	18		Pagliai 1967 [Italy], Steffan 1968b [Germany]
		20		Steffan 1968b (as *Sacchiphantes laricifoliae* (Fitch, 1858)) [Canada; USA]
*Sacchiphantes viridis* (Ratzeburg, 1843)	P(c)	18/16	2(X_1_X_2_)/ X_1_X_2_0	Steffan 1968a, b [Germany]
**Fam. Phylloxeridae**				
*Aphanostigma piri* (Cholodkovsky, 1903)	P(c)	8		Wysoki and Swirsky 1970 [Israel]
*Daktulosphaira vitifoliae* (Fitch, 1851)	P(c), P(o)	10/9	XX/X0	Maillet 1957 [France]; Forneck et al. 1999 [Europe; USA]
*Moritziella caryaefoliae* (Fitch, 1856)	P(o)	8		Morgan 1909b (as *Phylloxera*) [USA]
*Phylloxera caryaecaulis* (Fitch, 1855)	P(c)	8/6	2(X_1_X_2_)/ X_1_X_2_0	Morgan 1909a, 1912, 1915 [USA]
*Phylloxera caryaefallax* Riley, 1875	?	12		Morgan 1909a, 1912, 1915 (as *Phylloxera fallax*) [USA]
*Phylloxera caryaeglobuli* Walsh, 1863	?	22		Morgan 1906, 1909b [USA]
*Phylloxera depressa* (Shimer, 1869)	?	6		Morgan 1909b [USA]
*Phylloxera globosa* (Shimer, 1867)	?	6		Morgan 1906, 1909b [USA]
*Phylloxera quercus* Boyer de Fonscolombe, 1834	P(c), P(o)	12/11	XX/X0	Maillet 1957 [France]
*Phylloxera subelliptica* (Shimer, 1869)	?	6		Morgan 1909b [USA]
*Phylloxera* sp.	?	12		Morgan 1906 [USA]
**Superfam. APHIDOIDEA** **Fam. Eriosomatidae**				
*Aloephagus myersi* Essig, 1950	P(c), P(o)	22		Blackman and Eastop 1984 [?]
*Aploneura lentisci* (Passerini, 1856)	P(c), P(o)	16		Blackman 1980 (as *Asiphum*) [Great Britain], Blackman and Spence 1996 [Great Britain]
*Appendiseta robiniae* (Gillette, 1907)	P(c)	10		Blackman and Eastop 1994 [?]
*Baizongia pistaciae* (Linnaeus, 1767)	P(c), P(o)	24		Blackman 1980 [Great Britain] (anholocyclic population)
*Colopha compressa* (Koch, 1856)	P(c), P(o)	16		Blackman 1980 [Great Britain]
*Colopha kansugei* (Uye, 1924)	P(o), ? P(c)	10		Blackman 1986 [Japan]
*Colophina arma* Aoki, 1977	P(c)	10 (female), 8 (male)	2(X_1_X_2_)/X_1_X_2_0	Blackman 1986 [Japan]
*Colophina clematicola* (Shinji, 1922)	?P(c), P(o)	20		Blackman and Eastop 2015 [?]
*Colophina clematis* (Shinji, 1922)	P(c), P(o)	10 +B (female), 8+B (male)	2(X_1_X_2_)/X_1_X_2_0	Blackman 1986 [Japan]
*Epipemphigus imaicus* (Cholodkovsky, 1912)	P(c)	18		Khuda-Bukhsh 1980, Khuda-Bukhsh and Pal 1983a [Garhwal, Uttarakhand, India]
*Epipemphigus niisimae* (Matsumura, 1917)	P(c)	20		Blackman 1986 [Japan]
*Eriosoma crataegi* (Oestlund, 1887)	P(c)	12		Robinson and Chen 1969a [Canada]
*Eriosoma lanigerum* (Hausmann, 1802)	P(c), P (o)	12		Baehr 1908, 1909 (as *Schizoneura*) [Germany], Pagliai 1963 [Italy], Sun and Robinson 1966, Harper and MacDonald 1966, Robinson and Chen 1969a [Canada], Kulkarni and Kacker 1980 [India], Gautam and Verma 1982, Kulkarni 1984 [Shimla, Himachal Pradesh, India]
		12/11	XX/X0	Gautam and Verma 1983 [Shimla, Himachal Pradesh, India]
Eriosoma (Mimaphidus) lanuginosum (Hartig, 1839)	P(c)	10		Blackman and Eastop 1984 [?]
Eriosoma (Mimaphidus) patchiae (Börner & Blunck, 1916)	P(c)	10		Blackman 1980 [Great Britain]
Eriosoma (Schizoneura) auratum Akimoto, 1983	P(c)	12		Blackman 1986 [Japan]
Eriosoma (Schizoneura) grossulariae (Schüle, 1887)	P(c)	10		Blackman and Eastop 1984 [?]
Eriosoma (Schizoneura) harunire Akimoto, 1983	P(c)	10		Blackman 1986 [Japan]
Eriosoma (Schizoneura) japonicum (Matsumura, 1917)	P(c)	10		Blackman 1986 [Japan]
Eriosoma (Schizoneura) kashmiricum Ghosh, Verma & Raychaudhuri, 1976	P(c)	12		Pal and Khuda-Bukhsh 1983 [Garhwal, Uttarakhand, India]
? Eriosoma (Schizoneura) laciniatae Pashtshenko, 1988	P(c)	16		Blackman and Eastop 1994 [?]
Eriosoma (Schizoneura) longicornutum Akimoto, 1983	P(c)	10		Blackman 1986 [Japan]
Eriosoma (Schizoneura) moriokense Akimoto, 1983	P(c)	10		Blackman 1986 [Japan]
Eriosoma (Schizoneura) ulmi (Linnaeus, 1758)	P(c)	10		Blackman and Eastop 1984 [Europe]
		12		Baehr 1908, 1909 (as *Schizoneura*) [Germany]
Eriosoma near ulmi (Linnaeus, 1758)	?	16		Chen and Zhang 1985a [Beijing area, China] (cited after Blackman and Eastop 2015)
Eriosoma (Schizoneura) yangi Takahashi, 1939	P(c)	10		Blackman 1986 [Japan]
*Forda formicaria* von Heyden, 1837	P(c), P(o)	20		Robinson and Chen 1969a [Canada], Blackman and Spence 1996 [Great Britain]
		18-20 (somatic cells) or 21-23 (germline cells)		Blackman 1987a [Great Britain; Czechoslovakia; Sicily, Italy; Cyprus; Israel; Iran; USA; Canada]
		21, 22, 23		Blackman 1980 [Great Britain; North America] (anholocyclic populations)
*Forda hirsuta* Mordvilko, 1928	P(c), P(o)	18		Blackman 1980, 1987a [Iran]
*Forda marginata* Koch, 1857	P(c), P(o)	17-20 (somatic cells) or 25-40 (germ line cells)		Blackman 1987a [Great Britain; Sicily, Italy; Cyprus; Israel; Iran; USA; Canada]
		24,25, 26, 27, 32		Blackman 1980 [Great Britain; North America] (anholocyclic populations)
		28		Robinson and Chen 1969a [Canada]
*Forda riccobonii* (Stefani, 1899)	P(c)	18		Khuda-Bukhsh and Pal 1983b [Gharwal, Uttarakhand, India]
		30 (germ line cells)		Blackman 1980 (as *Forda dactylidis* Börner, 1950) [Iran] (but see Blackman 1987a: "Dr. V. F. Eastop has re-examined it and considers it to be closer to *Forda riccobonii* (Stefani)")
		18 (somatic cells) or 30 (germ line cells)		Blackman 1987a [Iran]
*Formosaphis micheliae* Takahashi, 1925	?P(o)	10		Blackman 1986 [Japan] (with structural heterozygosity)
*Geoica lucifuga* (Zehnter, 1897)	P(c), P(o)	14		Kulkarni 1984 [Darjeeling, West Bengal, India]
		18		Blackman and Eastop 1994 [?]
*Geoica?rungsi* Davatchi & Remaudière, 1957	P(c)	18		Blackman and Eastop 2015 [?] (holocyclic populations on *Pistacia atlantica*)
*Geoica setulosa* (Passerini, 1860)	P(c), P(o)	20?, 24, 28, 31		Blackman 1980 [Great Britain] (anholocyclic populations)
		20		Blackman 1980 [Iran]
		20, 24		Blackman and Eastop 2015 [?] (from grass roots)
*Geoica utricularia* (Passerini, 1856)	P(c), P(o)	16, 17, 18?		Blackman 1980 (as *Geoica eragrostidis* (Passerini, 1860)) [Great Britain] (anholocyclic populations)
		18		Blackman 1980 (as *Geoica eragrostidis* (Passerini, 1860)) [Italy]*
*Geoica?wertheimae* Brown & Blackman, 1994	P(c)	18		Blackman and Eastop 2015 [?] (holocyclic populations on *Pistacia palaestina*)
*Geoica* sp.	?	18		Blackman 1980 [Israel]
*Gootiella tremulae* Tullgren, 1925	P(c), ?P(o)	16		Blackman and Eastop 2015 [?]
*Hemipodaphis persimilis* Akimoto, 1983	P(c)	36		Blackman 1986 [Japan]
*Kaltenbachiella elsholtriae* (Shinji, 1936)	P(c)	32		Blackman 1986 [Japan]
*Kaltenbachiella japonica* (Matsumura, 1917)	P(c)	16/15	XX/X0	Blackman 1986 [Japan]
		18/?		Akimoto 1985 [Japan]
*Kaltenbachiella pallida* (Haliday, 1838)	P(c)	28		Blackman 1980 [Great Britain]
*Kaltenbachiella spinosa* Akimoto, 1985	P(c)	18		Akimoto 1985 [Japan]
*Melaphis rhois* (Fitch, 1866)	P(c), P(o)	26		Blackman and Eastop 1994 [?]
*Mordwilkoja vagabunda* (Walsh, 1863)	P(c), ?P(o)	20		Harper and MacDonald 1966, Robinson and Chen 1969a [Canada]
*Neoprociphilus aceris* (Monell, 1882)	P(c), P(o)	14		Robinson and Chen 1969a [Canada]
*Pachypappa marsupialis lambersi* Aoki, 1976	P(c)	10		Blackman 1986 [Japan]
*Pachypappa rosettei* (Maxson, 1934)	P(c)	10		Robinson and Chen 1969a (as *Asiphum*) [Canada]
*Pachypappa sacculi* (Gillette, 1914)	P(c)	10		MacDonald and Harper 1965 (as *Asiphum*), Harper and MacDonald 1966 (as *Asiphum*) [Canada]
*Pachypappa tremulae* (Linnaeus, 1761)	P(c)	10		Kuznetsova and Shaposhnikov 1973 (as *Asiphum*) [St. Petersburg, Russia]
*Pachypappa warshavensis* (Nasonov, 1894)	P(c)	10		Blackman and Eastop 1994 [?]
*Pachypappa* sp.	?	10		Blackman 1980 (as *Asiphum*) [Iran] (from *Populus euphratica*)
*Paracletus cimiciformis* von Heyden, 1837	P(c), P(o)	16		Blackman 1980 [Israel]
*Paracolopha morrisoni* (Baker, 1919)	P(c), ?P(o)	10/8	2(X_1_X_2_)/X_1_X_2_0	S. Akimoto, personal communication in Blackman 1986 (as *Colopha moriokaensis* (Monzen, 1923) [Japan], Blackman and Eastop 1994 [?]
*Patchiella reaumuri* (Kaltenbach, 1843)	P(c), P(o)	12		Colling, 1955 (as *Pachypappella*) [Great Britain]
*Pemphigus borealis* Tullgren, 1909	P(c)	20		Blackman and Eastop 1994 [?]
*Pemphigus bursarius* (Linnaeus, 1758)	P(c), P(o)	20		Baehr 1908, 1909 (as *Pemphigus pyriformis*) [Germany]
*Pemphigus dorocola* Matsumura, 1917	P(c)	20		Blackman 1986 [Japan]
*Pemphigus fuscicornis* (Koch, 1857)	P(o), ?P(c)	20/19	XX/X0	Kuznetsova and Shaposhnikov 1973 [Kiev, Ukraine], Kuznetsova 1974 [?]
*Pemphigus immunis* Buckton, 1896	P(c)	10		Pal and Khuda-Bukhsh 1982 [Srinagar, Jammu and Kashmir, India]
		20		Blackman and Eastop 2015 [?]
Pemphigus ? laurifolia Dolgova, 1973	P(c)	20		Blackman 1986 [Japan]
*Pemphigus matsumurai* Monzen, 1929	P(c)	12		Chen and Zhang 1985a [Beijing area, China] (cited after Blackman and Eastop 2015), Blackman 1986 [Japan], Blackman and Eastop 1994 [?] ("an unusual chromosome number for a *Pemphigus*, confirmed for Japanese samples from *Thalictrum*").
*Pemphigus microsetosus* Aoki, 1975	P(c)	22		Blackman 1986 [Japan]
*Pemphigus mordwilkoi* Cholodkovsky, 1912	P(c)	20		Dutta and Gautam 1993 [Shimla, Himachal Pradesh, India], Blackman and Eastop 1994 [?]
*Pemphigus passeki* Börner, 1952	P(c)	22		Gut 1976 [Holland]
*Pemphigus populicarius* Fitch, 1859	P(c)	20		MacDonald and Harper 1965 [Canada]
*Pemphigus populinigrae* (Schrank, 1801)	P(c), ?P(o)	22		Gut 1976 (as *Pemphigus filaginis* (Boyer de Fonscolombe, 1841) [Holland]
*Pemphigus populitransversus* Riley, 1879	P(c), ?P(o)	20		Harper and MacDonald 1966 [Canada]
*Pemphigus spyrothecae* Passerini, 1856	P(c)	20		Baehr 1909 [Germany]
*Pemphigus tartareus* Hottes & Frison, 1931	P(c)	20		Robinson and Chen 1969a (as *Pemphigus junctisensoriatus* Maxson, 1934) [Canada]
*Pemphigus* sp.	?	20		Blackman 1980 [USA] (from roots of *Euphorbia supina*)
*Prociphilus micheliae* Hille Ris Lambers, 1933	?	14		Kar et al. 1990 [India]
*Prociphilus osmanthae* Essig & Kuwana, 1918	P(c)	18		Khuda-Bukhsh and Kar 1990 [Shillong, Meghalaya, India]
Prociphilus (Meliarhizophagus) fraxinifolii (Riley, 1879)	P(c)	20		Robinson and Chen 1969a [Canada]
		22		Blackman and Eastop 1994 [?]
Prociphilus (Paraprociphilus) baicalensis (Cholodkovsky, 1920)	P(o), ?P(c)	12		Blackman 1986 [Japan], Blackman and Eastop 1994 [?]
Prociphilus (Paraprociphilus) tessellatus (Fitch, 1851)	P(c)	6		Blackman and Eastop 1994 [?]
Prociphilus (Stagona) konoi Hori, 1938	P(c)	18		Blackman 1986 [Japan], Blackman and Eastop 1994 [?]
Prociphilus (Stagona) pini (Burmeister, 1835)	P(c)	16		Blackman 1980 [Great Britain]
Prociphilus (Stagona) xylostei (De Geer, 1773)	P(c)	10		Pal and Khuda-Bukhsh 1983 [Garhwal, Uttarakhand, India], Blackman and Eastop 2015 [Europe]
*Prociphilus* sp. 1	?	18		Khuda-Bukhsh and Kar 1990 [Shillong, Meghalaya, India]
*Prociphilus* sp. 2	?	10		Dutta and Gautam 1993 [Shimla, Himachal Pradesh, India]
*Rectinasus buxtoni* Theobald, 1914	P(c), P(o)	26		Blackman 1980 [Iran]
*Schlechtendalia chinensis* (Bell, 1851)	P(c)	c. 36		Blackman and Eastop 1994 [?]
*Smynthurodes betae* Westwood, 1849	P(c), P(o)	8		Blackman 1980 [Great Britain; Iran]
*Tetraneura radicicola* Strand, 1929	P(c), ?P(o)	14		Blackman and Eastop 1984 [?]
		14/13	XX/X0	S. Akimoto, personal communication in Blackman 1986 [Japan]
*Tetraneura ulmi* (Linnaeus, 1758)	P(c), P(o)	14/13	XX/X0	Schwartz 1932 [Munich, Germany]
		14, 16		Galli and Manicardi 1998 (gall generation) [Italy]
		16		Blackman and Eastop 1984 [?]
*Tetraneura yezoensis* Matsumura, 1917	P(c), P(o)	12/11	XX/X0	S. Akimoto, personal communication in Blackman 1986 [Japan]
		12		Blackman and Eastop 1994 [Japan]
		18		Chen and Zhang 1985a [Beijing area, China] (cited after Blackman and Eastop 2015)
Tetraneura (Tetraneurella) fusiformis Matsumura, 1917	P(c), P(o)	18/16	2(X_1_X_2_)/X_1_X_2_0	S. Akimoto, personal communication in Blackman 1986 [Japan]
		18		Blackman and Eastop 2015 [?] (gall generation)
		17, 18, 19, 20		Blackman and Eastop 2015 [?] (permanently parthenogenetic populations)
Tetraneura (Tetraneurella) nigriabdominalis (Sasaki, 1899)	P(c), P(o)	14		Kulkarni and Kacker 1981a (as *Tetraneura hirsuta* Baker) [Sukna, West Bengal, India], Kulkarni 1984 [Darjeeling, West Bengal, India]
		14, 15, 16		Gautam et al. 1993, Manicardi and Gautam 1994 (as *Tetraneura akinire*) [Modena, Italy]
		17		Blackman and Eastop 1984 [?] (one sample), Blackman and Eastop 1994 [?] (anholocyclic population)
		18		Chen and Zhang 1985a [Beijing area, China] (cited after Blackman and Eastop 2015), Blackman 1986 [Japan]
		13-19 with modal number 18	XX/X0	Galli and Manicardi 1998 (gall generation) [Italy]
		19		Blackman and Eastop 1994 [?] (anholocyclic population)
Tetraneura (Tetraneurella) sp. 1 prope nigriabdominalis (Sasaki, 1899)	?	24		Blackman 1986 [Japan]
Tetraneura (Tetraneurella) sp. 2 prope nigriabdominalis (Sasaki, 1899)	?	22, 26		S. Akimoto, personal communication in Blackman 1986 [Japan]
Tetraneura (Tetraneurella) sorini Hille Ris Lambers, 1970	P(c)	16/14	2(X_1_X_2_)/X_1_X_2_0	S. Akimoto, personal communication in Blackman 1986 [Japan]
*Tetraneura* sp.	?	10		Chen and Zhang 1985a [Beijing area, China] (cited after Blackman and Eastop 2015)
*Thecabius affinis* (Kaltenbach, 1843)	P(c), P(o)	28		MacDonald and Harper 1965 (as *Thecabius populiconduplifolius* (Cowen, 1895)), Harper and MacDonald 1966 (as *Thecabius populiconduplifolius*) [Canada]
		38		Blackman 1980 [Great Britain], Blackman 1986 (as *Thecabius orientalis* (Mordvilko, 1935)) [Japan], Blackman and Eastop 2006 [British Columbia, Canada]
Tetraneura (Parathecabius) auriculae (Murray, 1877)	?	16		Blackman and Eastop 2006 [?]
Tetraneura (Parathecabius) latisensorius (Hori, 1938)	P(c)	18+1 (1 B-chromosome?)		Blackman 1986 (as *Thecabius*) [Japan]
Tetraneura (Parathecabius) lysimachiae Börner, 1916	P(c), P(o)	18		Gut 1976 [Holland]
**Fam. Mindaridae**				
*Mindarus abietinus* Koch, 1857	P(c)	12		Robinson and Chen 1969a [Canada], Blackman and Eastop 1994 [Europe]
*Mindarus obliquus* (Cholodkovsky, 1896)	P(c)	8		Blackman and Eastop 1994: [?] (sample from *Parathecabius glauca* in British Columbia, Canada (leg. C.K. Chan) had 2n=8 (R.L. Blackman; unpublished data), indicating that there may be more than one species on *Picea* in Canada)
		12		Robinson and Chen 1969a [Canada]
**Fam. Lachnidae**				
*Cinara atlantica* (Wilson, 1919)	P(c), P(o)	10		Blackman and Eastop 1994 [?]
*Cinara atrotibialis* David & Rajasingh, 1968	?P(o)	10		Khuda-Bukhsh and Kar 1990 [Shillong, Meghalaya, India]
		22		Das et al. 1985 [India]
*Cinara braggii* (Gillette, 1917)	P(c)	10		Sun and Robinson 1966, Robinson and Chen 1969a [Canada]
*Cinara cedri* Mimeur, 1936	P(c)	10		Blackman and Eastop 1994 [?]
*Cinara cembrae* (Seitner, 1936)	P(c)	10		Rukavischnikov 1979 [Siberia, Russia]
*Cinara coloradensis* (Gillette, 1917)	?	10		Robinson and Chen 1969a [Canada]
*Cinara confinis* (Koch, 1856)	P(c), P(o)	12		Blackman and Eastop 1994 [?]
*Cinara costata* (Zetterstedt, 1828)	P(c)	10		Blackman and Eastop 1994 [?]
*Cinara cronartii* Tissot & Pepper, 1967	?P(o)	10		Blackman and Eastop 1994 [?]
*Cinara cuneomaculata* (del Guercio, 1909)	P(c)	10/9		Shinji 1931 (as *Dilachnus laricis* (Walker, 1848)) [Japan]
*Cinara formosana* (Takahashi, 1924)	P(c)	10		Blackman and Eastop 1994 [?]
*Cinara fornacula* Hottes, 1930	P(c)	10		Robinson and Chen 1969a [Canada]
*Cinara hyperophila* (Koch, 1855)	P(c)	10		Rukavischnikov 1974, 1979 [Novosibirsk, Russia]
*Cinara kochiana* (Börner, 1939)	P(c)	10		Rukavischnikov 1974, 1979 (as *Cinara boerneri* Hille Ris Lambers, 1956 - see Mamontova 1991) [Novosibirsk, Russia], Blackman 1980 [Great Britain]
*Cinara lachnirostris* Hille Ris Lambers, 1966	?	8		Dutta and Gautam 1993 [Shimla, Himachal Pradesh, India]
*Cinara laricicola* (Matsumura, 1917)	P(c)	10		Shinji 1927 (as *Dilachnus laricolus*), 1931, 1941a (as *Cinara laricis*), Blackman 1986 [Japan]
*Cinara laricifex* (Fitch, 1858)	P(c)	10		Robinson and Chen 1969a [Canada]
*Cinara laricis* Hartig 1839	P(c)	10		Rukavischnikov 1979 [Siberia, Russia]
*Cinara maculipes* Hille Ris Lambers, 1966	P(c)	12		Das et al. 1985 [Jammu and Kashmir, India], Kurl and Chauhan 1986a, 1987a [Chail, Himachal Pradesh, India]
*Cinara maghrebica* Mimeur, 1934	?	10		Blackman and Eastop 1994 [?]
*Cinara matsumurana* Hille Ris Lambers, 1966	P(c)	10		Blackman 1986 [Japan]
*Cinara nuda* Mordvilko, 1895	P(c)	10		Rukavischnikov 1974, 1979 [Novosibirsk, Russia]
*Cinara palaestinensis* Hille Ris Lambers, 1948	?P(o)	10		Blackman and Eastop 1994 [?]
*Cinara pectinatae* (Nördlinger, 1880)	P(c)	6		Blackman and Eastop 1994 [Germany] (2 samples) (about record of Rukavishnikov 1979 see *Cinara confinis* (Koch, 1856))
*Cinara pergandei* (Wilson, 1919)	P(c)	14		Blackman 1990 [?], Blackman and Eastop 1994 [?]
*Cinara piceae* (Panzer, 1801)	P(c)	10		Rukavischnikov 1979 (as *Cinara piceae* (Panzer, 1801) and also as misidentification of *Cinara pectinatae* (Nördlinger, 1880)) – see Mamontova 1991) [Novosibirsk, Russia], Blackman and Eastop 1994 [Great Britain]
*Cinara piceicola* (Cholodkovsky, 1896)	P(c)	8		Blackman 1990 [?], Blackman and Eastop 1994 [?]
*Cinara pilicornis* (Hartig, 1841)	P(c)	10		Blackman 1990, Blackman and Eastop 1994 [Great Britain; New Zeland]
		14		Rukavischnikov 1974, 1979 [Novosibirsk, Russia]
*Cinara pilosa* (Zetterstedt, 1840)	?	8		Blackman 1990 [?], Blackman and Eastop 1994 [?] (Mamontova 2001 noted 2n=14 accoring to Rukavischnikov 1979, but the last paper does not consider *Cinara pilosa* in reality)
*Cinara pinea* (Mordvilko, 1895)	P(c)	10		Sun and Robinson 1966, Robinson and Chen 1969a [Canada]
		10, 11, 14		Blackman 1990 [Great Britain]
		14		Rukavischnikov 1974, 1979 [Novosibirsk, Russia]
*Cinara pini* (Linnaeus, 1758)	P(c)	10/9	XX/X0	Rukavischnikov 1974, 1979 (as *Cinara pini* (Linnaeus, 1758) and as *Cinara hyperophila* (Koch, 1855) – see Mamontova 1991) [Novosibirsk, Russia], Blackman 1986 [Europe]
*Cinara pinidensiflorae* (Essig & Kuwana, 1918)	P(c)	10		Blackman and Eastop 2015 [?]
		22/21	XX/X0	Shinji 1931 (as *Dilachnus*), Blackman 1986 [Japan] (based on n(♂) = 11(Shinji 1931))
*Cinara piniformosana* (Takahashi, 1923)	P(c)	10		Blackman 1986 [Japan]
*Cinara pinimaritimae* (Dufour, 1833)	P(c), ?P(o)	16		Blackman 1990, Blackman and Eastop 1994 (as *Cinara maritimae*) [?]
*Cinara ponderosae* (Williams, 1911)	P(c), P(o)	10		Blackman 1980 [USA]
*Cinara pruinosa* (Hartig, 1841)	P(c), P(o)	10		Blackman and Eastop 1994 [?]
*Cinara schimitscheki* Börner, 1940	P(c)	10		Blackman and Eastop 1994 [?]
*Cinara similis* (van der Goot, 1917)	?	12		Kulkarni and Kacker 1981a (as *Lachnus*) [Dadhau, Himachal Pradesh, India]
*Cinara strobi* (Fitch, 1851)	P(c)	10		Blackman and Eastop 1994 [?]
*Cinara tenuipes* Chakrabarti & Ghosh, 1974	?	12		Pal and Khuda-Bukhsh 1982 (as *Cinara abieticola tenuipes* Chakrabarti and Ghosh) [Srinagar, Jammu and Kashmir, India] (probably misidentification – aphids were collected from unusual host plant, *Juniperus communis*)
Cinara (Cupressobium) cupressi (Buckton, 1881)	P(c), P(o)	12		Blackman 1980 [Great Britain]
Cinara (Cupressobium) fresai Blanchard, 1939	P(o)	13		Blackman 1980 [Great Britain]
Cinara (Cupressobium) juniperi (de Geer, 1773)	P(c)	12		Blackman 1980 [Great Britain]
Cinara (Cupressobium) louisianensis Boudreaux, 1949	?	12		Blackman 1990 [?]
Cinara (Cupressobium) tujafilina (del Guercio, 1909)	P(c)	12		Blackman 1980 [USA; Iran], Das et al. 1985 [India], Dutta and Gautam 1993 [Shimla, Himachal Pradesh, India]
*Essigella californica* (Essig, 1909)	P(c), P(o)	8		Blackman 1980 [USA]
*Eulachnus agilis* (Kaltenbach, 1843)	P(c)	8		Rukavischnikov 1979 (as *Protolachnus*) [Novosibirsk, Russia], Blackman 1980 [Great Britain; Sweden]
*Eulachnus brevipilosus* Börner, 1940	?P(o)	30		Blackman 1980 [Great Britain]
*Eulachnus rileyi* (Williams, 1911)	P(c), ?P(o)	8		Blackman 1980 [USA; Iran]
*Eulachnus thunbergii* (Wilson, 1919)	P(c)	8		Khuda-Bukhsh and Kar 1990 [Shillong, Meghalaya, India]
		14/13	XX/X0	Shinji 1927, 1931 (as *Eulachnus piniformosanus* Takahashi, 1931), Blackman 1986 [Japan] (based on n(♂) = 7 (Shinji 1927, 1931))
*Eulachnus tuberculostemmatus* (Theobald, 1915)	?	8		Blackman 1986 [Europe], Khuda-Bukhsh and Kar 1990 (cited after Blackman and Eastop 2015)
*Lachnus acutihirsutus* Kumar & Burkhardt, 1970	?	16		Dutta and Gautam 1993 [Shimla, Himachal Pradesh, India]
*Lachnus longirostris* (Mordvilko, 1909)	P(c)	8		Blackman 1990 (as *Lachnus iliciphilus*) [West Germany]
*Lachnus roboris* (Linnaeus, 1758)	P(c)	7?	2(X_1_X_2_)/ X_1_X_2_0	Blackman 1990 [West Germany]
		8 (7+1B)		Blackman 1990 [Czechoslovakia; West Germany]
		9 (7+2B)		Blackman 1990 [Czechoslovakia; Denmark; Poland]
		10		Blackman 1990 [Portugal; Great Britain?]
		11 (10+1B)		Blackman 1990 [Sweden; Great Britain]
		12?		Blackman 1990 [Portugal]
		14		Blackman 1990 [Great Britain]
		15 (13+ 2B?), 16, 17?		Blackman 1990 [Portugal]
		7, 8, 10, 11, 13, 16 and17		Blackman and Eastop 2015 [?] (some of these may apply to *Lachnus iliciphilus*; a sample from *Castanea* in Portugal had 2n=10 (Blackman 1990))
*Lachnus tropicalis* (van der Goot, 1916)	P(c), ?P(o)	10	XX/X0	Shinji 1927, 1941a (as *Pterochlorus*), Blackman 1986 [Japan] (based on n(♂) = 5 (Shinji 1927))
		16		Shinji 1931 (as *Pterochlorus*) [Japan], Blackman 1986 [Japan] (based on n(♂) = 8 (Shinji 1931))
		12, 13 or 16		Blackman 1986, 1990 [Japan; China]
		12, 14, 16, 18, 22, 28, 38		Muramoto 1987 [Japan] (Blackman and Eastop 2015: " Muramoto (1987) reported chromosome numbers from 2n=14-38, but his results are difficult to interpret and may include polyploid cells and/or preparations of more than one species.")
*Maculolachnus sijpkensi* Hille Ris Lambers, 1962	P(c)	10		Robinson and Chen 1969a [Canada]
*Maculolachnus submacula* (Walker, 1848)	P(c)	10		Blackman 1980 [Great Britain], Blackman and Spence 1996 [Great Britain]
		10/9	XX/X0	Blackman 1990 [Great Britain]
*Protrama flavescens* (Koch, 1857)	P(o)	40-42, c. 42		Blackman 1980 [Great Britain]
		~ 42, 42		Blackman et al. 2000 [Great Britain]
*Protrama radicis* (Kaltenbach, 1843)	P(o)	c.60		Blackman 1980 [Great Britain]
		~ 50		Blackman et al. 2000 [Great Britain]
*Protrama ranunculi* (del Guercio, 1909)	?	c.36		Blackman 1980 [Great Britain]
*Pterochloroides persicae* (Cholodkovsky, 1899)	P(c), P(o)	20		Blackman and Eastop 1984 [?], Blackman 1990 [?]
*Schizolachnus pineti* (Fabricius, 1781)	P(c), ?P(o)	10		Blackman 1980 [Great Britain]
		18		Rukavischnikov 1974, 1979 [Novosibirsk, Russia] (Blackman and Eastop 2015 supposed that the material from Novosibirsk may be misidentification of *Schizolachnus obscurus*)
*Stomaphis bratislavensis* Czylok & Blackman, 1991	P(c)	8		Blackman 1990 (as *Stomaphis quercus* (Linnaeus, 1758)) [Czechoslovakia], Czylok and Blackman 1991 [Slovakia]
*Stomaphis cupressi* (Pintera, 1965)	?	14		Blackman 1990 [?]
*Stomaphis japonica* Takahashi, 1960	P(c)	10/8	2(X_1_X_2_)/ X_1_X_2_0	Blackman 1986 [Japan], Blackman 1990 [?], Czylok and Blackman 1991 [Japan]
*Stomaphis quercus* (Linnaeus, 1758)	P(c)	10/8	2(X_1_X_2_)/ X_1_X_2_0	Blackman 1990 [Europe]
*Stomaphis yanonis* Takahashi, 1918	P(c)	15, 16?	2(X_1_X_2_)/X_1_X_2_0	Blackman 1990 [?]
		20?	2(X_1_X_2_)/ X_1_X_2_0	Honda 1921 (as *Stomaphis janonis*), Blackman 1986 [Japan] (based on n(♂) = 10 (Honda 1921))
*Trama rara* Mordvilko, 1908	?	12		Blackman et al. 2000 [Great Britain]
		12, 13,14		Normark 1999 [Great Britain; Poland]
		13		Blackman 1980 [Great Britain]
*Trama troglodytes* von Heyden, 1837	P(o), P(c)	13, 14, 16, 17, 18, 19, 20, 21, 23		Normark 1999 [Great Britain; France; Germany; Czech Republic; Poland]
		14, 15, 16, 17, 18, 19, 20, 21, 22		Blackman 1980 [Great Britain]
		14, 15, 17, 18, 19, 20, 21,22, 23		Blackman et al. 2000 [Great Britain]
		16		Blackman et al. 2000 [Poland]
		16 (colony without sexual morphs), 20 (colonie swith sexual morphs)		Blackman et al. 2001 [Great Britain]
		21		Blackman and Spence 1996 [Great Britain]
Trama (Neotrama) caudata del Guercio, 1909	P(o)	9, 11		Blackman 1980 (as *Neotrama*) [Great Britain]
		9, 10, 11,12		Blackman et al. 2000 [Great Britain]
		10, 12		Normark 1999 [Great Britain]
Trama (Neotrama) maritima (Eastop, 1953)	P(o)	10, 11, 12, 13, 14		Normark 1999, Blackman et al. 2000 [Great Britain]
*Tuberolachnus salignus* (Gmelin, 1790)	P(o)	8		Morgan 1909b (as *Lachnus dentatus* Le Baron, 1872) [USA], Shinji 1927, 1931, 1941a (as *Tuberolachnus viminalis* (Fonscolombe)) [?] (based on n(♂) = 4 (Shinji 1927, 1931, 1941a), but Blackman 1980 supposed that all these data are misidentifications of different species of *Pterocomma*).
		20		Blackman 1986 [Japan], Blackman 1990 [Great Britain; Iran; India; Japan], Blackman and Spence 1996 [Great Britain]
		18,19,20		Dhatwalia and Gautam 2009 [Himachal Pradesh, India]
		22		Raychaudhuri and Das 1987 [India]
**Fam. Hormaphididae**				
*Aleurodaphis asteris* Takahashi & Sorin, 1958	P(o)	32		Blackman 1986 [Japan]
*Aleurodaphis impatientis* Sorin & Miyazaki, 2004	P(o)	c.30		Blackman and Eastop 2006 [?]
*Aleurodaphis mikaniae* Takahashi, 1925	?	c.30		Blackman 1986 [Japan]
*Astegopteryx bambusae* (Buckton, 1893)	?	12		Kar et al. 1990 [India]
*Aleurodaphis formosana* (Takahashi, 1924)	?	12		Chen and Zhang 1985b (as *Aleurodaphis insularis*) (cited after Blackman and Eastop 2015)
*Aleurodaphis himalayensis* (M.R. Ghosh, Pal & D.N. Raychaudhuri, 1977)		12		Kar et al. 1990 (as *Pseudoastegopteryx*) [India]
*Aleurodaphis minuta* (van der Goot, 1917)	?	12		Kar et al. 1990 [India]
*Cerataphis brasiliensis* (Hempel, 1901)	P(c), P(o)	18		Blackman and Eastop 2006 [?]
*Cerataphis orchidearum* (Westwood, 1879)	P(o)	16		Blackman and Eastop 1984, 2006 [?] (samples from *Cymbidium*, *Dendrobium* and *Epidendrum*)
		18		Blackman and Eastop 1984, 2006 [?] (samples from *Angraecum*, *Sarcochilus* and *Butia*)
*Ceratoglyphina bambusae* van der Goot, 1917	P(c)	12		Chen and Zhang 1985a [Beijing area, China] (cited after Blackman and Eastop 2015)
*Ceratoglyphina bengalensis* L.K. Ghosh, 1972	?	12		Khuda-Bukhsh and Kar 1987 (as *Ceratoglyphina bambusae bengalensis* Ghosh) [Kalimpong, West Bengal, India]
*Ceratovacuna indica* M.R. Ghosh, Pal & D.N. Raychaudhuri, 1977	?	12		Kar et al. 1990 [India]
*Ceratoglyphina japonica* (Takahashi, 1924)	P(c), P(o)	12		Blackman 1986 [Japan]
*Ceratoglyphina lanigera* Zehntner, 1897	P(o)	12		Blackman and Eastop 1984 [?], Blackman 1986 [Japan], Kar et al. 1990 [India]
*Ceratoglyphina nekoashi* (Sasaki, 1910)	P(c)	12		Blackman 1986 [Japan]
*Ceratoglyphina perglandulosa* R.C. Basu, A.K. Ghosh & D.N. Raychaudhuri, 1975	?	12		Khuda-Bukhsh and Kar 1987 [Kalimpong, West Bengal, India]
*Ceratoglyphina silvestrii* (Takahashi, 1927)	?	8		Kurl 1980b [Meghalaya, India]
		12		Khuda-Bukhsh and Kar 1987 [Kalimpong, West Bengal, India]
*Euthoracaphis umbellulariae* (Essig, 1932)	?P(c), P(o)	14		Blackman 1980 [USA]
*Hamamelistes betulinus* (Horvath, 1896)	P(o), P(c)	12		Kuznetsova and Shaposhnikov 1973 (as *Tetraphis*) [St. Petersburg, Russia], Blackman 1986 [Japan], Blackman and Eastop 2015 [?] (for anholocyclic European population)
*Hamamelistes spinosus* Shimer, 1867	P(c)	c. 50		Blackman 1980 [Canada], Blackman and Eastop 1994 [?]
*Hormaphis betulae* (Mordvilko, 1901)	P(o), P(c)	?18		Blackman 1986 [Japan]
*Hormaphis cornu* (Shimer, 1867)	P(c)	?18		Blackman and Eastop 1994 [?]
*Hormaphis hamamelidis* (Fitch, 1851)	P(c)	?18		Blackman and Eastop 1994 [?]
*Pseudoregma alexanderi* (Takahashi, 1924)	P(o), ?P(c)	12		Khuda-Bukhsh and Kar 1987 (as *Paraoregma*) [Kalimpong, West Bengal, India]
*Pseudoregma bambucicola* (Takahashi, 1921)	P(c), P(o)	12		Blackman 1986 [Japan], Chen and Zhang 1985b (cited after Blackman and Eastop 2015), Khuda-Bukhsh and Kar 1987 [Kalimpong, West Bengal, India]
*Pseudoregma panicola* (Takahashi, 1921)	P(o)	12		Blackman 1986 [Japan]
*Thoracaphis* sp.	?	12		Blackman 1980 [Japan]
**Fam. Thelaxidae**				
*Glyphina betulae* (Linnaeus, 1758)	P(c)	10/9	XX/X0	Kuznetsova and Shaposhnikov 1973 [St.Petersburg, Russia]
		28/27, 56/55	XX/X0	Blackman 1989 [Poland; Great Britain; Lithuania]
*Glyphina jacutensis* Mordvilko, 1931	P(c)	8		Blackman 1989 [Romania; Lithuania]
		10		Kuznetsova and Shaposhnikov 1973 (as *Glyphina schrankiana* Börner, 1950) [St.Petersburg, Russia]
*Glyphina pseudoschrankiana* Blackman, 1989	P(c)	10/9	XX/X0	Blackman 1989 [Great Britain, Sweden]
*Glyphina* sp. from *Betula*	?	55		Blackman 1980 [Great Britain]
*Kurisakia onigurumii* (Shinji, 1923)	?P(c)	18		Blackman and Eastop 1994 [?] (or specimens from *Pterocarya stenoptera* in China)
*Thelaxes californica* (Davidson, 1919)	P(c)	12		Blackman and Eastop 1994 [?]
*Thelaxes dryophila* (Schrank, 1801)	P(c)	8		Kuznetsova and Shaposhnikov 1973 [St.Petersburg, Russia], Kuznetsova 1974 [?]
*Thelaxes suberi* (del Guercio, 1911)	?	8		Blackman and Eastop 1984 [?]
*Thelaxes valtadorosi* Remaudière, (1982) 1983	?	8		Blackman and Eastop 1994 [?]
**Fam. Aiceonidae**				
*Aiceona retipennis* David, Narayanan & Rajasingh, (1970) 1971	?	18		Khuda-Bukhsh 1980 [Garhwal, Uttarakhand, India]
**Fam. Anoeciidae**				
*Anoecia corni* (Fabricius, 1775)	P(c), P(o)	6		Blackman and Eastop 2015 [?]
		6, 7, 8 (rear-rangements, hybri-dization?)		Blackman 1980 [Great Britain; Iran]
		8		Dutta and Gautam 1993 [Shimla, Himachal Pradesh, India]
*Anoecia cornicola* (Walsh, 1863)	P(c), P(o)	10		Robinson and Chen 1969a (as *Anoecia querci* Fitch, 1859) [Canada]
*Anoecia furcata* (Theobald, 1915)	P(o), ?P(c)	12		Gautam et al. 1993 [Modena, Italy]
		12, 13		Blackman 1980 (as *Anoecia furcata* (Theobald, 1915) and as *Anoecia nemoralis* Börner, 1950) [Great Britain]
*Anoecia graminis* Gillette & Palmer, 1924	P(c)	8		Sun and Robinson 1966, Robinson and Chen 1969a [Canada]
*Anoecia haupti* Börner, 1950	P(c)	8		Blackman and Eastop 2015 [?]
*Anoecia major* Börner, 1950	P(c)	7		Blackman 1980 [Great Britain] (2n=7 in possible hybrids with *corni*), Blackman and Eastop 2006 [?]
		8		Blackman 1980 [Great Britain], Blackman and Eastop 2006 [?]
*Anoecia vagans* (Koch, 1856)	P(c)	12		Blackman 1980 [Great Britain; Sweden]
Anoecia sp. prope haupti Börner, 1950	?	8		Kuznetsova and Shaposhnikov 1973 [Crimea, Ukraine], Kuznetsova 1974 [?]
**Fam. Phloeomyzidae**				
*Phloeomyzus passerinii* Signoret, 1875	P(c), P(o)	10		Gut 1976 [Holland]
**Fam. Greenideidae**				
*Anomalosiphum indigoferae* A.K. Ghosh, M.R. Ghosh & D.N. Raychaudhuri, 1971	?	18		Blackman 1980 [Sarawak, Malaysia]
*Cervaphis quercus* Takahashi, 1918	?	8		Kurl 1980b [Meghalaya, India], Blackman 1986 [Japan]
*Cervaphis rappardi indica* A.N. Basu, 1961	?	8		Kar et al. 1990 [India]
*Eutrichosiphon heterotrichum* (Raychaudhuri, 1956)	P(c), P(o)	20		Blackman 1986 (as *Eutrichosiphon dubium*) [Japan] (see Blackman and Eastop 2015)
*Eutrichosiphon makii* Raychaudhuri & Chatterjee, 1974	?	40		Khuda-Bukhsh and Kar 1990 [Shillong, Meghalaya, India]
*Eutrichosiphon parvulum* Eastop & Hille Ris Lambers, 1976	?	26		Blackman and Eastop 2006 [?]
*Eutrichosiphum* sp.	?	20		Dutta and Gautam 1993 [Shimla, Himachal Pradesh, India]
*Greenidea ayyari* D.N. Raychaudhuri, M.K. Ghosh, Banerjee, A.K. Ghosh, 1973	?	18		Gautam and Kumar 2006 [Shimla, Himachal Pradesh, India]
*Greenidea ficicola* Takahashi, 1921	P(o)	22		Blackman 1980 [Australia]
*Greenidea longisetosa* Raychaudhuri, Ghosh, Banerjee & Ghosh, 1973	?	18		Khuda-Bukhsh and Kar 1990 [Shillong, Meghalaya, India]
*Greenidea mangiferae* Takahashi, 1925	?	20		Chen and Zhang 1985b (cited after Blackman and Eastop 2015)
*Greenidea querciphaga* Raychaudhuri, Ghosh, Banerjee & Ghosh., 1973	?	18		Gautam and Kumar 2006 [Shimla, Himachal Pradesh, India]
Greenidea (Trichosiphum) anonae (Pergande, 1906)	?P(o), ?P(c)	22		Khuda-Bukhsh and Kar 1990 [Shillong, Meghalaya, India]
Greenidea (Trichosiphum) bucktonis A.K. Ghosh, R.C. Basu & D.N. Raychaudhuri, 1970	?	8		Kar et al. 1990 (as Greenidea (Trichosiphum) schoutedeni Raychaudhuri, Ghosh, Banerjee and Ghosh) [India]
		14		Kapoor and Gautam 1994 [Shimla, Himachal Pradesh, India]
Greenidea (Trichosiphum) haldari Maity & Chakrabarti, 1980	?	20		Gautam and Kumar 2006 [Shimla, Himachal Pradesh, India]
Greenidea (Trichosiphum) heeri D.N. Raychaudhuri, M.R. Ghosh, M. Banerjee & A.K. Ghosh, 1973	?	7, 8, 9		Kurl 1986 (as Greenidea (Trichosiphum) formosana heeri D.N. Raychaudhuri, M.R. Ghosh, M. Banerjee & A.K. Ghosh, 1973) [Meghalaya, India]
Greenidea (Trichosiphum) kuwanai (Pergande, 1906)	?P(c)	20		Blackman 1980, 1986 [Japan], Gautam and Kumar 2006 [Shimla, Himachal Pradesh, India]
Greenidea (Trichosiphum) nipponica Suenaga, 1934	P(c)	18		Blackman 1986 [Japan]
Greenidea (Trichosiphum) psidii van der Goot, 1917	P(o)	18		Kulkarni and Kacker 1979 (as Greenidea (Trichosiphum) formosana formosana (Maki )[Rautara, West Bengal, India], Kar et al. 1990 (as Greenidea (Trichosiphum) formosana formosana (Makiischout))[India], Khuda-Bukhsh and Kar 1990 (as Greenidea (Trichosiphum) formosana formosana (Maki, 1917)) [Shillong, Meghalaya, India], Dutta and Gautam 1993 (as Greenidea (Trichosiphum) formosana (Maki, 1917)) [Mandi, Himachal Pradesh, India], Samkaria et al. 2010 (as *Greenidea formosana* (Maki)) [Palampur, Himachal Pradesh, India]
*Mollitrichosiphum nandii* A.N. Basu, 1964	P(c)	16		Blackman and Eastop 1994 [?]
*Schoutedenia ralumensis* Rübsaamen, 1905	P(c), P(o)	14 (male)		Blackman 1980 [Australia]
		14		Khuda-Bukhsh and Kar 1990 (as *Schoutedenia lutea* (van der Goot, 1917)) [Kalyani, West Bengal, India]
		15(sex unknown)		Blackman and Eastop 1994 [Papua New Guinea]
		16/14	2(X_1_X_2_)/ X_1_X_2_0	Hales 1989 (as *Schoutedenia lutea* (van der Goot)) [Australia]
**Fam. Drepanosiphidae**				
*Allaphis californica* (Hille Ris Lambers, 1974)	?	10		Blackman and Eastop 2006 (as *Thripsaphis*) [?]
*Allaphis foxtonensis* (Cottier, 1953)	?	10		Blackman and Eastop 2006 (as *Thripsaphis*) [?]
*Allaphis verrucosa* (Gillette, 1917)	P(c)	10		Blackman and Eastop 2006 (as *Thripsaphis*) [?]
*Betacallis alnicolens* Matsumura, 1919	?	22		Blackman 1986 [Japan]
*Betacallis odaiensis* Takahashi, 1961	?	22		Blackman 1986 [Japan] provided these data and supposed that "*Euceraphis betulifoliae*" in Shinji 1927 (with n=11) is very possibly *Betacallis odaiensis*.
*Betacallis sikkimensis* R.C. Basu, M.R. Ghosh & D.N. Raychaudhuri,1974	P(c)	20		Khuda-Bukhsh and Pal 1983b [Gharwal, Uttarakhand, India]
*Betulaphis brevipilosa* Börner, 1940	P(c)	20		Blackman and Eastop 1994 [?]
*Betulaphis pelei* Hille Ris Lambers, 1952	?	20		Blackman and Eastop 1994 [?]
*Betulaphis quadrituberculata* (Kaltenbach, 1843)	P(c)	20		Blackman 1980 [Sweden]
*Boernerina variabilis* Richards, 1961	P(c)	16		Blackman and Eastop 1994 [Canada]
*Calaphis arctica* Hille Ris Lambers, 1952	P(c)	18		Blackman and Eastop 1994 [?]
*Calaphis betulaecolens* (Fitch, 1851)	P(c)	20		Sun and Robinson 1966, Robinson and Chen 1969a [Canada]
*Calaphis betulella* Walsh, 1863	P(c)	18		Blackman 1980 [USA]
*Calaphis betulicola* (Kaltenbach, 1843)	P(c)	18		Gut 1976 [Holland]
*Calaphis coloradensis* Granovsky, 1939	P(c)	18		Blackman 1980 [USA]
*Calaphis flava* Mordvilko, 1928	P(c)	18		Gut 1976 [Holland], Blackman 1980 (as *Calaphis viridipallida* Palmer, 1952) [Canada]
*Calaphis leonardi* Quednau, 1971	P(c)	20		Blackman and Eastop 1994 [?]
*Calaphis magnoliae* (Essig & Kuwana, 1918)	?	8/7	XX/X0	Shinji 1927, 1931, 1941a (as *Chromaphis*), Blackman 1986 (as *Neocalaphis*) [Japan] (based on n(♂) = 4 (Shinji 1931))
		14/13		Shinji 1941a [Japan] (Blackman 1986 supposed that "Shinji must have had immature males of another species")
		20		Blackman 1986 [Japan]
*Calaphis magnolicolens* (Takahashi, 1921)	?	20/19	XX/X0	Shinji 1927, 1931, 1941a [Japan], Blackman 1986 (as *Neocalaphis*) [Japan] (their own data and based on n(♂) = 10 (Shinji 1927, 1931))
		20		Blackman 1986 [Japan]
*Calaphis viridipallida* Palmer, 1952	P(c)	18		Blackman 1980 [Canada]
*Calaphis* sp.	?	18/17	XX/X0	Shinji 1927, 1931, 1941a (as *Calaphis betulaecolens* Fitch, 1851) [USA]
*Callipterinella calliptera* (Hartig, 1841)	P(c)	20		Blackman 1980 [USA], Blackman 1986 [Japan]
*Callipterinella tuberculata* (von Heyden, 1837)	P(c)	20		Blackman 1980 [Great Britain]
*Chromaphis juglandicola* (Kaltenbach, 1843)	P(c)	8		Dutta and Gautam 1993 [Shimla, Himachal Pradesh, India]
*Chromocallis nirecola* (Shinji, 1933)	P(c)	18		Blackman and Eastop 1994 [?]
*Clethrobius comes* (Walker, 1848)	P(c)	11 (structural heterozygote)		Blackman 1986 [Japan], Blackman 1988 [Japan; Great Britain; Ireland; Finland]
*Ctenocallis israelica* Hille Ris Lambers, 1954	?	16		Blackman and Eastop 2006 [?]
*Ctenocallis setosa* (Kaltenbach, 1846)	P(c)	18		Blackman and Eastop 2006 [?]
*Drepanaphis acerifoliae* (Thomas, 1878)	P(c)	38		Shinji 1923 (as *Drefavaphis*) [USA]
		38/37	XX/X0	Shinji 1931 [USA]
*Drefavaphis simpsoni* Smith, 1959	?	30		Blackman and Eastop 1994 [?]
*Drefavaphis utahensis* Smith & Knowlton, 1943	?	30		Blackman 1980 [USA]
*Drepanosiphum braggii* Gillette, 1907	P(c)	30		Blackman 1980 [USA]
*Drepanosiphum iranicum* Hille Ris Lambers, 1971	P(c)	30		Blackman and Eastop 1994 [?]
*Drepanosiphum platanoidis* (Schrank, 1801)	P(c)	30		Shinji 1923 (as *Drefavosiphum flatavoides*) [USA]
		30/29	XX/X0	Shinji 1927, 1931, 1941a [USA]
*Eucalipterus tiliae* (Linnaeus, 1758)	P(c)	10		Blackman 1980 [Great Britain]
		10 (female), 8 (male)	2(X_1_X_2_)/ X_1_X_2_0	Blackman and Eastop 1994 [?]
		38-40?		Kuznetsova and Shaposhnikov 1973 [St.Petersburg, Russia]
*Euceraphis betulae* (Koch, 1855)	P(c)	10/8	2(X_1_X_2_)/X_1_X_2_0	Blackman 1976, 1977 [Great Britain], Blackman 1980 [Europe; West of North America], Blackman and Spence 1996 [Great Britain], Blackman and De Boise 2002 [Great Britain; New Zealand; USA]
		9, 10/7, 8		Blackman 1988 [Europe]
*Euceraphis betulae* group 1 (from *Betula papyrifera*)	?	7 (♀), 6 (♂)		Blackman 1980 [Northwest Territories, Canada], Blackman 1988 (Fig. 4b) [Northwest Territories, Yukon, Canada]
*Euceraphis betulae* group 2	?	8		Sun and Robinson 1966 (as *Euceraphis deducta* Baker, 1917), Robinson and Chen 1969a [Canada] (see comments in Blackman 1980)
*Euceraphis betulae* group 3	?	8 (+2) 2B-chro-mosomes		Blackman 1986 [Japan] (as *Euceraphis betulae*)
*Euceraphis betulijaponicae* (Matsumura, 1919)	P(c)	8 no B-chromo-somes		Blackman 1986 [Japan] (as *Euceraphis betulae*)
		8(+1) 1B-chro-mosome 2n (♂) = 6 (+1) n (♂) = 4 (+1)		Blackman 1986 [Japan] (as *Euceraphis betulae*)
		9/7		Blackman and De Boise 2002 [Japan]
*Euceraphis borealis* Blackman, 2002	P(c)	8/7	XX/X0	Blackman 1980 (as *Euceraphis betulae* group) [Northwest Territories, Manitoba, Canada], Blackman and De Boise 2002 [Canada] (one pair of X-chromosome)
*Euceraphis caerulescens* Pashtshenko, 1984	P(c)	22		Blackman 1986 (as *Euceraphis ontakensis* Sorin, 1970), Blackman and De Boise 2002 [Japan]
*Euceraphis gillettei* Davidson, 1915	P(c)	15, 16, 18		Blackman 1980 [Canada; USA]
		15, 16, 18, 19/13, 17		Blackman 1988, Blackman and De Boise 2002 [Canada; USA]
*Euceraphis lineata* Baker, 1917	P(c)	16		Blackman 1980 (also as *Euceraphis deducta* Baker, 1917) [USA]
		16/14		Blackman 1988, Blackman and De Boise 2002 [USA]
*Euceraphis mucida* (Fitch, 1856)	P(c)	20		Blackman 1980 [New York, Pennsylvania, USA]
		20, 21, 22/18, 19, 20		Blackman 1988, Blackman and De Boise 2002 [USA] (the differences are due to variation in the number of accessory ("B") chromosomes)
*Euceraphis ontakensis* Sorin, 1970	?	22		Blackman 1986, 1988 [Japan]
*Euceraphis papyrifericola* Blackman, 2002	P(c)	9/7		Blackman and De Boise 2002 [Canada]
		9-10/8		Blackman 1980 [USA; Canada] (as *Euceraphis betulae* group) (the 2n=8 male record was probably due to misinterpretation of B-chromosomes in somatic cells, as all later males examined had 2n=7 (i.e. 2 X-chromosomes) – Blackman, personal comm.)
*Euceraphis punctipennis* (Zetterstedt, 1828)	P(c)	7, 8, 9		Blackman 1980 [Great Britain]
		7, 8 (without or with B chromo- somes)		Blackman 1976 [Great Britain]
		7, 8/5, 6		Blackman 1988 [Europe]
		8/6		Blackman and De Boise 2002 [?]
		8		Blackman 1977 [?], Sun and Robinson 1966 [Canada]
*Euceraphis quednaui* Blackman, 2002	P(c)	11/9		Blackman 1980 [Utah, USA] (as *Euceraphis betulae* group), Blackman and De Boise 2002 [western USA] (including 3 "B" chromosomes)
*Hoplocallis picta* (Ferrari, 1872)	P(c)	14		Blackman and Eastop 2015 [?]
*Israelaphis carmini carmini* Essig, 1953	P(c)	18		Blackman 1980 (as *Israelaphis tavaresi* Ilharco, 1961) [Portugal]
*Israelaphis carmini alistana* Mier Durante, 1978	P(c)	18		Blackman 1980 (as *Israelaphis tavaresi alistana* Mier Durante, 1978) [Spain]
*Israelaphis lambersi* Ilharco, 1961	P(c)	16		Blackman and Eastop 2006 [?]
*Melanocallis caryaefoliae* (Davis, 1910)	P(c)	14		Blackman 1980 (as *Melanocallis fumipennellus* (Fitch)) [USA]
*Mesocallis sawashibae* (Matsumura, 1917)	P(c)	10		Blackman 1986 (as *Pterocallis*) [Japan]
Mesocallis (Paratinocallis) corylicola (Higuchi, 1972)	?	10		Blackman 1986 (as *Pterocallis*) [Japan]
*Monaphis antennata* (Kaltenbach, 1843)	P(c)	20		Blackman and Eastop 2015 [?]
*Monellia caryella* (Fitch, 1855)	P(c)	18		Blackman 1980 [USA]
*Monellia microsetosa* Richards, 1960	P(c)	18		Blackman 1980 [USA]
*Monelliopsis caryae* (Monell, 1879)	P(c)	18		Blackman 1980 [USA]
*Monelliopsis nigropunctata* (Granovsky, 1931)	P(c)	10		Blackman 1980 [Canada; USA]
*Myzocallis boerneri* Stroyan, 1957	P(c)	14		Blackman and Eastop 1994 [?]
*Myzocallis carpini* (Koch, 1855)	P(c)	14		Blackman and Eastop 1994 [?]
*Myzocallis coryli* (Goetze, 1778)	P(c)	14		Gut 1976 [Holland]
*Myzocallis glandulosa* Hille Ris Lambers, 1948	P(c)	14		Blackman and Eastop 1994 [?]
Myzocallis (Agrioaphis) castanicola Baker, 1917	P(c)	12/11		Shinji 1941a (as *Agrioaphis castanae*) [Japan]
		14	XX/X0	Kuznetsova et al. 1988 [St. Petersburg, Russia]
		14/13	XX/X0	Shinji 1923, 1927, 1931 (as *Myzocallis castaneae* (Fitch, 1857)) [USA]
Myzocallis (Agrioaphis) myricae (Kaltenbach, 1843)	P(c)	14		Gut 1976 [Holland]
Myzocallis (Neodryomyzus) polychaeta (David, 1969)	P(c)	12		Khuda-Bukhsh and Pal 1983b [Gharwal, Uttarakhand, India]
Myzocallis (Neomyzocallis) discolor (Monell, 1879)	P(c)	14		Robinson and Chen 1969a [Canada]
Myzocallis (Neomyzocallis) punctata (Monell, 1879)	P(c), P(o)	14		Sun and Robinson 1966, Robinson and Chen 1969a [Canada]
Myzocallis (Pasekia) cocciferina Quednau & Barbagallo, 1991	?P(o)	14		Blackman and Eastop 1994 [?]
Myzocallis (Pasekia) komareki (Pašek, 1953)	P(c)	14		Blackman and Eastop 1994 [?]
*Neochromaphis coryli* Takahashi, 1961	P(c)	18		Chen and Zhang 1985b (cited after Blackman and Eastop 2015)
*Neophyllaphis araucariae* Takahashi, 1937	P(o), ?P(c)	18		Hales and Lardner 1988 [Australia]
*Neophyllaphis brimblecombei* Carver, 1971	P(c)	26/25		Hales and Lardner 1988 [Australia]
*Neophyllaphis gingerensis* Carver, 1959	P(c)	14		Hales and Lardner 1988 [Australia]
*Neophyllaphis grobleri* Eastop, 1955	P(c)	18		Hales and Lardner 1988 [Africa], Blackman and Eastop 1994 [?]
*Neophyllaphis lanata* Hales & Lardner, 1988	P(c)	24/23		Hales and Lardner 1988 [Australia]
*Neophyllaphis podocarpi* Takahashi, 1920	P(c), ?P(o)	24		Chen and Zhang 1985b (cited after Blackman and Eastop 2015)
		26		Blackman 1986 [Japan]
*Neophyllaphis totarae* Cottier, 1953	P(c)	10		Hales and Lardner 1988 [New Zealand], Blackman and Eastop 1994 [?]
*Neuquenaphis bulbicauda* Hille Ris Lambers, 1968	?	14	XX/X0	Blackman et al. 2003 [Chile]
*Neophyllaphis edwardsi* (Laing, 1927)	P(c)	12	XX/X0	Blackman et al. 2003 [Chile]
*Neophyllaphis palliceps* Hille Ris Lambers, 1968	?P(c)	6	XX/X0	Blackman et al. 2003 [Chile]
*Neophyllaphis schlingeri* Hille Ris Lambers, 1968	P(c)	12	XX/X0	Blackman et al. 2003 [Chile]
*Neophyllaphis sensoriata* Hille Ris Lambers, 1968	P(c)	16	XX/X0	Blackman et al. 2003 [Chile]
*Neophyllaphis similis* Hille Ris Lambers, 1968	?P(c)	14	XX/X0	Blackman et al. 2003 [Chile]
*Neophyllaphis staryi* Quednau & Remaudière, 1994	?	14	XX/X0	Blackman et al. 2003 [Chile]
*Neophyllaphis valdiviana* Carrillo, 1980	?	6	XX/X0	Blackman et al. 2003 [Chile]
Neophyllaphis (Spicaphis) chilensis Essig, 1953	?	10	XX/X0	Blackman et al. 2003 [Chile]
Neophyllaphis (Spicaphis) essigi Hille Ris Lambers, 1968	?	12	XX/X0	Blackman et al. 2003 [Chile]
*Neuquenaphis* sp. 1	?	12	XX/X0	Blackman et al. 2003 [Chile]
*Neuquenaphis* sp. 2	?	16	XX/X0	Blackman et al. 2003 [Chile]
*Oestlundiella flava* (Davidson, 1912)	P(c)	8		Blackman 1980 [USA; Canada]
*Panaphis juglandis* (Goetze, 1778)	P(c)	22		Blackman and Eastop 1994 [?]
*Phyllaphis fagi* (Linnaeus, 1761)	P(c)	16		Blackman 1986 [Great Britain]
*Phyllaphis fagifoliae* Takahashi, 1919	P(c)	26/25	XX/X0	Shinji 1931 (as *Phyllaphis fagi* (Linnaeus, 1767) see Blackman 1986), Blackman 1986 [Japan] (based on n(♂) = 13 (Shinji 1931))
*Protopterocallis gigantea* Bissell, 1978	P(c)	10		Blackman 1980 [USA]
*Pterocallis alni* (De Geer, 1773)	P(c)	20		Blackman 1980 [USA]
*Pterocallis montana* (Higuchi, 1972)	?	16		Blackman 1986 [Japan]
Pterocallis (Recticallis) nigrostriata (Shinji, 1941)	P(c)	c. 26		Blackman and Eastop 1994 [?]
*Saltusaphis scirpus* Theobald, 1915	P(c)	10		Blackman and Eastop 2015 [?]
*Sarucallis kahawaluokalani* (Kirkaldy, 1907)	P(c)	6		Kurl 1978 (as *Neotherioaphis chhenafuli* Behura and Dash) [Meerut, Uttar Pradesh, India], Blackman 1980 [USA]
		8		Dutta and Khuda-Bukhsh 1980 (as *Tinocallis*) [Kalyani, West Bengal, India]
*Sinochaitophorus maoi* Takahashi, 1936	P(c)	10		Chen and Zhang 1985a [Beijing area, China] (cited after Blackman and Eastop 2015)
*Sensoriaphis nothofagi* Cottier, 1953	P(c)	10		Blackman 1980 [New Zealand]
*Shivaphis celti* Das, 1918	P(c), P(o)	6/5	XX/X0	Shinji 1927, 1931, 1941a, Blackman 1986 [Japan] (based on n(♂) = 3 (Shinji 1931))
		10		Chen and Zhang 1985a [Beijing area, China] (cited after Blackman and Eastop 2015), Blackman 1986 [Hong Kong]
Shivaphis (Sinishivaphis) hangzhouensis (G. Zhang & Zhong, 1982)	?	10		Blackman and Eastop 1994 [?]
*Stegophylla essigi* Hille Ris Lambers, 1966	P(c), P(o)	12		Blackman 1980 [USA]
*Stegophylla quercina* Quednau, 1966	P(c)	> 30		Blackman and Eastop 1994 (as *Stegophylla quercicola* (Monell, 1879) [?]
*Strenaphis elongata* (Baker, 1917)	P(c)	10		Blackman and Eastop 2015 [?]
*Subsaltusaphis aquatilis* (Ossiannilsson, 1959)	?	8		Blackman and Eastop 2006 [?]
*Subsaltusaphis flava* (Hille Ris Lambers, 1939)	P(c)	8		Blackman 1980 [Sweden]
*Subsaltusaphis lambersi kamijiensis* Sorin, 2005	P(c)	6		Blackman and Eastop 2015 [?] ("Blackman 1980, erroneously listed as *Subsaltusaphis saracola*")
*Subsaltusaphis ornata* (Theobald, 1927)	?	8		Gut 1976 [Holland]
*Subsaltusaphis picta* (Hille Ris Lambers, 1939)	P(c)	10		Blackman 1980 [Sweden]
*Subsaltusaphis virginica* (Baker, 1917)	P(c)	6		Blackman 1986 (as *Subsaltusaphis saracola* Higuchi, 1972) [Japan]
*Symydobius alniarius* (Matsumura, 1917)	P(c)	20		Blackman 1986 [Japan]
*Symydobius intermedius* Gillette and Palmer, 1930	P(c)	16		Blackman 1980 [USA]
*Symydobius oblongus* (von Heyden, 1837)	P(c)	14 (male), 15 (female)		Blackman 1988 [Great Britain; Sweden; Czechoslovakia]
		16		Gut 1976 [Holland]
Symydobius (Yezocallis) kabae (Matsumura, 1917)	P(c)	?26/25	XX/X0	Shinji 1931, 1941a, Blackman 1986 [Japan] (based on n(♂) = 13 (Shinji 1931))
*Takecallis arundicolens* (Clarke, 1903)	?P(c), ?P(o)	18		Blackman and Eastop 1984 [?], Blackman 1986 [Japan]
*Takecallis arundinariae* (Essig, 1917)	P(o), ?P(c)	18		Blackman 1980 [USA; Great Britain], Chen and Zhang 1985a [Beijing area, China] (cited after Blackman and Eastop 2015), Blackman 1986 [Great Britain], Khuda-Bukhsh and Kar 1990 [Shillong, Meghalaya, India]
*Takecallis taiwana* (Takahashi, 1926)	?P(c), ?P(o)	16		Blackman and Eastop 1984 [?]
*Tamalia coweni* (Cockerell, 1905)	P(c)	6/5	XX/X0	Morgan 1915 (as *Phyllaphis*), Ris 1942 [USA]
*Therioaphis natricis* Hille Ris Lambers & van den Bosch, 1964	?	16		Blackman and Eastop 2006 [?]
*Therioaphis ononidis* (Kaltenbach, 1846)	P(c)	16		Blackman and Eastop 2006 [?]
*Therioaphis tenera* (Aizenberg, 1956)	P(c)	6		Blackman and Eastop 2006 [?]
Therioaphis (Pterocallidium) trifolii trifolii (Monell, 1882)	P(c), P(o)	16		Blackman 1980 [USA]
Therioaphis (Pterocallidium) trifolii maculata (Buckton, 1899)	P(c), P(o)	16		Blackman 1980 [USA; Australia]
Therioaphis (Rhizoberlesia) riehmi (Börner, 1949)	P(c)	16		Robinson and Chen 1969a [Canada]
*Thripsaphis ballii pennsylvanica* Quednau, 2010	?	8		Blackman and Eastop 2006 [?]
*Tiliaphis coreana* Quednau, 1979	P(c)	38		Chen and Zhang 1985b (cited after Blackman and Eastop 2015)
*Therioaphis shinae* (Shinji, 1924)	P(c)	14/13	XX/X0	Shinji 1927, 1931, 1941a (as *Therioaphis*), Blackman 1986 [Japan] (based on n(♂) = 7 (Shinji 1931))
*Tinocallis ulmifolii* (Monell, 1979)	P(c)	8		MacDonald and Harper 1965 (as *Myzocallis*), Robinson and Chen 1969a [Canada]
*Tinocallis ulmiparvifoliae* Matsumura, 1919	P(c)	16		Blackman and Eastop 1994 [?]
*Tinocallis zelkowae* (Takahashi, 1919)	P(c)	12		Blackman 1986 (as *Tinocallis nirecola* (Shinji 1924) [Japan], Blackman and Eastop 1994 [?]
Tinocallis (Sappocallis) saltans (Nevsky, 1929)	P(c)	16		Chen and Zhang 1985b (cited after Blackman and Eastop 2015)
Tinocallis (Sappocallis) takachihoensis Higuchi, 1972	P(c)	16		Blackman and Eastop 2015 [?]
Tinocallis (Sappocallis) ulmicola (Matsumura, 1919)	?	16		Blackman 1986 (as *Sappocallis*) [Japan]
*Tinocalloides montanus* Basu, 1970 (1969)	P(c)	18		Kurl 1981 [Shillong, Meghayala, India]
Tuberculatus (Acanthocallis) quercicola (Matsumura, 1917)	?	14/13	XX/X0	Shinji 1927, 1931, 1941a, Blackman 1986 [Japan] (based on n(♂) = 7 (Shinji 1931))
		16		Blackman 1986 [Japan], Chen and Zhang 1985b (cited after Blackman and Eastop 2015)
Tuberculatus (Acanthotuberculatus) radisectuae G. Zhang, W. Zhang & Zhong, 1990	?	14		Chen and Zhang 1985b (cited after Blackman and Eastop 2015)
Tuberculatus (Nippocallis) kuricola (Matsumura, 1917)	P(c)	14/13	XX/X0	Shinji 1927, 1931 (as *Callipterus*) [Japan], Blackman 1986 (as *Myzocallis*) [Japan] (own data and based on n(♂) = 7 (Shinji 1931))
Tuberculatus (Orientuberculoides) capitatus (Essig et Kuwana, 1918)	P(c)	14		Blackman and Eastop 2015 [?]
Tuberculatus (Orientuberculoides) kashiwae (Matsumura, 1917)	P(c)	14/13	XX/X0	Shinji 1927, 1931 [Japan], Blackman 1986 [Japan] (own data and based on n(♂) = 7 (Shinji 1927, 1931))
Tuberculatus (Orientuberculoides) paranaracola hemitrichus Hille Ris Lambers, (1972) 1974	?	14		Blackman and Eastop 2015 [?]
Tuberculatus (Orientuberculoides) yokoyamai (Takahashi, 1923)	P(c)	14		Blackman 1986 [Japan]
Tuberculatus (Tuberculoides) annulatus (Hartig, 1841)	P(c)	14		Blackman 1980 (as *Tuberculoides*) [Great Britain; USA]
Tuberculatus (Tuberculoides) moerickei Hille Ris Lambers, (1972) 1974	?	14		Blackman and Eastop 2015 [?]
*Tuberculatus* sp.	?	14		Kar et al. 1990 [India]
*Yamatocallis takagii* (Takahashi, 1963)	?	c. 48		Blackman 1986 [Japan]
**Fam. Chaitophoridae**				
*Atheroides hirtellus* Haliday, 1839	P(c)	8		Blackman 1980 [Great Britain]
*Atheroides serrulatus* Haliday, 1839	P(c)	8		Blackman 1980 [Sweden]
*Chaitophorus capreae* (Mosley, 1841)	P(c)	30		Blackman 1980 [Great Britain]
*Chaitophorus dorocolus* Matsmura, 1919	P(c)	14		Shinji 1941a, Blackman 1986 [Japan] (based on n(♂) = 7 (Shinji 1941a)) (but see Blackman 1986 p. 77)
*Chaitophorus euphraticus* Hodjat, 1981	P(c), ?P(o)	22		Blackman and Eastop 2015 [?]
*Chaitophorus furcatus* Quednau ex Pintera, 1987	P(c)	16		Blackman and Eastop 1994 [?]
*Chaitophorus himalayensis* (Das, 1918)	?	18		Dutta and Gautam 1993 [Shimla, Himachal Pradesh, India]
*Chaitophorus indicus* A.K. Ghosh, M.R. Ghosh & D.N. Raychaudhuri, 1970	P(c)	18		Pal and Khuda-Bukhsh 1983 (as *Chaitophorus manaliensis* Chakrabarti, 1975) [Garhwal, Uttarakhand, India], Dutta and Gautam 1993 (as *Chaitophorus manaliensis* Chakrabarti, 1975) [Shimla, Himachal Pradesh, India]
*Chaitophorus inouyei* Hille Ris Lambers, 1976	?	26		Blackman and Eastop 2015 [?]
*Chaitophorus leucomelas* Koch, 1854	P(c)	4		Rubín de Celis and Ortiz 1993 [Lima, Peru]
		36		Blackman and Eastop 2015 [Israel]
		40		Blackman 1980 [Great Britain], Blackman and Eastop 2015 [Great Britain; South Africa]
*Chaitophorus?matsumurai* Hille Ris Lambers, 1960	?	14		Shinji 1927 (as *Chaitophorus salicicolus*), 1931 (as *Chaitophorus saliniger*), Blackman 1986 [Japan] (based on n(♂) = 7 (Shinji 1927, 1931, 1941))
*Chaitophorus neglectus* Hottes & Frison, 1931	P(c)	12		Robinson and Chen 1969a [Canada]
*Chaitophorus niger* Mordvilko, 1929	P(c)	30		Blackman and Eastop 2015 [?]
*Chaitophorus nigrae* Oestlund, 1886	P(c)	24		Blackman and Eastop 1994 [?]
*Chaitophorus nigritus* Hille Ris Lambers, 1966	P(c)	18		Dutta and Gautam 1993 [Shimla, Himachal Pradesh, India]
*Chaitophorus populeti* (Panzer, 1801)	P(c)	10		Pal and Khuda-Bukhsh 1982 [Srinagar, Jammu and Kashmir, India]
		12		Blackman and Eastop 1994 [Iran; China]
		14		Shinji 1941a (as *Chaitophorus populi*), Blackman 1986 [Japan] (based on n(♂) = 7 (Shinji 1941a)) (but see Blackman 1986, p. 77)
*Chaitophorus populialbae* (Boyer de Fonscolombe, 1841)	P(c)	28		Chen and Zhang 1985 (cited after Blackman and Eastop 2015)
		30		Blackman and Eastop 2015 [?]
*Chaitophorus populicola* Thomas, 1878	P(c)	18, 28, 32		Blackman and Eastop 1994 [?]
*Chaitophorus populifolii* (Essig, 1912)	P(c)	12		Robinson and Chen 1969a (also as *Chaitophorus populifolii neglectus* Hottes and Frison, 1931) [Canada]
*Chaitophorus saliapterus* Shinji, 1924	?	14/13	XX/X0	Shinji 1927, 1931, 1941a, Blackman 1986 [Japan] (based on n(♂) = 7 (Shinji 1931)) (Blackman and Eastop 2015: "Shinji’s record of 2n=14 (n=7) should probably be applied to another species of *Chaitophorus*")
		30		Blackman and Eastop 2015 [?}
*Chaitophorus salicti* (Schrank, 1801)	P(c)	28		Blackman 1980 [Sweden]
Chaitophorus prope salijaponicus niger (Mordvilko, 1929)	?	30		Kuznetsova and Shaposhnikov 1973 (Chaitophorus aff. niger Mordv.) [Georgia; Turkmenistan]
*Chaitophorus saliniger* Shinji 1924	P(c)	8		Shinji 1931, Blackman 1986 [Japan]
		14		Blackman 1986 [Japan] (based on n(♂) = 7 (Shinji 1931))
*Chaitophorus stevensis* Sanborn, 1904	?	14		Blackman and Eastop 2015 [?]
*Chaitophorus tremulae* Koch, 1854	P(c)	18		Blackman and Eastop 1994 [?]
*Chaitophorus truncatus* Hausmann, 1802	P(c)	30		Blackman and Eastop 2015 [?]
*Chaitophorus viminalis* Monell, 1879	P(c)	9, 10, 11		Morgan 1909b [USA]
		18		Robinson and Chen 1969a [Canada]
*Chaitophorus* sp. 1 (from *Populus euphratica*)	?	22		Blackman 1980 [Iran]
*Chaitophorus* sp. 2	?	26		Blackman 1986 [Japan]
*Periphyllus acericola* (Walker, 1848)	P(c)	18		Gut 1976 [Holland], Blackman and Eastop 1994 [?]
*Periphyllus aceris* (Linnaeus, 1761)	P(c)	16		Gut 1976 [Holland], Blackman and Eastop 1994 [?]
*Periphyllus californiensis* (Shinji, 1917)	P(c)	18		Blackman 1986 [Great Britain]
		20	XX/X0	Shinji 1927, 1931, 1941a (as *Periphyllus aceris*) [Japan] (but see Blackman 1986), Blackman 1986 [Japan] (based on n(♂) = 10 (Shinji 1931))
*Periphyllus coracinus* (Koch, 1854)	P(c)	18		Gut 1976 [Holland]
*Periphyllus hirticornis* (Walker, 1848)	P(c)	18		Gut 1976 [Holland], Blackman and Eastop 1994 [?]
*Periphyllus koelreuteriae* (Takahashi, 1919)	?	10		Chen and Zhang 1985a [Beijing area, China] (cited after Blackman and Eastop 2015)
		18		Blackman and Eastop 1994 [?]
		20/19	XX/X0	Shinji 1931, Blackman 1986 [Japan] (based on n(♂) = 10 (Shinji 1931))
		22		Shinji 1927, 1941a [Japan]
*Periphyllus kuwanaii* (Takahashi, 1919)	?	18		Blackman and Eastop 1994 [?]
*Periphyllus lyropictus* (Kessler, 1886)	P(c)	18		Gut 1976 [Holland]
*Periphyllus negundinis* (Thomas, 1878)	P(c)	20		Sun and Robinson 1966, Robinson and Chen 1969a [Canada]
*Periphyllus testudinaceus* (Ferni, 1852)	P(c)	18		Gut 1976 [Holland]
*Sipha flava* (Forbes, 1885)	P(c), P(o)	10		Mayo and Starks 1972 [USA]
*Sipha glyceriae* (Kaltenbach, 1843)	P(c)	10		Blackman and Eastop 2006 [?]
		12		Gut 1976 [Holland]
Sipha (Rungsia) elegans del Guercio, 1905	P(c)	6		Sun and Robinson 1966 (as *Sipha agropyrella* Hille Ris Lambers, 1939), Robinson and Chen 1969a (as *Sipha kurdjumovi* Mordvilko, 1921)[Canada]
Sipha (Rungsia) maydis Passerini, 1860	P(c), ?P(o)	12		Blackman and Eastop 2015 [?]
**Fam. Aphididae**				
*Abstrusomyzus phloxae* (Sampson, 1939)	P(o)	18		Blackman and Eastop 2006 [?]
*Acaudinum centaureae* (Koch, 1854)	P(c)	10		Kuznetsova 1968 (as *Acaudinum dolichosiphon* Mordvilko,1928) [St.Petersburg, Russia]
*Acyrthosiphon auriculae* Martin, 1981	P(c)	8		Martin 1981 [Great Britain]
*Acyrthosiphon bidenticola* Smith, 1960	?	8		Blackman and Eastop 2006 [?]
*Acyrthosiphon boreale* Hille Ris Lambers, 1952	P(c)	10		Blackman and Eastop 2015 [?]
*Acyrthosiphon caraganae caraganae* (Cholodkovsky, 1907(1908))	P(c)	10		Sun and Robinson 1966, Robinson and Chen 1969a [Canada]
*Acyrthosiphon caraganae occidentale* Hille Ris Lambers, 1947	P(c)	10		Blackman and Eastop 2015 [?]
*Acyrthosiphon ghanii* Eastor, 1971	P(c), P(o)	10		Kapoor and Gautam 1994 [Shimla, Himachal Pradesh, India] (Blackman and Eastop 2015: " …but aphid was possibly misidentified as host was *Medicago*"), Blackman and Eastop 2006 [?]
*Acyrthosiphon gossypii* Mordvilko, 1914	P(c), P(o)	6		Blackman 1980 [Iran], Gautam and Dhatwalia 2003 [Shimla, Himachal Pradesh, India]
*Acyrthosiphon ignotum* Mordvilko, 1914	?	14		Pal and Khuda-Bukhsh 1984 (as *Metopolophium*), Khuda-Bukhsh and Pal 1986b (as *Metopolophium*) [Jamunetri, Uttarakhand, India]
Acyrthosiphon sp. prope ignotum Mordvilko, 1914	?	10		Kuznetsova and Shaposhnikov 1973 [St. Petersburg, Russia]
*Acyrthosiphon kondoi* Shinji, 1938	P(c)	10		Blackman 1980 [USA], Blackman 1986 [Japan]
*Acyrthosiphon lactucae* (Passerini, 1860)	P(c)	16		Blackman and Eastop 1984 [?]
*Acyrthosiphon loti* (Theobald, 1913)	P(c)	10		Blackman 1980 [Great Britain]
*Acyrthosiphon macrosiphum* (Wilson, 1912)	P(c)	10		Blackman and Eastop 2015 [?]
*Acyrthosiphon malvae malvae* (Mosley, 1841)	P(c)	10		Blackman 1980 (as *Acyrthosiphon pelargonii* Kaltenbach, 1843) [Great Britain], Kurl and Chauhan 1987b [Barog, Himachal Pradesh, India]
		12		Kar et al. 1990 (as Metopolophium (Metopolophium) malvae (Mosley)) [India]
*Acyrthosiphon malvae poterii* Prior & Stroyan, 1964	P(c)	10		Blackman and Eastop 2015 [?]
*Acyrthosiphon malvae rogersii* (Theobald, 1913)	P(c)	10		Blackman and Eastop 2015 [?]
*Acyrthosiphon pisivorum* G. Zhang, 1980	?	14		Chen and Zhang 1985a [Beijing area, China] (cited after Blackman and Eastop 2015)
*Acyrthosiphon pisum* (Harris, 1776)	P(c)	8/7	XX/X0	Pagliai 1965, Manicardi, Bizzaro et al. 1991, Bizzaro et al. 2000 [Italia]
		8		Suomalainen 1933 (as *Macrosiphum pisi* (Kaltenbach, 1843)) [Finland], Colling 1955 [Great Britain], Sun and Robinson 1966, Harper and MacDonald 1968, Robinson and Chen 1969a [Canada], Kuznetsova and Shaposhnikov 1973 (as *Dactinotus basalis* Walker, 1948) [St. Petersburg, Russia], Kuznetsova 1974 (as *Dactinotus basalis* Walk.) [?], Blackman 1986 [Japan], Khuda-Bukhsh and Kar 1990 [Kalimpong, West Bengal, India], Kar et al. 1990 [India], Blackman and Spence 1996 [Great Britain], Gautam and Dhatwalia 2003 [Shimla, Himachal Pradesh, India]
*Acyrthosiphon primulae* (Theobald, 1913)	?	16		Blackman and Eastop 2015 [?]
*Acyrthosiphon rubi* Narzikulov, 1957	P(c)	10		Pal and Khuda-Bukhsh 1982, Khuda-Bukhsh and Pal 1986b (as *Metopolophium sonchifoliae* Raychaudhuri, Ghosh & Das, 1980) [Srinagar, Jammu and Kashmir, India]
		12		Kurl and Chauhan 1986c, Kurl and Chauhan 1987a (as *Metapolophium*) [Barog, Himachal Pradesh, India], Dutta and Gautam 1993 [Shimla, Himachal Pradesh, India]
*Acyrthosiphon scariolae* Nevsky, 1929	?	18		Blackman and Eastop 1984 [?]
*Akkaia polygoni* Takahashi, 1919	P(c)	12		Shinji 1927, 1931 [Japan] (Blackman 1986 supposed that "Shinji’s immature males were of another species of *Akkaia*")
		24		Blackman 1986 [Japan]
*Akkaia* sp.	?	12/11	XX/X0	Shinji 1931 [Japan] (see comments of Blackman 1986)
*Aleurosiphon smilacifoliae* (Takahashi, 1921)	P(c)	8		Blackman 1986 [Japan]
*Amphicercidus japonicus* (Hori, 1927)	P(c)	8		Chen and Zhang 1985b (cited after Blackman and Eastop 2015)
*Acyrthosiphon lonicerae* Maity & Chakrabarti, 1982	?	18		Khuda-Bukhsh and Pal 1983a [Garhwal, Uttarakhand, India] (Blackman and Eastop 2015: «this was probably an error»)
*Acyrthosiphon tuberculatus* David, Narayanan & Rajasingh, 1970 (1971)	?	6		Chauhan and Kurl 1990 [Dachigam, Jammu and Kashmir, India]
		12		Pal and Khuda-Bukhsh 1984 [Jamunetri, Uttarakhand, India]
*Amphicercidus* sp.		8?		Khuda-Bukhsh 1980 [Garhwal, Uttarakhand, India] (Blackman and Eastop 2015: *Amphicercidus lonicerae* Maity and Chakrabarti)
*Amphorophora agathonica* Hottes, 1950	P(c)	14		Robinson and Chen 1969a [Canada]
*Acyrthosiphon ampullata ampullata* Buckton, 1876	P(c)	12		Blackman 1980, 2010 [Great Britain], Blackman 1986 [Japan]
*Acyrthosiphon ampullata bengalensis* Hille Ris Lambers & Basu, 1966	?	12		Kurl and Chauhan 1986a [Kandaghat, Himachal Pradesh, India], Kurl and Chauhan 1987a [Manali, Himachal Pradesh, India]
*Acyrthosiphon ampullata laingi* (Mason, 1925)	?	12		Sun and Robinson 1966 (as *Acyrthosiphon laingi* (Mason, 1925)), Robinson and Chen 1969a (as *Acyrthosiphon laingi* (Mason, 1925)) [Canada]
*Acyrthosiphon amurensis* (Mordvilko, 1919)	?	14		Blackman 1986 [Japan]
*Acyrthosiphon forbesi* Richards, 1959	?	12		Blackman and Eastop 2015 [?]
*Acyrthosiphon gei* (Börner, 1939)	P(c)	12		Blackman 1980 [Great Britain]
*Acyrthosiphon idaei* (Börner, 1939)	P(c)	18		Blackman et al. 1977 [Europe], Blackman 1980 [Great Britain; Germany]
*Acyrthosiphon pacifica* Hill, 1968	P(o)	18		Blackman 1980 [USA]
*Acyrthosiphon parviflori* Hill, 1958	?	12		Blackman 1980 [USA; Canada]
*Acyrthosiphon rossi* Hottes & Frison, 1931	P(c)	46		Blackman and Eastop 2006 [?]
*Acyrthosiphon rubi* (Kaltenbach, 1843)	P(c), P(o)	20, 21		Blackman et al. 1977 [Europe], Blackman 1980 [Great Britain]
*Acyrthosiphon rubitoxica* Knowlton, 1954	?	30		Blackman 1980 [USA; Canada]
*Acyrthosiphon sensoriata* Mason, 1923	?	72		Blackman 1980 [USA; Canada]
*Acyrthosiphon stachyophila* Hille Ris Lambers, 1966	?	12		Blackman 1980 [USA]
*Acyrthosiphon stolonis* Robinson, 1974	P(c)	48		Blackman 1980 [Canada]
*Acyrthosiphon tigwatensa* Hottes, 1933	?	40		Blackman and Eastop 2006 [?]
*Acyrthosiphon tuberculata* Brown & Blackman, 1985	P(c)	4	XX/X0	Blackman 1985, Blackman and Hales 1986, Blackman and Spence 1996, Spence and Blackman 1998 [Great Britain]
*Amphorophora* sp.	?	10		Blackman and Eastop 2006, Blackman 2010 [populations on *Athyrium felix-femina* in Netherlands and Great Britain]
*Anuraphis catonii* Hille Ris Lambers, 1935	P(c)	22		Kuznetsova 1968 [Crimea, Ukraine]
		26		Kuznetsova 1975 [Crimea, Ukraine]
*Anuraphis farfarae* (Koch, 1854)	P(c)	12		Kuznetsova 1968, 1975 [St. Petersburg, Russia], Kuznetsova 1974 [?]
*Anuraphis farfarae dianae* Shaposhnikov, 1974	P(o)	12		Kuznetsova and Shaposhnikov 1973 [Georgia], Kuznetsova 1974 [?]
*Anuraphis pyrilaseri* Shaposhnikov, 1950	P(c), P(o)	12		Kuznetsova 1968, 1975 [Crimea, Ukraine]
*Anuraphis subterranea* (Walker, 1852)	P(c), P(o)	22		Kuznetsova 1968 [St. Petersburg, Russia]
		26		Kuznetsova 1975 [St. Petersburg, Russia]
*Anuromyzus cotoneasteris* (Shaposhnikov, 1959)	P(c)	12		Kuznetsova 1968 (as Dysaphis (Anuromyzus)) [Georgia]
*Aphidura pannonica* Szelegiewicz, 1967	?	12		Blackman 1980 [Greece]
*Aphis acaenovinae* Eastop, 1961	?	8		Blackman and Eastop 2006 [Australia] ("D.F. Hales, pers. comm.")
*Aphis achyranthi* Theobald, 1929	?	7		Kurl and Chauhan 1987a [Solan, Himachal Pradesh,, India]
		8		Kurl and Chauhan 1986b [Solan, Himachal Pradesh, India]
*Aphis affinis* Del Guercio, 1911	P(c)	8		Pal and Khuda-Bukhsh 1982, Khuda-Bukhsh and Pal 1985 [Srinagar, Jammu and Kashmir, India], Gautam and Sharma 1990 [Himachal Pradesh, India], Dutta and Gautam 1993, Gautam and Dhatwalia 2003 [Shimla, Himachal Pradesh, India]
*Aphis amaranthi* Holman, 1974	?	8		Blackman 1980 [USA]
*Aphis armata* Hausmann, 1802	P(c)	8		Gut 1976 [Holland]
*Aphis asclepiadis* Fitch, 1851	?	8		Stevens 1906, 1909 [USA], Robinson and Chen 1969a [Canada], Chen and Zhang 1985a [Beijing area, China] (cited after Blackman and Eastop 2015)
*Aphis brunnea* Ferrari, 1872	?	8		Blackman and Eastop 2015 [?]
*Aphis carduella* Walsh, 1863	P(c)	8		Sun and Robinson 1966 (as *Aphis helianthi* Monell, 1879), Robinson and Chen 1969a (as *Aphis helianthi* Monell, 1879 and *Aphis kurosawella* Davis, 1919) [Canada]
*Aphis celastrii* Matsumura, 1917	P(c)	8		Blackman 1986 (as *Aphis citricola celastrii* Matsumura, 1917) [Japan]
*Aphis chloris* Koch, 1854	P(c)	8		Blackman and Eastop 2015 [?]
*Aphis clematidis* Koch, 1854	P(c)	8		Khuda-Bukhsh and Pal 1985 [Garhwal, Uttarakhand, India]
*Aphis clerodendri* Matsumura, 1917	?	8		Blackman and Eastop 2015 [?]
Aphis near clerodendri Matsumura, 1917	?	8		Chen and Zhang 1985a [Beijing area, China] (cited after Blackman and Eastop 2015)
*Aphis clinopodii* Passerini, 1861	P(c)	8		Blackman and Eastop 2015 [?]
		7,8		Kurl 1986 [Meghalaya, India]
*Aphis craccivora* Koch, 1854	P(o), P(c)	8		Kurl 1978 [Jodhpur, Rajasthan; Modinagar, Uttar Pradesh, India], Kulkarni and Kacker 1979 [Kolkata, West Bengal, India], Blackman 1980 [USA; Iran], Chen and Zhang 1985a, c (also as *Aphis robiniae* Machiati) [Beijing area, China] (Chen and Zhang 1985a cited after Blackman and Eastop 2015), Kurl and Chauhan 1986b [Himachal Pradesh, India], Kuznetzova and Sapunov 1985, 1987 [Russia], Kar and Khuda-Bukhsh 1989 [Kalimpong, West Bengal, India], Kar et al. 1990 [India], Sen and Khuda-Bukhsh 1992 [West Bengal, India], Dutta and Gautam 1993 [Kangra, Himachal Pradesh, India], Kapoor and Gautam 1994 [Himachal Pradesh, India], Bakhtadze et al. 2010 [Georgia]
		9		Blackman 1980 [Iran] (from *Lupinus*)
*Aphis crepidis* (Börner, 1940)	P(c)	8		Blackman and Eastop 2015 [?]
*Aphis cytisorum cytisorum* Hartig, 1841	P(c)	8		Blackman 1980 [USA], Chen and Zhang 1985a (as *sophoricola* Zhang) [Beijing area, China] (cited after Blackman and Eastop 2015)
*Aphis cytisorum sarothamni* Franssen, 1928	P(c)	8		Blackman and Eastop 2015 [?]
*Aphis epilobii* Kaltenbach, 1843	P(c)	8		Gut 1976 [Holland]
*Aphis eugeniae* van der Goot, 1917	?	8		Blackman 1980 [Philippines; Australia]
*Aphis fabae fabae* Scopoli, 1763	P(c)	8		Colling 1955 [Great Britain], Orlando 1965 [Italy], Kurl and Chauhan 1986b, 1987a [Kangra, Himachal Pradesh, India], Kuznetsova and Gandrabur 1991 [St.Petersburg, Russia], Dutta and Gautam 1993 [Solan, Himachal Pradesh, India], Kapoor and Gautam 1994 [Himachal Pradesh, India], Blackman and Spence 1996 [Great Britain], Rivi et al. 2009 [Italy] Jangra et al. 2014 [Jammu and Kashmir, India]
		8, 9		Panigrahy and Patnaik 1991 (as *Aphis citricola*) [Chatrapur, Odisha, India]
		8 (structural heterozygosity)		Blackman 1980 [anholicyclic population in California, USA]
*Aphis fabae evonymi* Fabricius, 1775	P(c)	8		Blackman and Eastop 2015 [?]
*Aphis fabae mordvilkoi* Börner & Janich 1922	P(c)	8		Blackman 1980 [Great Britain], Chen and Zhang 1985a [Beijing area, China] (cited after Blackman and Eastop 2015) Blackman and Eastop 2015 [?]
*Aphis farinosa* J.F. Gmelin, 1790	P(c)	6/5		Baehr 1908, 1912 (as *Aphis saliceti*) [Germany], Morgan 1909b (as *Aphis salicola* Gillette & Baker, 1895) [USA], Shinji 1941a (as *Aphis saliceti*) [Japan]
		6		Stevens 1906 [USA], Baehr 1909 (as *Aphis saliceti*) [Germany], Kuznetsova and Shaposhnikov 1973 [?], Chen and Zhang 1985a [Beijing area, China] (cited after Blackman and Eastop 2015)
		6	XX/X0	Kuznetsova and Gandrabur 1991 [St.Petersburg, Russia]
*Aphis forbesi* Weed, 1889	P(c)	8		Blackman and Eastop 1984 [?]
*Aphis frangulae* Kaltenbach, 1845	P(c), ?P(o)	8		Blackman and Eastop 2015 [?]
*Aphis fukii* Shinji, 1922	?	8		Blackman 1986 [Japan]
*Aphis genistae* Scopoli, 1763	P(c)	8		Gut 1976 [Holland]
*Aphis gossypii* Glover, 1877	P(o), P(c)	8		Stevens 1906, 1909 [USA], Shinji 1927 [Japan], Robinson and Chen 1969a [Canada], Kurl 1978 [Jodhpur, Rajasthan; Modinagar and Meerut, Uttar Pradesh, India], Kulkarni and Kacker 1979 [Baruipur, West Bengal, India], Chattopadhyay and Raychaudhuri 1980 [Kolkata, West Bengal, India], Khuda-Bukhsh and Datta 1981a, b [India], Khuda-Bukhsh and Pal 1985 [Srinagar, Jammu and Kashmir, India], Chen and Zhang 1985a [Beijing area, China] (cited after Blackman and Eastop 2015), Blackman 1986 [Japan] (their own data and based on n(♂) = 4 (Shinji 1927)), Kurl and Chauhan 1986b [Himachal Pradesh, India], Khuda-Bukhsh and Kar 1989b, Kar et al. 1990 [India], Gautam and Sharma 1990 [Himachal Pradesh, India], Kar and Khuda-Bukhsh 1991a [Meghalaya, West Bengal, India], Dutta and Gautam 1993 [Shimla, Himachal Pradesh, India], Gautam and Dhatwalia 2003 [Solan, Himachal Pradesh, India], Samkaria et al. 2010 [Palampur, Himachal Pradesh, India], Reeta Devi and Gautam 2012 [Kullu region, Himachal Pradesh, India]
		10		Shinji 1941a [Japan]
*Aphis healyi* Cottier, 1953	P(c)	8		Blackman and Eastop 2006 [New Zealand]
*Aphis hederae hederae* Kaltenbach, 1843	P(c)	8		Bakhtadze et al. 2010 [Georgia]
*Aphis hederae pseudohederae* Theobald, 1927	?	8		Blackman 1980 (as Aphis hederae form pseudohederae Theobald) [USA]
*Aphis horii* Takahashi, 1923	?	8		Chen and Zhang 1985b (cited after Blackman and Eastop 2015)
*Aphis hyperici* Monell, 1879	P(c)	8		Blackman and Eastop 2015 [?]
*Aphis ichigo* Shinji, 1922	?	8		Blackman 1986 [Japan]
*Aphis idaei* van der Goot, 1912	P(c)	8		Blackman 1980 [Great Britain]
*Aphis ilicis* Kaltenbach, 1843	?P(c)	8		Blackman 1980 [Great Britain]
*Aphis kurosawai* Takahashi, 1921	?P(c)	8		Blackman 1986 [Japan], Kurl and Chauhan 1987a, 1987b [Solan, Himachal Pradesh, India]
Aphis near kurosawai Takahashi, 1921	?	8		Chen and Zhang 1985a [Beijing area, China] (cited after Blackman and Eastop 2015)
*Aphis lambersi* (Börner, 1940)	P(c)	8		Blackman and Eastop 2015 [?]
*Aphis longisetosa* Basu, 1969(1970)	?	6		Kurl and Chauhan 1987a, b (as *Aphis ruborum longisetosus* Basu) [Solan, Himachal Pradesh, India], Khuda-Bukhsh and Kar 1990 (as *Aphis ruborum longisetosus* Basu) [Shillong, Meghalaya, India]
		8		Khuda-Bukhsh 1982 (as *Aphis ruborum longisetosus*) [Mussoorie, Uttarakhand, India], Khuda-Bukhsh and Pal 1985 (as *Aphis ruborum longisetosus*) [Srinagar, Jammu and Kashmir, India]
*Aphis longituba* Hille Ris Lambers, 1966	?	8		Kar et al. 1990 (as *Aphis clematidis simlaensis* Kumar & Burkhardt) [India], Dutta and Gautam 1993 (as *Aphis clematidis simlaensis* Kumar & Burkhardt) [Shimla, Himachal Pradesh, India]
*Aphis loti* Kaltenbach, 1862	P(c)	8		Blackman 1980 [Great Britain]
*Aphis maculatae* Oestlund, 1887	P(c)	8		Robinson and Chen 1969a [Canada]
*Aphis nasturtii* Kaltenbach, 1843	P(c)	8		Dionne and Spicer 1957 (as *Aphis abbreviata* Patch), Sun and Robinson 1966, Robinson and Chen 1969a [Canada], Kurl and Chauhan 1986b [Himachal Pradesh, India], Kar and Khuda-Bukhsh 1989 [Kalimpong, West Bengal, India], Dutta and Gautam 1993 [Shimla, Himachal Pradesh, India], Kapoor and Gautam 1994 [Himachal Pradesh, India], Gautam and Dhatwalia 2003 [Shimla, Himachal Pradesh, India], Samkaria et al. 2010 [Yol, Himachal Pradesh, India]
*Aphis neogillettei* Palmer, 1938	P(c)	8		Sun and Robinson 1966, Robinson and Chen 1969a [Canada]
*Aphis nerii* Boyer de Fonscolombe, 1841	P(o), P(c)	8		Kurl 1978 [Jodhpur, Rajasthan; Modinagar, Uttar Pradesh, India], Kulkarni and Kacker 1980 [India], Blackman 1980 [Great Britain], Chattopadhyay and Raychaudhuri 1980 [Kolkata, West Bengal, India], Khuda-Bukhsh and Datta 1981b [India], Khuda-Bukhsh and Pal 1985 [Garhwal, Uttarakhand, India], Kapoor and Gautam 1994 [Nahan, Himachal Pradesh, India]
*Aphis newtoni* Theobald, 1927	P(c)	8		Blackman 1980 [Great Britain]
*Aphis odinae* (van der Goot, 1917)	P(o), P(c)	8		Kurl 1980a (as *Toxoptera*) [Assam, Meghalaya, India], Pal and Khuda-Bukhsh 1980 (as *Toxoptera*) [Uttarakhand, India], Khuda-Bukhsh and Pal 1984a (as *Toxoptera*) [Triyuginarayan, Uttarakhand, India], Chen and Zhang 1985a (as *Toxoptera*) [Beijing area, China] (cited after Blackman and Eastop 2015), Blackman 1986 (as *Toxoptera*) [Japan], Kar and Khuda-Bukhsh 1989 (as *Toxoptera*) [Kalimpong, West Bengal, India], Kar et al. 1990 (as *Toxoptera*) [India]
		10		Shinji 1941a [Japan]
*Aphis oestlundi* Gillette, 1927	P(c)	8		Blackman and Eastop 2015 [?]
*Aphis paraverbasci* Chakrabarti (1976) 1977	?	8		Kurl and Chauhan 1986b, 1987a [Solan, Himachal Pradesh, India]
*Aphis parietariae* Theobald, 1922	P(c)	8		Blackman and Eastop 2015 [?]
*Aphis platylobii* Carver & White, 1970	?	8		Blackman and Eastop 2006 [New South Wales, Australia]
*Aphis polygonata* (Nevsky, 1929)	P(c)	8		Blackman and Eastop 2006 [?]
*Aphis pomi* De Geer, 1773	P(c)	8		Robinson and Chen 1969a [Canada], Kuznetsova and Shaposhnikov 1973 [Leningrad Prov., Russia], Gautam and Kumari 2003 [Shimla, Himachal Pradesh, India]
		7,8,9		Criniti et al. 2005 [Italy]
*Aphis punicae* Passerini, 1863	P(c), P(o)	8		Blackman and Eastop 1984 [?], Panigrahy and Patnaik 1987 [Chatrapur, Odisha, India], Dutta and Gautam 1993 [Shimla, Himachal Pradesh, India], Gautam and Dhatwalia 2003 [Solan, Himachal Pradesh, India]
*Aphis rhamnifila* David, Narayanan & Rajasingh, 1971	?	8		Khuda-Bukhsh 1982 [Mussoorie, India]
*Aphis rubicola* Oestlund, 1887	P(c)	8		Sun and Robinson 1966, Robinson and Chen 1969a [Canada]
*Aphis ruborum* (Börner & Schilder, 1931)	P(c)	8		Blackman 1980 [Great Britain], Bakhtadze et al. 2010 [Georgia]
*Aphis rumicis* Linnaeus, 1758	P(c)	8		Colling 1955 [Great Britain]
*Aphis salicariae* Koch, 1855	P(c)	8		Sun and Robinson 1966 (as *Aphis corniella* Hille Ris Lambers, 1935), Robinson and Chen 1969a (as *Aphis corniella* Hille Ris Lambers, 1935) [Canada]
*Aphis sambuci* Linnaeus, 1758	P(c), P(o)	8		Gut 1976 [Holland], Blackman 1986 [Europe; Japan], Manicardi et al. 1998 [Italy]
*Aphis sambuci* group	?	10		Shinji 1941a (as *Aphis sambuci* Linnaeus) [Japan]
		12		Shinji 1927, 1931 (as *Aphis sambuci* Linnaeus), Blackman 1986 [Japan] (based on n(♂) = 6 (Shinji 1927, 1931))
*Aphis sedi* Kaltenbach, 1843	P(c)	8		Blackman and Eastop 2015 [?]
*Aphis solanella* Theobald, 1914	P(c), ?P(o)	7		Blackman 1980 [Iran] (from *Solanum*)
		8		Blackman 1980 [Great Britain], Khuda-Bukhsh and Pal 1985 (as *Aphis fabae solanella* Theobald, 1914) [Garhwal, Uttarakhand, India], Kar and Khuda-Bukhsh 1989 (as *Aphis fabae solanella* Theobald) [Kalimpong, West Bengal, India], Gautam and Sharma 1990 [Himachal Pradesh, India], Kar et al. 1990 (as *Aphis fabae solanella* Theobald) [India], Dutta and Gautam 1993 (as *Aphis fabae solanella* Theobald, 1914) [Shimla, Himachal Pradesh, India], Gautam and Dhatwalia 2003 (as *Aphis fabae solanella*) [Shimla, Himachal Pradesh, India]
*Aphis spiraecola* Patch, 1914	P(c), P(o)	8		Sun and Robinson 1966, Robinson and Chen 1969a [Canada], Kurl 1978 [Jodhpur, Rajasthan, India], Kulkarni and Kacker 1981a [Solan, Himachal Pradesh, India], Khuda-Bukhsh 1982 [Mussoorie, India], Khuda-Bukhsh and Pal 1985 [Garhwal, Uttarakhand, India], Kurl and Chauhan 1986a, b (as *Aphis citricola* van der Goot) [Himachal Pradesh, India], Panigrahy and Patnaik 1987 (as *Aphis citricola* van der Goot) [Chatrapur, Odisha, India], Kar and Khuda-Bukhsh 1989 (as *Aphis citricola* van der Goot) [Shillong, Meghalaya, India], Sen and Khuda-Bukhsh 1992 [West Bengal, India], Kapoor and Gautam 1994 [Nahan, Himachal Pradesh, India], Gautam and Dhatwalia 2003 (as *Aphis citricola* van der Goot) [Solan, Himachal Pradesh, India]
		10		Chen and Zhang 1985a (as *Aphis citricola* van der Goot) [Beijing area, China] (cited after Blackman and Eastop 2015)
*Aphis spiraephaga* F.P. Müller, 1961	P(c)	8		Blackman and Eastop 2015 [?]
*Aphis spiraephila* Patch, 1914	P(c)	8		Robinson and Chen 1969a [Canada]
*Aphis subnitida* (Börner, 1940)	?	8		Blackman and Eastop 2015 [?]
*Aphis taraxacicola* (Börner, 1940)	P(c)	8		Blackman 1980 [Great Britain]
*Aphis thaspii* Oestlund, 1887	?	8		Robinson and Chen 1969a [Canada]
*Aphis triglochinis* Theobald, 1926	P(c)	8		Blackman and Eastop 1984 [?], Turčinavičienė et al. 1997 [Lithuania]
*Aphis ulicis* Walker, 1870	P(c)	8		Blackman 1980 [Great Britain]
*Aphis umbrella* (Börner, 1950)	P(c), ?P(o)	6		Blackman and Eastop 2006 [Iran]
		7		Blackman 1980 [Iran]
		8		Blackman and Eastop 2006 [Israel; Cyprus; Italy; Great Britain]
*Aphis verbasci* Schrank, 1801	P(c)	8		Khuda-Bukhsh and Pal 1985 [Kalyaani, West Bengal, India]
*Aphis viburni* Scopoli, 1763	P(c)	8		Colling 1955 [Great Britain]
*Aphis violae* Shouteden, 1900	P(c)	8		Blackman and Eastop 2015 [?]
Aphis (Bursaphis) epilobiaria Theobald, 1927	P(c)	8		Blackman 1980 [Great Britain]
Aphis (Bursaphis) fluvialis Martin, 1982	?P(o)	9		Blackman and Eastop 2015 [?]
Aphis (Bursaphis) grossulariae Kaltenbach, 1843	P(c)	8		Turčinavičienė et al. 1997 [Lithuania]
Aphis (Bursaphis) neomexicana (Cockerell & Cockerell, 1901)	?	8		Robinson and Chen 1969a [Canada]
Aphis (Bursaphis) oenotherae Oestlund, 1887	?P(c), P(o)	8		Blackman and Eastop 2015 [?]
		10/9		Stevens 1905a, b, 1906, 1910 [USA]
Aphis (Bursaphis) schneideri (Börner, 1940)	P(c)	8		Turčinavičienė et al. 1997 [Lithuania]
Aphis (Bursaphis varians Patch, 1914	P(c)	8		Sun and Robinson 1966, Robinson and Chen 1969a [Canada]
Aphis (Toxoptera) aurantii Boyer de Fonscolombe	P(o), ?P(c)	8		Pagliai 1961 [Italy], Kurl 1980a [Assam, Meghalaya, India], Kar et al. 1990 [India], Kar and Khuda-Bukhsh 1991a [Jammu and Kashmir, Meghalaya, West Bengal, India], Gautam and Dhatwalia 2003 [Shimla, Himachal Pradesh, India], Samkaria et al. 2010 [Palampur, Himachal Pradesh, India]
		8, 9		Panigrahy and Patnaik 1991 [Chatrapur, Odisha, India]
Aphis (Toxoptera) citricidus (Kirkaldy)	P(o), P(c)	7, 8		Kurl 1980a [Assam, Meghalaya, India], Kurl 1986 [Meghalaya, India], Kar and Khuda-Bukhsh 1989 [Kalimpong, West Bengal, India], Kar et al. 1990 [India]
Aphis (Toxopterina) vandergooti (Börner, 1939)	P(c)	8		Kuznetsova and Shaposhnikov 1973 (as *Chomaphis*) [Leningrad Prov., Russia], Blackman 1980 (as *Toxopterina*) [Great Britain]
*Aphis* sp. 1	?	8		Robinson and Chen 1969a [Canada]
*Aphis* sp. 2	?	8		Robinson and Chen 1969a [Canada]
*Aphis* sp. 3	?	8		Kuznetsova and Shaposhnikov 1973 [Crimea, Ukraine]
*Aphis* sp. 4	?	10		Khuda-Bukhsh and Pal 1985 [Srinagar, Jammu and Kashmir, India]
*Aphis* sp. 5	?	8		Chen and Zhang 1985a [Beijing area, China] (cited after Blackman and Eastop 2015)
*Aphis* sp. 6	?	8		Chen and Zhang 1985a [Beijing area, China] (cited after Blackman and Eastop 2015)
*Aphis* sp. 7	?	10		Khuda-Bukhsh and Kar 1990 [Shillong, Meghalaya, India]
*Aphis* sp. 8	?	8		Dutta and Gautam 1993 [Shimla, Himachal Pradesh, India]
*Aphis* sp. 9	?	8		Dutta and Gautam 1993 [Shimla, Himachal Pradesh, India]
*Aphis* sp. 10 (*Aphis gossypii* complex)	?	8		Dutta and Gautam 1993 [Solan, Himachal Pradesh, India], Kapoor and Gautam 1994 [Nahan, Himachal Pradesh, India]
*Aphthargelia symphoricarpi* (Thomas, 1878)	P(c)	14		Sun and Robinson 1966, Robinson and Chen 1969a [Canada]
*Aspidaphis adjuvans* (Walker, 1848)	P(c)	12		Sun and Robinson 1966, Robinson and Chen 1969a [Canada]
		14,16		Blackman and Eastop 2006 [Cyprus; Israel; Iran]
Aspidophorodon (Eoessigia) longicauda (Richards, 1963)	?	20		Blackman and Eastop 2015 [?]
*Atarsos grindeliae* Gillette, 1911	P(c)	12		Robinson and Chen 1969a [Canada]
*Aulacophoroides hoffmanni* (Takahashi, 1937)	P(c)	14		Blackman and Eastop 2006 [?China]
*Aulacorthum cercidiphylli* (Matsmura, 1918)	?P(c)	12		Blackman 1986 [Japan]
*Aulacorthum cirsicola* (Takahashi, 1923)	P(c)	10		Blackman 1986 [Japan]
*Aulacorthum dorsatum* Richards, 1967	P(c)	12		Blackman and Eastop 2006 [?Western North America]
*Aulacorthum flavum* F.P. Müller, 1958	P(c)	12		Blackman and Eastop 2006 [?]
*Aulacorthum ibotum* (Essig & Kuwana, 1918)	?	14/13	XX/X0	Shinji 1927 (as *Macrosiphum ligustrumae*), 1931 (as *Macrosiphum*), Blackman 1986 [Japan] (based on n(♂) = 7 (Shinji 1927))
*Aulacorthum linderae* (Shinji, 1922)	?P(c)	12		Shinji 1941b (as *Myzus*), Blackman 1986 [Japan] (based on n(♂) = 6 (Shinji 1941b))
*Aulacorthum magnoliae* (Essig & Kuwana, 1918)	P(c)	12/11	XX/X0	Shinji 1931, 1941a (as *Amphorophora*) [Japan]
*Aulacorthum muradachi* (Shinji, 1928)	?P(c)	10		Blackman and Eastop 2015 [?]
*Aulacorthum myriopteroni* (G. Zhang, 1980)	?	10		Chen and Zhang 1985b (cited after Blackman and Eastop 2015)
*Aulacorthum palustre* Hille Ris Lambers, 1947	?P(c), ?P(o)	34		Blackman and Eastop 2006 [?]
*Aulacorthum phytolaccae* Miyazaki, 1968	?	10		Blackman and Eastop 2006 [Japan]
*Aulacorthum sensoriatum* (David, Narayanan & Rajasingh, 1971)	?	18		Dutta and Gautam 1993 [Shimla, Himachal Pradesh, India]
*Aulacorthum smilacis* Takahashi, 1965	?	10		Blackman 1986 [Japan]
*Aulacorthum solani* (Kaltenbach, 1843)	P(c), P(o)	9, 10, 11		Blackman 1980 [Great Britain; California, USA]
		10		Dionne and Spicer 1957 [Canada], Pagliai 1966 [Italy], Kuznetsova and Shaposhnikov 1973 [St. Petersburg, Russia; Crimea, Ukraine], Kulkarni and Kacker 1980 [India], Pal and Khuda-Bukhsh 1980 [Triyuginarayan, Uttarakhand, India], Blackman 1986 [Japan], Khuda-Bukhsh and Pal 1986b [Triyuginarayan, Uttarakhand, India], Kapoor and Gautam 1994 [Nahan, Himachal Pradesh, India], Samkaria et al. 2010 [Shimla, Himachal Pradesh, India]
*Aulacorthum speyeri* Börner, 1939	P(c)	10		Blackman 1980 [Iran]
*Aulacorthum spinacaudatum* (Kumar & Burchardt, 1971)	?P(c)	12		Khuda-Bukhsh and Basu 1987 (as *Aulacorthum magnoliae*) (cited after Blackman and Eastop 2015)
*Aulacorthum* sp. 1	?	12		Khuda-Bukhsh and Kar 1990 [Kalimpong, West Bengal, India]
*Aulacorthum* sp. 2	?	12		Samkaria et al. 2010 [Yol, Himachal Pradesh, India]
*Brachycaudus helichrysi* (Kaltenbach, 1843)	P(c), P(o)	10, 11, 12, 13		Kurl 1986 [Meghalaya, India]
		12		Kuznetsova 1968 [Georgia], Kurl 1978 [Delhi, India], Pal and Khuda-Bukhsh 1980 [Gourikund, Uttarakhand, India], Kulkarni and Kacker 1981a [Dadhau, Himachal Pradesh, India], Raychaudhuri and Das 1987 [India], Gautam and Sharma 1990 [Himachal Pradesh, India], Dutta and Gautam 1993 [Shimla, Himachal Pradesh, India]
*Brachycaudus spiraeae* Börner, 1932	P(c)	12		Gut 1976 [Holland]
Brachycaudus (Acaudus) klugkisti (Börner, 1942)	P(c)	10		Blackman and Eastop 2015 [?]
Brachycaudus (Acaudus) lychnidis (Linnaeus, 1758)	P(c)	12		Kuznetsova 1968 [St.Petersburg, Russia]
Brachycaudus (Acaudus) populi (del Guercio, 1911)	P(c)	12		Blackman and Eastop 2015 [?]
Brachycaudus (Appelia) prunicola Kaltenbach, 1843)	P(c)	12		Colling 1955 [Great Britain]
Brachycaudus (Appelia) prunifex (Theobald, 1926)	P(c)	12		Blackman and Eastop 2015 [Great Britain]
Brachycaudus (Appelia) schwartzi (Börner, 1931)	P(c)	12		Gut 1976 [Holland], Gautam and Dhatwalia 2003 [Shimla, Himachal Pradesh, India]
Brachycaudus (Appelia) tragopogonis tragopogonis (Kaltenbach, 1843)	P(c) ?	12		Blackman and Eastop 1984 [?]
		11		Blackman and Eastop 1984 [Israel]
Brachycaudus (Appelia) tragopogonis setosus (Kaltenbach, 1843)	?	12		Blackman and Eastop 2015 [Iran]
Brachycaudus (Mordvilkomemor) amygdalinus (Schouteden, 1905)	P(c), P(o)	12		Kuznetsova 1968 [Georgia], Gautam and Kapoor 2002 [Una, Himachal Pradesh, India]
Brachycaudus (Mordvilkomemor) rumexicolens (Patch, 1917)	P(c)	12		Kurl and Chauhan 1987a [Barog, Himachal Pradesh, India]
Brachycaudus (Mordvilkomemor) sedi (Jacob, 1964)	P(c)	8		Blackman and Eastop 2006 [?]
Brachycaudus (Nevskyaphis) bicolor (Nevsky, 1929)	P(o), ?P(c)	12		Blackman and Eastop 2015 [?]
Brachycaudus (Nevskyaphis) malvae Shaposhnikov, 1964	?	12		Blackman and Eastop 2015 [?]
Brachycaudus (Prunaphis) cardui (Linnaeus, 1758)	P(c)	10		Kuznetsova 1968 [Georgia], Blackman and Eastop 1984 [?]
Brachycaudus (Prunaphis) jacobi Stroyan, 1957	P(c)	12		Gut 1976 [Holland]
Brachycaudus (Scrophulaphis) persicae (Passerini, 1860)	P(o), ?P(c)	10		Blackman and Eastop 1984 [?]
*Brachycolus cerastii* (Kaltenbach, 1846)	P(c)	14/13	XX/X0	Gut 1976 [Holland]
*Brachycorinella asparagi* (Mordvilko, 1929)	P(c)	10		Blackman and Eastop 1984 [?]
*Brachycorinella lonicerina* (Shaposhnikov, 1952)	P(c)	10		Blackman and Eastop 2006 [?]
*Brachyunguis calotropicus* Menon & Pawar, 1958	?	8		Kurl 1978, Kurl and Misra 1980, 1981 [Jodhpur, Rajasthan, India]
*Brachyunguis harmalae* Das, 1918	P(c), ?P(o)	8		Blackman and Eastop 2015 [?]
*Brachyunguis lycii* (Nevsky, 1928)	?	8		Blackman and Eastop 2006 [?]
*Brachyunguis tamaricis* (Lichtenstein, 1885)	P(c)	8		Blackman and Eastop 2015 [?]
*Brevicoryne brassicae* (Linnaeus, 1758)	P(c), P(o)	8, 9		Panigrahy and Patnaik 1991 [Chatrapur, Odisha, India]
		12, 14		Reeta Devi and Gautam 2012 [Kullu region, Himachal Pradesh, India]
		14		Kulkarni 1984 [Darjeeling, West Bengal, India]
		16/15	XX/X0	Cognetti 1961a, b, Cognetti and Cognetti-Varriale 1961, Pagliai 1962 [Italy]
		16		MacDonald and Harper 1965, Sun and Robinson 1966, Robinson and Chen 1969a [Canada], Kar et al. 1990 [India], Dutta and Gautam 1993 [Shimla, Himachal Pradesh, India], Kapoor and Gautam 1994 [Himachal Pradesh, India], Gautam and Dhatwalia 2003 [Hamirpur, Himachal Pradesh, India], Giannini et al. 2003 [Italy]
*Capitophorus carduinus* (Walker, 1850)	P(c)	16		Blackman and Eastop 2015 [?]
*Capitophorus cirsiiphagus* Takahashi, 1961	?P(c)	16		Blackman and Eastop 2006 (recorded as *Capitophorus elaeagni* in Blackman, 1986) [?]
*Capitophorus elaeagni* (Del Guercio, 1894)	P(c)	16		Robinson and Chen 1969a [Canada], Blackman 1986 [Japan]
*Capitophorus formosartemisiae* (Takahashi, 1921)	?	16		Blackman 1986 [Japan]
*Capitophorus hippophaes* (Walker, 1852)	P(c), ?P(o)	10		Sun and Robinson 1966, Robinson and Chen 1969a [Canada]
*Capitophorus hippophaes javanicus* Hille Ris Lambers, 1953	?	10		Chen and Zhang 1985a [Beijing area, China] (cited after Blackman and Eastop 2015), Blackman 1986 [Japan], Kar et al. 1990 [India]
*Capitophorus horni* Börner, 1931	P(c)	16		Blackman 1980 [Great Britain]
*Capitophorus inulae* (Passerini, 1860)	?P(o)	16		Blackman and Eastop 2015 [?]
*Capitophorus mitegoni* Eastop, 1956	?P(c)	9		Kurl and Chauhan 1986a, 1987a [Manali, Himachal Pradesh, India]
*Capitophorus pakansus* Hottes & Frison, 1931	P(c)	16		Blackman and Eastop 1994 [?]
*Capitophorus* sp. [?*eniwanus* Miyazaki, 1971]	?	10		Blackman and Eastop 2006 [China, near Beijing]
*Casimira canberrae* (Eastop, 1961)	P(c)	8		Blackman and Eastop 2015 [?]
*Catamergus kickapoo* (Hottes & Frison, 1931)	P(c)	10		Robinson and Chen 1969a (as *Macrosiphum*) [Canada]
*Cavariella aegopodii* (Scopoli, 1763)	P(c), P(o)	10		Blackman 1980 [Great Britain; Iran], Dutta and Gautam 1993, Gautam and Dhatwalia 2003 [Shimla, Himachal Pradesh, India]
		8, 9, 10		Dhatwalia and Gautam 2009 [Himachal Pradesh, India]
*Cavariella araliae* Takahashi, 1921	?P(c), P(o)	14		Blackman 1986 [Japan]
*Cavariella archangelicae* (Scopoli, 1763)	P(c)	6		Blackman 1980 [Great Britain]
*Cavariella borealis* Hille Ris Lambers, 1952	P(c)	6		Blackman and Eastop 1994 [?]
*Cavariella cicutae* (Koch, 1854)	P(c)	10		Blackman 1980 [Iran]
*Cavariella intermedia* Hille Ris Lambers, 1969	?P(c)	6		Blackman 1980 [Great Britain]
*Cavariella japonica* (Essig & Kuwana, 1918)	P(c)	8		Blackman 1986 [Japan]
*Cavariella konoi* Takahashi, 1939	P(c)	8		Blackman and Eastop 1984 [?], Blackman 1986 [Iceland]
*Cavariella pastinacae* (Linnaeus, 1758)	P(c)	8		Gut 1976 [Holland]
*Cavariella salicicola* (Matsumura, 1917)	P(c)	10		Chen and Zhang 1985b (cited after Blackman and Eastop 2015)
*Cavariella sericola* Shinji, 1927	?	8		Shinji 1927 [Japan]
*Cavariella theobaldi* (Gillette & Bragg, 1918)	P(c)	8, 10		Blackman 1980 [Great Britain]
Cavariella (Cavaraiellia) aquatica (Gillette & Bragg, 1916)	P(c)	8		Blackman and Eastop 2015 [?]
Cavariella (Cavariellinepicauda) oenanthi (Shinji, 1922)	?	8/7	XX/X0	Shinji 1931, Blackman 1986 [Japan] (based on n(♂) = 4(Shinji 1931))
*Cavariella* sp. 1	?	10		Robinson and Chen 1969a [Canada]
*Cavariella* sp. 2	?	6		Kuznetsova 1978 [St.Petersburg, Russia]
*Cavariella* sp. 3	?	10		Khuda-Bukhsh 1980 [Gharwal, Uttarakhand, India]
*Cavariella* sp. 4	?	12		Kar et al. 1990 [India]
*Ceruraphis eriophori* (Walker, 1848)	P(c)	14		Kuznetsova and Gandrabur 1991 [St. Petersburg, Russia] (they also noted that 2n=8 in Kuznetsova 1968, 1974 was erroneous)
*Chaetomyzus* sp.	?	12		Dutta and Gautam 1993 [Shimla, Himachal Pradesh, India]
*Chaetosiphon gracilicorne* David, Rajasingh & Narayanan, (1970) 1971	?P(c)	16		Dutta and Gautam 1993 [Shimla, Himachal Pradesh, India]
Chaetosiphon (Pentatrichopus) coreanum (Paik, 1965)	P(c)	8		Blackman and Eastop 1984 [?], Blackman 1986 [Japan]
Chaetosiphon (Pentatrichopus) fragaefolii (Cockerell, 1901)	P(o), ?P(c)	12, 13, 14, 15		Blackman et al. 1987 [Old World, North America]
		13, 14,15		Blackman and Eastop 2015 [?]
		14		Blackman and Eastop 1984 [?]
Chaetosiphon (Pentatrichopus) jacobi Hille Ris Lambers, 1953	P(o)	17		Blackman et al. 1987 [Western North America]
Chaetosiphon (Pentatrichopus) minor Forbes, 1884	P(c)	12		Blackman et al. 1987 [Eastern North America]
Chaetosiphon (Pentatrichopus) tetrarhodum (Walker, 1849)	P(c)	14		Blackman 1980 (as *Pentatrichopus*) [Great Britain]
		16		Blackman and Eastop 2006 [one sample from Australian Capital Territory, Australia]
Chaetosiphon (Pentatrichopus) thomasi Hille Ris Lambers, 1953	P(c)	12		Blackman et al. 1987 [Western North America]
*Chomaphis mira* Mordvilko, 1928	?	8		Kuznetsova and Shaposhnikov 1973 [Voronezh, Russia]
*Coloradoa artemisiae* (Del Guercio, 1913)	P(c)	16		Robinson and Chen 1969a [Canada]
*Coloradoa bournieri* Remaudière & Leclant, 1969	P(o)	22		Blackman and Eastop 2006 [?]
*Coloradoa huculaki* Szelegiewicz, 1981	?	c.24		Blackman and Eastop 2006 [immature specimen from China]
*Coloradoa ponticae* (Börner, 1940)	?	16		Blackman and Eastop 2015 [?]
*Coloradoa rufomaculata* (Wilson, 1908)	P(o), ?P(c)	8		Panigrahy and Patnaik 1987 (Chatrapur, Odisha, India)
		8, 17		Panigrahy and Patnaik 1991 [Chatrapur, Odisha, India]
		18		Das et al. 1985 [India]
*Coloradoa santolinae* Hille Ris Lambers, 1948	?	20		Blackman and Eastop 2006 [?] (specimens from *Artemisia monosperma*)
*Coloradoa viridis* (Nevsky, 1929)	?	16		Blackman and Eastop 2006 [?]
*Corylobium avellanae* (Schrank, 1801)	P(c)	10		Blackman and Eastop 1984 [?]
*Cryptaphis bromi* Robinson, 1967	P(c)	16		Robinson and Chen 1969a [Canada]
*Cryptaphis geranicola* (Shinji, 1935)	P(c)	14		Blackman 1986 [Japan]
*Cryptaphis poae* (Hardy, 1850)	P(c)	16		Sun and Robinson 1966 [Canada]
		20		Blackman and Eastop 1984 [?]
*Cryptomyzus alboapicalis* (Theobad, 1916)	P(c), P(o)	12		Blackman 1980 [Great Britain], Bašilova et al. 2008 [Lithuania]
*Cryptomyzus ballotae* Hille Ris Lambers, 1953	P(o), ?P(c)	12		Blackman 1980 [Great Britain]
*Cryptomyzus galeopsidis* (Kaltenbach, 1843)	P(c)	12		Blackman 1980 [Great Britain], Bašilova et al. 2008 [Lithuania]
*Cryptomyzus korschelti* Börner, 1938	P(c)	12		Bašilova et al. 2008 [Lithuania]
*Cryptomyzus leonuri* Bozhko, 1961	P(c)	12		Bašilova et al. 2008 [Lithuania]
*Cryptomyzus maudamanti* Guldemond, 1990	P(c)	12		Bašilova et al. 2008 [Lithuania]
*Cryptomyzus ribis* (Linnaeus, 1758)	P(c)	12		Sun and Robinson 1966, Robinson and Chen 1969a [Canada], Bašilova et al. 2008 [Lithuania]
*Cryptomyzus taoi* Hille Ris Lambers, 1963	P(c)	12		Blackman and Eastop 2015 [?]
*Cryptomyzus ulmeri* Börner, 1952	P(c)	12		Bašilova et al. 2008 [Lithuania]
Cryptomyzus (Ampullosiphon) stachydis (Heikenheimo, 1955)	P(c)	12		Blackman and Eastop 2006 [?]
*Cryptosiphum artemisiae* Buckton, 1879	P(c)	8		Blackman 1980 [Great Britain], Blackman 1986 [Japan]
*Delphiniobium canadense* (Robinson, 1968)	P(c)	20		Blackman and Eastop 2006 [?]
*Delphiniobium yezoense* Miyazaki, 1971	P(c)	12		Chen and Zhang 1985a [Beijing area, China] (cited after Blackman and Eastop 2015)
*Diuraphis mexicana* (Baker, 1934)	P(o), ?P(c)	8		Blackman and Eastop 2006 [?]
*Diuraphis noxia* (Mordvilko ex Kurdjumov, 1913)	P(c), ?P(o)	10		Blackman 1980 [South Africa]
		10/9	XX/X0	Novotná et al. 2011 [Czech Republic]
Diuraphis (Holcaphis) agrostidis (Muddathir, 1965)	P(c)	12		Blackman 1980 (as Holcaphis) [Great Britain]
Diuraphis (Holcaphis) frequens (Walker, 1848)	P(c)	14		Gut 1976 [Holland]
Diuraphis (Holcaphis) holci (Hardy, 1850)	P(c)	14		Gut 1976 [Holland]
*Dysaphis affinis* (Mordvilko, 1928)	P(c)	12		Kuznetsova 1968 [Georgia]
*Dysaphis angelicae* (Koch, 1854)	P(c)	12		Blackman and Eastop 2015 [?]
*Dysaphis anthrisci anthrisci* Börner, 1950	P(c)	12/11	XX/X0	Kuznetsova 1968 [St. Petersburg, Russia], Kuznetsova and Gandrabur 1991 [Ukraine]
		12		Gautam and Kapoor 2002 [Shimla, Himachal Pradesh, India]
*Dysaphis anthrisci majkopica* Shaposhnikov, 1961	P(c)	12		Kuznetsova 1968 [North Caucasus, Russia], Kuznetsova 1974 [?]
*Dysaphis apiifolia* (Theobald, 1923)	P(o), P(c)	12		Blackman 1980 [Iran]
*Dysaphis chaerophyllina* Shaposhnikov, 1959	P(c)	12	XX/X0	Kuznetsova 1968 [North Caucasus, Russia], Kuznetsova 1974 [?]
*Dysaphis crataegi crataegi* (Kaltenbach, 1843)	P(c), P(o)	12		Blackman 1980 [Great Britain]
*Dysaphis crataegi heracleana* (Narzikulov, 1955)	?	12		Kuznetsova and Daniyarova1980 [Kondara, Tajikistan]
*Dysaphis devecta* (Walker, 1849)	P(c)	12/11	XX/X0	Kuznetsova and Gandrabur 1991 [St.Petersburg, Russia]
*Dysaphis foeniculus foeniculus* (Theobald, 1923)	P(o)	12		Blackman and Eastop 1984 [?], Gautam and Kapoor 2002 [Shimla, Himachal Pradesh, India]
*Dysaphis foeniculus malidauci* Shaposhnikov, 1976	P(c)	12		Kuznetsova 1968 [Alma-Ata, Kazakhstan]
*Dysaphis hirsutissima* (Börner, 1940)	P(c)	12		Kuznetsova 1968 [St.Petersburg, Russia]
*Dysaphis narzikulovi* Shaposhnikov, 1956	P(c)	12		Kuznetsova and Daniyarova1980 [Kondara, Tajikistan]
*Dysaphis newskyi aizenbergi* (Shaposhnikov, 1949)	P(c)	12		Kuznetsova 1968 (as *Dysaphis aizenbergi* (Shaposhnikov, 1949)) [St.Petersburg, Russia]
*Dysaphis radicola* (Mordvilko, 1897)	P(c)	12		Kuznetsova 1968 [St.Petersburg, Russia], Blackman 1980 [Great Britain]
*Dysaphis rumecicola* (Hori, 1927)	P(c), P(o)	12		Kuznetsova and Daniyarova 1980 (as *Dysaphis emicis* Mim.) [Kondara, Tajikistan]
*Dysaphis tulipae* (Boyer de Fonscolombe, 1841)	P(o)	11, 12		Blackman 1980 [Great Britain]
Dysaphis (Cotoneasteria) microsiphon (Nevsky, 1929)	P(c)	12		Kuznetsova 1968 [Georgia]
Dysaphis (Pomaphis) aucupariae (Buckton, 1879)	P(c)	12		Blackman and Eastop 1994 [?], Blackman and Eastop 2006 [?]
Dysaphis (Pomaphis) maritima (Hille Ris Lambers, 1955)	P(c)	12		Blackman and Eastop 2006 [?]
Dysaphis (Pomaphis) pavlovskyana Narzikulov, 1957	P(c)	12		Khuda-Bukhsh and Pal 1983a [Garhwal, Uttarakhand, India] (apparently it is *Dysaphis indica* Chakrabarti & Medda, 1993)
Dysaphis (Pomaphis) plantaginea (Passerini, 1860)	P(c)	12		Kuznetsova 1968 (as *Dysaphis mali* (Ferrari, 1872) [Crimea, Ukraina], Blackman 1986 [Japan], Criniti et al. 2009 [Italy]
Dysaphis (Pomaphis) pyri (Boyer de Fonscolombe, 1841)	P(c)	12		Kuznetsova 1968 [Crimea, Ukraina]
Dysaphis (Pomaphis) reamuri Mordvilko, 1928	P(c)	12		Kuznetsova 1968 [Crimea, Ukraina]
Dysaphis (Pomaphis) sorbi (Kaltenbach, 1843)	P(c)	12		Kuznetsova 1968 [St.Petersburg, Russia]
*Dysaphis* sp.	?	12		Kuznetsova 1968 (as *Dysaphis crataegi* (Kaltenbach, 1843)) [Georgia]
*Elatobium abietinum* (Walker, 1849)	P(c), P(o)	18		Blackman 1980 [Great Britain]
*Elatobium* sp.	?	8		Khuda-Bukhsh and Kar 1990 [Shillong, Meghalaya, India]
*Ericaphis fimbriata* (Richards, 1959)	P(c)	14		Blackman and Eastop 2015 [?]
*Ericaphis gentneri* (Mason, 1947)	P(c)	18, 19, 20, 21, 23/17, 19		Blackman and Eastop 2015 [British Columbia, Canada]
*Ericaphis scammelli* (Mason, 1940)	P(c)	14		Blackman and Eastop 2015 [?]
*Ericaphis wakibae* (Hottes, 1934)	P(c)	12		Blackman and Eastop 2015 [?]
*Ericolophium holsti* (Takahashi, 1935)	?	22		Dutta and Gautam 1993 [Shimla, Himachal Pradesh, India]
*Ericolophium itoe* (Takahashi, 1925)	?	18		Blackman and Eastop 2006 [?]
*Eucarazzia elegans* (Ferrari, 1872)	?	12		Gautam and Kapoor 2002 [Shimla, Himachal Pradesh, India]
*Eumyzus eastopi* Maity & Chakrabarti ex Maity, Bhattacharya & Chakrabarti, 1982	?	10		Khuda-Bukhsh and Pal 1986b [Triyuginarayan, Uttarakhand, India]
*Eumyzus gallicola* Takahashi, 1963	?	12		Blackman 1986 [Japan]
*Eumyzus impatiensae* (Shinji, 1924)	P(c)	10		Pal and Khuda-Bukhsh 1980, Khuda-Bukhsh and Pal 1986b [Triyuginarayan, Uttarakhand, India]
		12		Blackman 1986 [Japan]
*Gypsoaphis oestlundi* Hottes, 1930	?	4		Sun and Robinson 1966 [Canada], Robinson and Chen 1969a [Canada]
*Hayhurstia atriplicis* (Linnaeus, 1761)	P(c)	14		Sun and Robinson 1966 (as *Brachycolus*), Robinson and Chen 1969a [Canada], Chen and Zhang 1985a [Beijing area, China] (cited after Blackman and Eastop 2015), Dutta and Gautam 1993 [Shimla, Himachal Pradesh, India]
*Hyadaphis coriandri* (B. Das, 1918)	P(c), ?P(o)	12		Blackman and Eastop 2006 [?]
		13		Blackman 1980 [Iran]
		14		Kuznetsova and Shaposhnikov 1973 (as *Semiaphis tataricae* (Aizenberg, 1935) [St. Petersburg, Russia]
*Hyadaphis foeniculi* (Passerini, 1860)	P(c)	12, 14		Blackman and Eastop 2006 [?] (one sample from *Foeniculum* had a mixture of 2n=12 and 2n=14 individuals)
		13		Blackman and Eastop 2006 [?] (one sample from *Lonicera*, a *foeniculi* × *passerinii* hybrid?)
		14		Gut 1976 [Holland] (on *Conium maculatum*), Gautam and Kapoor 2002 [Una, Himachal Pradesh, India], Blackman and Eastop 2006 [?] (for samples of *Hyadaphis foeniculi* from *Conium* and *Foeniculum*)
*Hyadaphis passerinii* (del Guercio, 1911)	P(c), P(o)	12		Kuznetsova and Shaposhnikov 1973 [Crimea, Ukraine]
*Hyadaphis tataricae* (Aizenberg, 1935)	P(c)	14		Blackman and Eastop 2015 [?]
*Hyadaphis* sp.	?	12		Blackman 1980 [Great Britain]
*Hyalomyzus raoi* Hille Ris Lambers, 1973	?	8		Khuda-Bukhsh and Kar 1990 [Shillong, Meghalaya, India]
*Hyalopteroides humilis* (Walker, 1852)	P(c)	16		Blackman 1980 [Great Britain]
*Hyalopterus amygdali* (Blanchard, 1840)	P(c)	10		Chen and Zhang 1985a [Beijing area, China] (cited after Blackman and Eastop 2015)
*Hyalopterus pruni* (Geoffroy, 1762)	P(c)	10		Shibata 1941 [Japan], Robinson and Chen 1969a [Canada], Kuznetsova and Shaposhnikov 1973 [St.Petersburg, Russia; Turkmenistan], Kuznetsova 1974 [?], Pal and Khuda-Bukhsh 1982, Khuda-Bukhsh and Pal 1984a [Srinagar, Jammu and Kashmir, India], Blackman 1986 [Japan]
*Hyperomyzus carduellinus* (Theobald, 1915)	P(o)	12		Kurl and Chauhan 1986a [Dharampur, Himachal Pradesh, India], Kurl and Chauhan 1987a [Naldehra, Himachal Pradesh, India], Gautam and Kapoor 2002 [Una, Himachal Pradesh, India]
*Hyperomyzus lactucae* (Linnaeus, 1758)	P(c), P(o)	12		Colling 1955 [Great Britain], Sun and Robinson 1966 (as *Nasonovia*), Robinson and Chen 1969a [Canada], Dutta and Gautam 1993 [Shimla, Himachal Pradesh, India]
*Hyperomyzus lampsanae* (Börner, 1932)	P(c)	12		Blackman 1980 [Great Britain]
Hyperomyzus (Hyperomyzella) rhinanthi (Schouteden, 1903)	P(c)	12		Blackman and Eastop 2015 [?]
Hyperomyzus (Neonasonovia) picridis (Börner & Blunck, 1916)	P(c)	12		Blackman 1980 [Great Britain], Blackman and Eastop 1984 [?]
Hyperomyzus (Neonasonovia) ribiellus (Davis, 1919)	P(c)	12		Sun and Robinson 1966 (as *Amphorophora*), Robinson and Chen 1969a (as *Kakimia ribiella* (Davis, 1919))[Canada], Blackman and Eastop 1984 [?]
*Hysteroneura setariae* (Thomas, 1878)	P(c), P(o)	12		Robinson and Chen 1969a [Canada], Kurl 1986 [Meghalaya, India], Khuda-Bukhsh and Kar 1990 [Kalyani, West Bengal, India], Kapoor and Gautam 1994 [Shimla, Himachal Pradesh, India]
*Idiopterus nephrelepidis* Davis, 1909	P(o)	12		Blackman and Spence 1996 [Great Britain]
		13		Blackman 1980 [Great Britain]
*Illinoia alni* (Mason, 1925)	P(c)	10		Blackman 1980 [Canada]
*Illinoia azaleae* (Mason, 1925)	P(o), ?P(c)	10		Blackman and Eastop 1984 [?]
*Illinoia liriodendri* (Monell, 1879)	P(c)	10		Blackman 1980 [USA]
*Illinoia morrisoni* (Swain, 1918)	P(o)	10		Blackman and Eastop 2015 [?]
*Illinoia pepperi* (MacGillivray, 1958)	P(c)	22		Blackman and Eastop 2000 [?]
*Illinoia richardsi* (MacGillivray, 1958)	?	10		Blackman 1980 [Canada]
*Illinoia spiraeae* (MacGillivray, 1958)	?	10		Blackman and Eastop 2015 [?]
*Illinoia subviride* (MacDougall, 1926)	?	10		Blackman and Eastop 2006 [?]
*Illinoia wahnaga* (Hottes, 1952)	P(c)	10		Sun and Robinson 1966 (as *Masonaphis*), Robinson and Chen 1969a (as *Masonaphis*) [Canada]
Illinoia (Amphorinophora) crystleae (Smith & Knowlton, 1939)	P(c)	16		Blackman and Eastop 2006 [?]
Illinoia (Masonaphis) lambersi (MacGillevray, 1960)	P(c), P(o)	10		Gut 1976 (as *Masonaphis*) [Holland]
Illinoia (Masonaphis) menziesiae (Robinson, 1969)	P(c)	10		Blackman and Eastop 2006 [?]
Illinoia (Oestlundia) davidsoni Mason, 1925	?	12		Blackman 1980 [USA]
Illinoia (Oestlundia) maxima (Mason, 1925)	P(c)	12		Blackman 1980 [Canada]
Illinoia (Oestlundia) rubicola (Oestlund, 1886)	P(c)	12		Shinji 1931 (as *Amphorophora rubicola* (Oestlund) [?USA], Robinson and Chen 1969a (as *Masonaphis*) [Canada]
*Impatientinum asiaticum asiaticum* Nevsky, 1929	P(c)	16		Gut 1976 [Holland], Pal and Khuda-Bukhsh 1980 [Sonprayag, Uttarakhand, India], Khuda-Bukhsh and Pal 1986b [Gourikund, Uttarakhand, India], Kapoor and Gautam 1994 [Nahan, Himachal Pradesh, India]
*Impatientinum asiaticum dalhousiensis* Verma, 1969	?P(c)	16		Kurl and Chauhan 1986a, 1987a [Mecloadganj, Himachal Pradesh, India]
*Impatientinum balsamines* (Kaltenbach 1862)	P(c)	16		Blackman and Eastop 2015 [?]
*Impatientinum impatiens* (Shinji, 1922)	?P(c)	16		Blackman 1986 [Japan]
*Indoidiopterus geranii* (Chowdhuri, R.C. Basu, Chakrabarti, & D.N. Raychaudhuri, 1969)	P(c)	12		Pal and Khuda-Bukhsh 1980, Khuda-Bukhsh and Pal 1986b [Triyuginarayan, Uttarakhand, India]
*Indomasonaphis inulae* (A.K.Ghosh & Raychaudhuri, 1972)	P(c)	30		Kurl and Chauhan 1986c [Barog, Himachal Pradesh, India]
		32		Kurl and Chauhan 1987a [Barog, Himachal Pradesh, India]
*Indomegoura indica* (van der Goot, 1916)	P(c)	10		Blackman and Eastop 2006 [?]
		12		Shinji 1927 (as *Amphorophora indicum*), Blackman 1986 [Japan] (based on n(♂) = 6 (Shinji 1927))
*Liosomaphis atra* Hille Ris Lambers, 1966	?	17		Kurl and Chauhan 1987b [Barog, Himachal Pradesh, India], Kurl and Chauhan 1988 [India]
		18		Kurl and Chauhan 1987a [Barog, Himachal Pradesh, India]
*Liosomaphis berberidis* (Kaltenbach, 1843)	P(c)	18		Blackman 1980 [Great Britain]
*Liosomaphis himalayensis* A.N. Basu, 1964	?P(c)	18		Pal and Khuda-Bukhsh 1984 [Jamunetri, Uttarakhand, India], Dutta and Gautam 1993 [Shimla, Himachal Pradesh, India]
*Lipaphis erysimi* (Kaltenbach, 1843)	P(c)	10		Gut 1976 [Holland]
*Lipaphis fritzmuelleri* Börner, 1950	P(c)	10		Blackman and Eastop 2015 [?]
*Lipaphis pseudobrassicae* (Davis, 1914)	P(c), P(o)	6/5	XX/X0	Fox 1957 [Virginia, USA]
		8		Chen and Zhang 1985a, c (as *Lipaphis erysimi*) [Beijing area, China] (Chen and Zhang 1985a cited after Blackman and Eastop 2015), Kar and Khuda-Bukhsh 1991b (as *Lipaphis erysimi*) [Kalyani, West Bengal, India]
		8–9		Blackman and Eastop 2015 [?] (anholocyclic populations in most parts of the world have 2n=9)
		8, 10		Gautam and Kapoor 2002 (as *Lipaphis erysimi*) [Una, Himachal Pradesh, India]
		8, 9, 10		Feng and You 1988 (as *Lipaphis erysimi*) [Taiwan]
		9, 10		Kurl 1986 (as *Lipaphis erysimi*) [Meghalaya, India]
		10		Kurl and Misra 1981(as *Lipaphis erysimi*) [Jodhpur, Rajasthan, India], Gautam and Sharma 1990 (as *Lipaphis erysimi*) [Himachal Pradesh, India], Gautam and Dhatwalia 2003 (as *Lipaphis erysimi*) [Shimla, Himachal Pradesh, India]
		4, 5, 6, 7, 8, 9, 10, 15, 18		Khuda-Bukhsh and Pal 1984b (as *Lipaphis erysimi*) [Kalyani, West Bengal, India]
*Longicaudus trirhodus* (Walker,1849)	P(c)	12		Gut 1976 [Holland], Chen and Zhang 1985a [Beijing area, China] (cited after Blackman and Eastop 2015)
*Macchiatiella itadori* (Shinji, 1924)	P(c)	12/11	XX/X0	Shinji 1927, 1931, 1941a (as *Acaudus*), Blackman 1986 [Japan] (based on n(♂) = 6 (Shinji 1927, 1931))
*Macromyzus woodwardiae* (Takahashi, 1921)	P(o), ?P(c)	12		Blackman 1986 [Japan]
*Macrosiphoniella absinthii* (Linnaeus, 1758)	P(c)	12		Sun and Robinson 1966, Robinson and Chen 1969a [Canada]
*Macrosiphoniella artemisiae* (Boyer de Fonscolombe, 1841)	P(c)	12		Gut 1976 [Holland]
*Macrosiphoniella dimidiata* Börner, 1942	P(c)	12		Blackman and Eastop 2006 [?]
*Macrosiphoniella formosartemisiae* Takahashi, 1921	?P(c), P(o)	10		Pal and Khuda-Bukhsh 1980, Khuda-Bukhsh and Pal 1986b [Rambara, Uttarakhand, India]
*Macrosiphoniella huaidensis* G. Zhang, 1980	?	12		Chen and Zhang 1985b (cited after Blackman and Eastop 2015)
*Macrosiphoniella kikungshana* Takahashi, 1937	P(c)	12		Pal and Khuda-Bukhsh 1980, Khuda-Bukhsh and Pal 1986b [Triyuginarayan, Uttarakhand, India]
*Macrosiphoniella ludovicianae* (Oestlund, 1886)	P(c)	12		Robinson and Chen 1969a [Canada]
*Macrosiphoniella millefolii* (De Geer, 1773)	P(c)	12		Gut 1976 [Holland]
*Macrosiphoniella pseudoartemisiae* Shinji, 1933	?	10		Pal and Khuda-Bukhsh 1982, Khuda-Bukhsh and Pal 1986b [Srinagar, Jammu and Kashmir, India], Dutta and Gautam 1993 [Solan, Himachal Pradesh, India]
		12		Kar and Khuda-Bukhsh 1989 [Kalimpong, West Bengal, India] (Blackman and Eastop 2006: "perhaps this was misidentified yomogifoliae?")
*Macrosiphoniella sanborni* (Gillette, 1908)	P(o)	10		Chen and Zhang 1985a [Beijing area, China] (cited after Blackman and Eastop 2015), Chen and Zhang 1985b (cited after Blackman and Eastop 2015)
*Macrosiphoniella sanborni* (Gillette, 1908)	P(o)	12		Boschetti 1963 [Italia], Blackman and Eastop 2015 [many samples from Great Britain and India], Khuda-Bukhsh and Datta 1981b [India], Gautam and Sharma 1990 [Himachal Pradesh, India], Dutta and Gautam 1993 [Shimla, Himachal Pradesh, India], Blackman and Eastop 2006 [one sample from China]
*Macrosiphoniella sejuncta* (Walker, 1848)	P(c)	10		Blackman 1980 [Great Britain]
*Macrosiphoniella subterranea* (Koch, 1855)	P(c)	12		Gut 1976 (as *Macrosiphoniella trimaculata* Hille Ris Lambers, 1938) [Holland]
*Macrosiphoniella szalaymarzsoi* Szelegiewicz, 1978	?	12		Blackman and Eastop 2006 [?]
*Macrosiphoniella tanacetaria* (Kaltenbach, 1843)	P(c)	12		Sun and Robinson 1966, Robinson and Chen 1969a [Canada]
		12/11	XX/X0	Kuznetsova and Gandrabur 1991 [St. Petersburg, Russia]
*Macrosiphoniella tapuskae* (Hottes & Frison, 1931)	P(c)	12		Blackman and Eastop 2015 [?]
*Macrosiphoniella yomogifoliae* (Shinji, 1922)	?	12		Kulkarni 1984 (as *Macrosiphum yamagopholiae* (Shinji)) [Darjeeling, West Bengal, India]
Macrosiphoniella (Asterobium) yomenae (Shinji, 1922)	?	12		Shinji 1927 (as *Amphorophora*), Blackman 1986 [Japan] (based on n(♂) = 6 (Shinji 1927))
Macrosiphoniella (Chosoniella) myohyangsani Szelegiewicz, 1980	?	12		Chen and Zhang 1985b (cited after Blackman and Eastop 2015)
Macrosiphoniella (Chosoniella) spinipes A.N. Basu, 1968	?	10		Kar et al. 1990 [India]
Macrosiphoniella (Phalangomyzus) antennata Holman & Szelegiewicz, 1978	?	12		Blackman and Eastop 2006 [?]
Macrosiphoniella (Phalangomyzus) grandicauda Takahashi & Moritsu, 1963	?	12		Chen and Zhang 1985b (cited after Blackman and Eastop 2015)
Macrosiphoniella (Phalangomyzus) oblonga (Mordvilko, 1901)	P(c)	12		Gut 1976 [Holland]
Macrosiphoniella (Phalangomyzus) persequens (Walker, 1852)	P(c)	12		Gut 1976 [Holland]
*Macrosiphoniella* sp. 1	?	12		Samkaria et al. 2010 [Shimla, Himachal Pradesh, India]
*Macrosiphoniella* sp. 2	?	12		Chen and Zhang 1985a [Beijing area, China] (cited after Blackman and Eastop 2015)
*Macrosiphum albifrons* Essig, 1911	P(c)	10		Blackman 1980 [USA]
*Macrosiphum californicum* (Clarke, 1903)	P(c)	10		Blackman 1980 [USA]
*Macrosiphum centranthi* Theobald, 1915	P(c), ?P(o)	10		Blackman and Eastop 1984 [?]
*Macrosiphum cholodkovskyi* (Mordvilko, 1909)	P(c)	10		Blackman and Eastop 2015 [?]
*Macrosiphum claytoniae* Jensen, 2000	P(o)	16		Blackman and Eastop 2015 [?]
*Macrosiphum clematifoliae* Shinji, 1924	P(c)	18		Blackman 1986 [Japan], Blackman and Eastop 2015 [?] (*the karyotype suggests that this species may be a Sitobion*)
*Macrosiphum clydesmithi* Robinson, 1980	P(c)	16		Blackman and Eastop 2015 [?]
*Macrosiphum cornifoliae* (Shinji, 1924)	?P(c)	14/13	XX/X0	Shinji 1927, 1931, 1941a, Blackman 1986 [Japan] (based on 2n male =13 (Shinji 1931))
*Macrosiphum corydalis* (Oestlund, 1886)	P(c)	10		Blackman and Eastop 2015 [?]
*Macrosiphum creelii* Davis, 1914	?P(c)	10		Blackman and Eastop 2015 [?]
*Macrosiphum daphinidis* Börner, 1940	P(c)	10		Blackman and Eastop 2015 [?]
*Macrosiphum dicentrae* Jensen & Chan, 2009	P(c)	16		Blackman and Eastop 2015 [?]
*Macrosiphum equiseti* (Holman, 1961)	P(c)	16		Blackman and Eastop 2015 [?]
*Macrosiphum euphorbiae* (Thomas, 1878)	P(c), P(o)	10/9	XX/X0	Lawson 1936 (as *Macrosiphum solanifolii* Ashmead, 1882) [USA], Dionne and Spicer 1957 (as *Macrosiphum solanifolii*) [Canada], Pagliai 1966 [Italy], Sun and Robinson 1966, Robinson and Chen 1969a [Canada], Gautam and Kapoor 2002 [Shimla, Himachal Pradesh, India], Monti et al. 2011 [Italy]
*Macrosiphum euphorbiellum* Theobald, 1917	P(c)	10		Blackman 1980 (as *Macrosiphum amygdaloides* Theobald, 1925) [Great Britain]
*Macrosiphum funestum* (Macchiati, 1885)	P(c)	10		Blackman 1980 [Great Britain]
*Macrosiphum gei* (Koch, 1855)	P(c)	10		Gut 1976 [Holland]
*Macrosiphum geranii* (Oestlund, 1887)	?	10		Robinson and Chen 1969a [Canada]
*Macrosiphum hamiltoni* Robinson, 1968	?	10		Robinson and Chen 1969a [Canada]
*Macrosiphum hellebori* Theobald & Walton, 1923	P(c), P(o)	10		Gut 1976 [Holland]
*Macrosiphum impatientis* Williams, 1911	P(c)	10		Blackman and Eastop 2015 [?]
*Macrosiphum knautiae* Holman 1972	P(c)	12	XX/X0	Voronova et al. 2010 [Byelorussia]
*Macrosiphum manitobense* Robinson, 1965	P(c)	10		Sun and Robinson 1966, Robinson and Chen 1969a [Canada]
*Macrosiphum mordvilkoi* Miyazaki, 1968	P(c)	10		Blackman 1986 [Japan]
*Macrosiphum occidentalis* (Essig, 1942)	P(c)	16		Blackman and Eastop 2015 [?]
*Macrosiphum opportunisticum* Jensen, 2012	P(c)	16		Blackman and Eastop 2015 [?]
*Macrosiphum osmaroniae* Wilson, 1912	P(c)	16		Blackman and Eastop 2006 [?]
*Macrosiphum pachysiphon* Hille Ris Lambers, 1966	?	18		Kurl 1980b [Meghalaya, India], Gautam and Kapoor 2002 [Shimla, Himachal Pradesh, India]
*Macrosiphum pallidum* (Oestlund, 1887)	?	10		Robinson and Chen 1969a [Canada], Gautam and Dhatwalia 2003 [Shimla, Himachal Pradesh, India]
*Macrosiphum parvifolii* Richards, 1967	?	16		Blackman and Eastop 2015 [?]
*Macrosiphum penfroense* Stroyan, 1979	?P(o)	10		Blackman and Eastop 2015 [?]
*Macrosiphum ptericolens* Patch, 1919	P(c)	16		Blackman 1980 (as *Sitobion*) [Great Britain; USA]
*Macrosiphum pteridis* Wilson, 1915	P(c)	16		Blackman and Eastop 2015 [?]
*Macrosiphum pyrifoliae* MacDougall, 1926	?P(c)	10		Blackman and Eastop 2015 [?]
		11		Blackman and Eastop 2015 [?]
		12		Blackman and Eastop 1994 [?]
*Macrosiphum rhamni* (Clarke, 1903)	P(c)	16		Blackman and Eastop 2015 [?]
*Macrosiphum rosae* (Linnaeus, 1758)	P(c), P(o)	10		Stevens 1905b, 1906, 1909 (as *Aphis*) [USA], Hewitt 1906 (as *Aphis*) [Great Britain], Baehr 1909 (as *Aphis*) [Germany], Cognetti 1961a, b, Cognetti and Cognetti-Varriale 1961 Boschetti and Pagliai 1964, Pagliai 1966 [Italy], Khuda-Bukhsh 1980 [Garhwal, Uttarakhand, India], Raychaudhuri and Das 1987 [India], Kar and Khuda-Bukhsh 1989 [Kalimpong, West Bengal, India], Gautam and Dhatwalia 2003, Samkaria et al. 2010 [Shimla, Himachal Pradesh, India], Reeta Devi and Gautam 2012 [Kullu region, Himachal Pradesh, India]
		12		Kulkarni 1984 [Darjeeling, West Bengal, India]
		14		Stschelkanovzew 1904 (as *Aphis*) [Germany]
*Macrosiphum stanleyi* Wilson, 1915	P(c)	16		Blackman and Eastop 1994 [?]
*Macrosiphum stellariae* Theobald, 1913	?	10		Blackman 1980 [Great Britain]
*Macrosiphum tenuicauda* Bartholomew, 1932	?	10		Blackman and Eastop 2006 [?]
*Macrosiphum tinctum* (Walker, 1849)	P(o)	10		Blackman and Eastop 2015 [?]
*Macrosiphum walkeri* Robinson, 1980	P(o), ?P(c)	16		Blackman and Eastop 2015 [?]
*Macrosiphum willamettense* Jensen, 2000	P(c)	10		Blackman and Eastop 2006 [?]
*Macrosiphum woodsiae* Robinson, 1980	?P(c)	16		Blackman and Eastop 2006 [?]
Macrosiphum (Neocorylobium) pseudocoryli Patch, 1919	P(c)	10		Blackman and Eastop 1994 [?]
Macrosiphum (Unisitobion) perillae (G. Zhang, 1988)	P(c)	18		Chen and Zhang 1985a [Beijing area, China] (cited after Blackman and Eastop 2015)
*Macrosiphum* sp.	?	10		Robinson and Chen 1969a [Canada]
*Matsumuraja capitophoroides* Hille Ris Lambers, 1966	?P(c)	14		Kurl and Chauhan 1986 [Manali, Himachal Pradesh, India]
*Macrosiphum nuditerga* Hille Ris Lambers, 1965	?	14		Blackman and Eastop 2015 [?]
*Macrosiphum rubea* Sorin, 1965	?	14		Blackman 1986 [Japan]
*Macrosiphum rubi* (Matsumura, 1918)	P(c)	14		Blackman 1986 [Japan]
*Macrosiphum rubifoliae* Takahashi, 1931	P(c), P(o)	14		Blackman 1986 [Japan]
*Macrosiphum rubiphila* Takahashi, 1965	?	14		Blackman and Eastop 2015 [?]
*Matsumuraja* sp.	?	18		Blackman and Eastop 2006 [?]
*Megoura crassicauda* Mordvilko, 1919	?	10		Blackman and Eastop 2015 [?]
*Megoura dooarsis* (A.K. Ghosh & D.N. Raychaudhuri, 1969)	?	20		Dutta and Gautam 1993 [Shimla, Himachal Pradesh, India]
*Megoura lespedezae* (Essig & Kuwana, 1918)	?	12/11	XX/X0	Shinji 1927, 1931 (as *Amphorophora*), Blackman 1986 [Japan] (based on 2n (♂) =6 (Shinji 1931))
		14		Kulkarni and Kacker 1980, 1981b [India], Blackman and Eastop 1984 [?], Shinji 1941a (as *Myzus lespedezae*), Blackman 1986 [Japan]
*Megoura viciae* Buckton, 1876	P(c)	10/9	XX/X0	Manicardi, Gautam et al. 1991 [Italy]
		10		Pagliai 1966, Orlando 1974, 1983 [Italy], Blackman 1986 [Japan]
*Melanaphis arundinariae* (Takahashi, 1937)	?	8		Khuda-Bukhsh and Pal 1984a [Triyuginarayan, Uttarakhand, India], Kar and Khuda-Bukhsh 1989 [Shillong, Meghalaya, India]
*Melanaphis bambusae* (Fullaway, 1910)	P(c), P(o)	8		Blackman and Eastop 1984 [?], Blackman 1986 [Japan]
		10		Kuznetsova and Shaposhnikov 1973 [Sukhumi, Georgia], Kuznetsova 1974 [?]
		12		Kar et al. 1990 [India]
*Melanaphis donacis* (Passerini, 1861)	P(c)	8		Pal and Khuda-Bukhsh 1980, Khuda-Bukhsh and Pal 1984a [Ghangaria, Uttarakhand, India], Kuznetsova and Shaposhnikov 1973 (as *Longiunguis*) [Kara-Kala, Turkmenistan]
*Melanaphis japonica* (Takahashi, 1919)	P(c)	c.22		Blackman and Eastop 2006 [?]
*Melanaphis meghalayensis meghalayensis* D.N. Raychaudhuri & C. Banerjee, 1974	?	10		Pal and Khuda-Bukhsh 1980, Khuda-Bukhsh and Pal 1984a [Gobindoghat, Uttarakhand, India]
*Melanaphis pyraria* (Passerini, 1861)	P(c)	8		Kuznetsova and Shaposhnikov 1973 (as *Longiunguis*) [Crimea, Ukraine], Gautam and Dhatwalia 2003 [Shimla, Himachal Pradesh, India], Criniti et al. 2009 [Italy]
*Melanaphis sacchari* (Zehntner, 1897)	P(o), ?P(c)	8		Blackman 1980 [India], Blackman 1986 [Hong Kong]
		10		Khuda-Bukhsh and Kar 1990 [Kalyani, West Bengal, India]
*Melanaphis sorghi* (Theobald, 1904)	P(o), ?P(c)	8		Blackman and Eastop 2015 [?]
*Melanaphis* sp.	?	22		Blackman 1986 [Japan] (Blackman and Eastop 2015: "a record of 2n=22 for *Melanaphis sacchari* in Japan (Blackman 1986) is referable to another, undescribed species")
*Metopeurum fuscoviride* Stroyan, 1950	P(c)	8		Blackman 1980 [Great Britain]
*Metopolophium albidum* Hille Ris Lambers, 1947	P(c)	16		Blackman and Eastop 2006 [?]
*Metopolophium dirhodum* (Walker, 1849)	P(c), P(o)	16, 18		Rubín de Celis et al. 1997 [Brazil]
		18		Sun and Robinson 1966 (as *Acyrthosiphon*), Robinson and Chen 1969a [Canada], De Barro 1992 [Australia]
*Metopolophium fasciatum* Stroyan, 1982	P(o), ?P(c)	18		Blackman and Eastop 2015 [?]
*Metopolophium festucae festucae* (Theobald, 1917)	P(c), P(o)	16		Blackman and Eastop 1984 [?]
*Metopolophium festucae cerealium* Stroyan, 1982	P(c), P(o)	16		Blackman and Eastop 1984 [?]
*Metopolophium festucae* Hille Ris Lambers, 1947	P(c)	16		Blackman 1980 [Great Britain]
*Metopolophium* sp.	?	16		Kar et al. 1990 [India] (possible *Acyrthosiphum*)
*Microlophium carnosum* (Buckton, 1876)	P(c)	16		Kuznetsova and Shaposhnikov 1973 (as *Microlophium evansi* (Theobald, 1923) [Crimea, Ukraine]
		18		Robinson and Chen 1969a [Canada]
		20		Blackman 1980 [Great Britain], Blackman 2010 [?]
*Microlophium rubiformosanum* (Takahashi, 1927)	?	12		Pal and Khuda-Bukhsh 1982, Khuda-Bukhsh and Pal 1986b (as *Acyrthosiphum*) [Srinagar, Jammu and Kashmir, India]
*Microlophium?sibiricum tenuicauda* Hille Ris Lambers, 1949	?	18		Blackman 1980 [North America]
*Microlophium* sp.	?	16		Blackman and Eastop 2006 [?] ("This is possibly the species with 2n=16 from Crimea listed as *Microlophium evansi* Theobald by Kuznetsova and Shaposhnikov (1973)), Blackman 2010 [Great Britain]
*Micromyzella filicis* (van der Goot, 1917)	?	36		Blackman and Eastop 2015 [New Zealand]
*Micromyzodium filicium* David, 1958	?	12		Kar et al. 1990 [India]
*Micromyzodium spinulosum* Miyazaki, 1971	?	10		Blackman 1986 [Japan]
*Micromyzus nikkoensis* Miyazaki, 1968	?	12		Blackman 1986 [Japan]
*Microsiphum woronieckae* Judenko, 1931	P(c)	12		Blackman and Eastop 2006 [?]
*Muscaphis escherichi irae* (Shaposhnikov, 1963)	P(c)	12		Kuznetsova 1968 (as *Toxopterella drepanosiphoides irae* Shaposhnikov, 1963) [St. Petersburg, Russia]
*Myzakkaia verbasci* (Chowdhuri, R.C. Basu, Chakrabarti & D.N. Raychaudhuri, 1969)	?	12		Kurl and Chauhan 1986a,1987a [Manali, Himachal Pradesh, India]
*Myzaphis bucktoni* (Jacob, 1946)	P(c)	13		Blackman and Eastop 2006 [one sample from Portugal]
*Myzaphis rosarum* (Kaltenbach, 1843)	P(c), P(o)	4		MacDonald and Harper 1965, Harper and MacDonald 1968 [Canada], Chen and Zhang 1985a [Beijing area, China] (cited after Blackman and Eastop 2015), Dutta and Gautam 1993 [Shimla, Himachal Pradesh, India], Reeta Devi and Gautam 2012 [Kullu region, Himachal Pradesh, India]
*Myzus cerasi* (Fabricius, 1775)	P(c)	10		Sun and Robinson 1966, Robinson and Chen 1969a [Canada], Kurl and Chauhan 1986d, 1987a [Solan, Himachal Pradesh, India], Bizzaro et al. 1999 [Italy], Blackman and Eastop 2015 [European and North American populations]
*Myzus cerasi umefoliae* (Shinji, 1924)	P(c)	12		Khuda-Bukhsh and Pal 1986a (as *Myzus cerasi*, but Blackman and Eastop 2015 - "possibly *Myzus umefoliae*") [Garhwal, Uttarakhand, India]
*Myzus dycei* Carver, 1961	?	12		Kurl and Chauhan 1986d,1987a [Solan, Himachal Pradesh, India], Kar et al. 1990 [India]
*Myzus fataunae* Shinji, 1924	P(c)	8		Blackman 1986 [Japan]
*Myzus formosanus* Takahashi, 1923	?	12		Pal and Khuda-Bukhsh 1980 [Sonprayag, Uttarakhand, India], Khuda-Bukhsh and Pal 1986a [Garhwal, Uttarakhand, India]
*Myzus hemerocallis* Takahashi, 1921	?	8		Chen and Zhang 1985a [Beijing area, China] (cited after Blackman and Eastop 2015)
		12		Blackman and Eastop 2006 [China; Kenya; Brazil]
*Myzus lythri* (Schrank, 1801)	P(c)	12		Kuznetsova and Shaposhnikov 1973 (as Myzus (Nevskia) lithri Schr.) [Crimea, Ukraine]
*Myzus mumecola* (Matsumura, 1917)	?	12		Chen and Zhang 1985a [Beijing area, China] (cited after Blackman and Eastop 2015), Khuda-Bukhsh and Pal 1986a [Garhwal, Uttarakhand, India]
*Myzus obtusirostris* S.K. David, Narayanan & Rajasingh, 1971	?P(c)	12		Kurl and Chauhan 1987a [Barog, Himachal Pradesh, India]
*Myzus ornatus* Laing, 1932	P(o), ?P(c)	12		Blackman 1980 [Great Britain], Dutta and Gautam 1993 [Shimla, Himachal Pradesh, India], Kapoor and Gautam 1994 [Himachal Pradesh, India]
*Myzus sorbi* Bhattacharya & Chakrabarti ex Maity, Bhattacharya & Chakrabarti, 1982	P(c)	12		Khuda-Bukhsh and Pal 1983a [Garhwal, Uttarakhand, India]
*Myzus varians* Davidson, 1912	P(c)	12		Blackman 1980 [Great Britain], Bizzaro et al. 1999 [Italy]
		13		Blackman 1980 [USA]
Myzus (Nectarosiphon) ajugae Schouteden, 1903	P(c)	12		Gut 1976 [Holland]
Myzus (Nectarosiphon) antirrhinii (Macchiati, 1883)	P(o), ?P(c)	11, 12, 13		Hales et al. 2000 [Australia]
		11, 12, 13, 14		Wilson et al. 2003 [Great Britain; France; Canada; Australia], Blackman and Eastop 2015 [?]
		13		Blackman and Spence 1996 (clone) [Great Britain], Hales 1993 [Australia]
		13, 14		Blackman 1987b [Europe; North America], Spence et al. 1998, Terradot et al. 1999 [Great Britain]
Myzus (Nectarosiphon) asteriae Shinji, 1941	?	12		Blackman 1986 [Japan]
Myzus (Nectarosiphon) certus (Walker, 1843)	P(c), P(o)	12		Gut 1976 [Holland], Blackman 1987b [?], Spence et al. 1998 [Great Britain, USA]
Myzus (Nectarosiphon) dianthicola Hille Ris Lambers, 1966	P(o)	14 (heterozy-gous)		Blackman 1980 [Great Britain; New Zealand], Blackman 1987b [?]
Myzus (Nectarosiphon) icelandicus Blackman, 1986	P(c)	10		Blackman and Eastop 2015 [?]
Myzus (Nectarosiphon) ligustri (Mosley, 1841)	P(c)	12		Blackman 1980 [Great Britain]
Myzus (Nectarosiphon) myosotidis (Börner, 1950)	P(c)	12		Gut 1976 [Holland]
Myzus (Nectarosiphon) persicae persicae (Sulzer, 1776)	P(c), P(o)	8		Chattopadhyay and Raychaudhuri 1980 [Kolkata, West Bengal, India]
		8, 12, 13		Raychaudhuri and Das 1987 [India]
		10, 11,12		Misra and Kurl 1983 [Jodhpur, Rajasthan, India]
		10, 11,12, 13		Khuda-Bukhsh 1980 (as *Macrosiphum*) [Garhwal, Uttarakhand, India], Kurl 1986 [Meghalaya, India]
		11, 12		Hales 1993 [Australia], Spence and Blackman 2000 [clon]
		11, 12, 13		Spence et al. 1998 [France; Great Britain; lab. cultures]
		11, 12, 18 (triploid)		Yang and Zhang 2000, Yang et al. 2000 [China]
		12/11	XX/X0	Hales and Mittler 1983, 1987 [Australia], Searle and Mittler 1991 [Washington, USA]
		12		Shinji 1941b [Japan], Colling 1955 [Great Britain], Dionne and Spicer 1957 (as *Myzus solanifolii*), Sun and Robinson 1966, Robinson and Chen 1969a [Canada], Kuznetsova 1969 (as *Myzodes*) [Alma-Ata, Kazakhstan], Kuznetsova and Shaposhnikov 1973 (as *Myzodes*) [St. Petersburg, Russia], Kulkarni and Kacker 1980,1981b [India], Chen and Zhang 1985a [Beijing area, China] (cited after Blackman and Eastop 2015), Blackman 1986 [Japan] (based on 2n (♂) =6 (Shinji 1941b)), Khuda-Bukhsh and Pal 1986a [Garhwal, Uttarakhand, India], Blackman 1987b [?], Kar et al. 1990 [India], Gautam and Sharma 1990 [Himachal Pradesh, India], Dutta and Gautam 1993 [Shimla, Himachal Pradesh, India], Blackman and Spence 1996 [Great Britain], Spence and Blackman 1998 [Great Britain], Terradot et al. 1999 [Great Britain; France; Spain; Cuba], Wilson et al. 2002 [Australia], Gautam and Dhatwalia 2003 [Solan, Himachal Pradesh, India], Samkaria et al. 2010 [Palampur, Himachal Pradesh, India], Jangra et al. 2014 [Jammu and Kashmir, India]
		12, 13		Blackman and Takada 1976, 1977, Blackman 1986 [Japan],
		12, 14		Sethi and Nagaich 1972 [Shimla, Himachal Pradesh, India]
		12, 13, 14		Blackman 1971 (as *Myzodes*) [Great Britain; France], Blackman, Brown and Eastop 1987 [Europe; Japan; USA], Monti et al. 2012a [Italy; Great Britain], Rivi et al. 2012 [Italy]
		14		Cognetti 1961a, b (as *Myzodes*), Cognetti and Cognetti-Varriale 1961 [Italy]
		12, 13, 14, 15, 16, 17		Monti et al. 2012b [clone from Hertfordshire, Great Britain]
		12/11, 11, 12, 13, 14, 18 (triploid)	XX/X0	Blackman 1980 [Europe; Japan; USA; Chile; New Zealand]
		12		Blackman 1987b [North America], Harlow et al. 1991 [North Carolina, USA], Dutta and Gautam 1993 [Shimla, Himachal Pradesh, India], Terradot et al. 1999 [France]
Myzus (Nectarosiphon) persicae nicotianae Blackman, 1987	P(c), P(o)	18 (triploid)		Takada et al. 1978 [Japan]
Myzus (Sciamyzus) ascalonicus Doncaster, 1946	P(o)	12		Gut 1976 [Holland], Blackman 1987b [?], Kapoor and Gautam 1994 [Shimla, Himachal Pradesh, India], Blackman and Spence 1996 [Great Britain], Gautam and Dhatwalia 2003 [Solan, Himachal Pradesh, India]
Myzus (Sciamyzus) cymbalariae Stroyan, 1954	P(o), ?P(c)	12		Blackman 1980 [Great Britain], Blackman 1987b [?]
*Myzus* sp. 1	?	10		Khuda-Bukhsh and Pal 1986a [Garhwal, Uttarakhand, India]
*Myzus* sp. 2	?	12		Khuda-Bukhsh and Kar 1990 [Kalimpong, West Bengal, India]
*Myzus* sp. 3	?	12		Kar et al. 1990 [India]
*Nasonovia compositellae nigra* (Hille Ris Lambers, 1931)	P(o), P(c)	11 (he-terozygous)		Blackman 1980 (as *Nasonovia nigra* (Hille Ris Lambers, 1931)) [Great Britain], Blackman and Eastop 2006 [Great Britain] (one sample)
*Nasonovia jammuensis* Verma, 1966	?	12		Dutta and Gautam 1993 [Shimla, Himachal Pradesh, India]
*Nasonovia ribisnigri* (Mosley, 1841)	P(c)	12		Blackman 1980 [Great Britain]
		14		Shinji 1941a (as *Amphorophora ribicola*) [Japan]
*Nasonovia rostrata* David & Hameed, 1974	P(c)	12		Pal and Khuda-Bukhsh 1980, Khuda-Bukhsh and Pal 1986b [Triyuginarayan, Uttarakhand, India]
Nasonovia (Kakimia) alpina (Gillette & Palmer, 1928)	?	10		Blackman and Eastop 2015 [?]
Nasonovia (Kakimia) aquilegiae (Essig, 1917)	P(c)	10		Sun and Robinson 1966, Robinson and Chen 1969a (as *Kakimia essigi* (Gillette & Palmer, 1928)) [Canada]
Nasonovia (Kakimia) cynosbati (Oestlund, 1887)	P(c)	10		Sun and Robinson 1966, Robinson and Chen 1969a (as *Kakimia* and as *Kakimia thomasi* (Hottes & Frison 1931)) [Canada]
Nasonovia (Kakimia) dasyphylli Stroyan, 1957	P(c), P(o)	12 (heterozy-gous)		Blackman and Eastop 2006 [Great Britain](one sample)
*Nearctaphis bakeri* (Cowen, 1895)	P(c), P(o)	12		Blackman 1980 [Great Britain], Dutta and Gautam 1993 [Shimla, Himachal Pradesh, India]
*Nearctaphis californica* Hille Ris Lambers, 1970	?P(c)	12		Blackman and Eastop 1994 [?]
*Neoceruraphis viburnicola* (Gillette, 1909)	P(c)	14		Sun and Robinson 1966, Robinson and Chen 1969a [Canada]
*Neomyzus circumflexus* (Buckton, 1876)	P(o)	8		Blackman 1980 [Great Britain], Dutta and Gautam 1993 [Shimla, Himachal Pradesh, India], Gautam and Dhatwalia 2003 [Hamirpur, Himachal Pradesh, India]
		10		Kar and Khuda-Bukhsh 1986, Khuda-Bukhsh and Kar 1990 [Kalimpong, West Bengal, India]
*Neomyzus parthenocissi* (Takahashi, 1965)	?	12		Blackman 1986 (as Aulacorthum (Neomyzus)) [Japan]
*Neomyzus parthenocissi* (Takahashi, 1965)	?	12		Blackman 1986 (as Aulacorthum (Neomyzus)) [Japan]
*Neotoxoptera formosana* (Takahashi, 1921)	P(o)	12		Blackman and Eastop 1984 [?]
*Neotoxoptera violae* (Pergande, 1900)	?	12		Blackman and Eastop 1984 (as *Neotoxoptera oliveri* (Essig, 1935)) [?]
*Obtusicauda coweni* (Hunter, 1901)	P(c)	12		Blackman and Eastop 2006 [?]
*Oedisiphum soureni* A.N. Basu, 1964	?	8		Kurl and Chauhan 1986c [Barog, Himachal Pradesh, India]
*Ovatomyzus boraginacearum* Eastop, 1952	P(o)	12		Gut 1976 [Holland]
*Ovatomyzus stachyos* Hille Ris Lambers, 1947	?P(o)	12		Blackman and Eastop 2006 [?]
*Ovatus crataegarius* (Walker, 1950)	P(c), P(o)	12		Shinji 1941a (as *Phorodon menthae*) [Japan], Blackman and Eastop 1984 [?], Chen and Zhang 1985a [Beijing area, China] (cited after Blackman and Eastop 2015), Blackman 1986 [Japan] (based on 2n (♂) =6 (Shinji 1941a)), Gautam and Dhatwalia 2003 [Shimla, Himachal Pradesh, India]
*Ovatus insitus* (Walker, 1849)	P(c)	12/11	XX/X0	Kuznetsova and Shaposhnikov 1973 [St.Petersburg, Russia]
*Ovatus malisuctus* (Matsumura, 1918)	P(c)	12		Chen and Zhang 1985a (as *Myzus*) [Beijing area, China] (cited after Blackman and Eastop 2015)
*Paczoskia obtecta* Börner, 1950	?	12		Blackman 1980 [Sweden]
*Paradoxaphis aristoteliae* Sunde, 1988	P(c)	8		Blackman and Eastop 2015 [?]
*Paramyzus longirostris* Miyazaki, 1971	?	14		Blackman 1986 [Japan]
*Pentalonia kalimpongensis* (A.N. Basu, (1967) 1968)	P(c)	12		Khuda-Bukhsh and Kar 1990 [Kalimpong, West Bengal, India]
*Pentalonia nigronervosa* Coquerel, 1859	P(o), ?P(c)	14		Blackman and Eastop 1984 [?], Panigrahy and Patnaik 1987 [Chatrapur, Odisha, India], Khuda-Bukhsh and Kar 1990 [Kalyani, West Bengal, India]
*Phorodon cannabis* Passerini, 1860	P(c)	12		Pal and Khuda-Bukhsh 1980, Khuda-Bukhsh and Pal 1986b [Triyuginarayan, Uttarakhand, India], Dutta and Gautam 1993 [Shimla, Himachal Pradesh, India]
*Pentalonia humuli humuli* (Schrank, 1801)	P(c)	12		Kuznetsova and Shaposhnikov 1973 (as *Phorodon pruni* Geoffr.) [Crimea, Ukraine], Blackman 1980 [Great Britain]
*Pentalonia humuli japonensis* Takahashi, 1965	P(c)	12		Shinji 1941a [Japan], Blackman and Eastop 1984 [?], Chen and Zhang 1985a [Beijing area, China] (cited after Blackman and Eastop 2015), Blackman 1986 [Japan] (their own data and based on n(♂) = 6 (Shinji 1941a))
*Pentalonia humulifoliae* Tseng & Tao, 1938	?P(c)	12		Chen and Zhang 1985b (cited after Blackman and Eastop 2015)
*Pleotrichophorus duponti* Hille Ris Lambers, 1935	P(c)	14		Blackman 1980 [Great Britain]
*Pleotrichophorus glandulosus* (Kaltenbach, 1846)	P(c)	14		Blackman 1980 [Great Britain]
*Plocamaphis flocculosa* (Weed, 1891)	P(c)	30–34?		Kuznetsova and Shaposhnikov 1973 [St.Petersburg, Russia]
*Protaphis knowltoni* (Hottes & Frison, 1931)	P(c)	8		Sun and Robinson 1966, Robinson and Chen 1969a (as *Aphis*) [Canada]
*Protaphis middletonii* (Thomas, 1879)	P(c), ?P(o)	8		Blackman 1980 (as *Aphis armoraciae* Cowen, 1895) [USA]
		8, 9		Blackman 1980 (as Aphis (Protaphis) maidiradicis Forbes, 1891) [USA]
*Protaphis pseudocardui* (Theobald, 1915)	?	8		Blackman and Eastop 2015 [?]
*Protaphis terricola* (Rondani, 1847)	P(c)	8		Blackman 1980 (as Aphis (Protaphis)) [Spain]
*Protaphis* sp.	?	8, 9		Blackman 1980 (as Aphis (Protaphis)) [Iran] (from *Artemisia dracunculus*)
*Pseudocercidis rosae* Richards, 1961	P(c)	12		Robinson and Chen 1969a [Canada]
*Pseudomegoura magnoliae* (Essig & Kuwana, 1918)	P(o), P(c)	12		Blackman 1986 [Japan] (their own data ex. cult. on potato and based on n(♂) = 6 (Shinji 1927))
*Pterocomma bicolor* (Oestlund, 1887)	P(c)	8		Robinson and Chen 1969a [Canada]
*Pterocomma jacksoni* Theobald, 1921	?	30–34?		Kuznetsova and Shaposhnikov 1973 [St.Petersburg, Russia]
*Pterocomma konoi* Hori, 1939	P(c)	8		Blackman 1986 [Japan]
*Pterocomma pilosum* Buckton, 1879	P(c)	8		Kuznetsova and Shaposhnikov 1973 [St.Petersburg, Russia]
*Pterocomma populeum* (Kaltenbach, 1843)	P(c)	8		Kuznetsova and Shaposhnikov 1973 [St.Petersburg, Russia]
*Pterocomma rufipes* (Hartig, 1841)	P(c)	8		Kuznetsova and Shaposhnikov 1973 (as *Pterocomma steinheili* Mordvilko, 1901) [St.Petersburg, Russia]
		8, 9		Kuznetsova 1974 (as *steinheili* Mordv.) [?]
*Pterocomma salicis* (Linnaeus, 1758)	P(c)	6		Tannreuther 1907 [USA]
		30-34?		Kuznetsova and Shaposhnikov 1973 [St.Petersburg, Russia]
		58		Blackman 1980 [Great Britain], Blackman and Eastop 1994 [?]
*Pterocomma salijaponica* (Shinji, 1924)	?	8/7	XX/X0	Shinji 1931 (as *Melanoxantherium*) [Japan], Blackman 1986 (as *Plocamaphis*) [Japan] (based on n(♂) = 4 (Shinji 1931))
		22		Shinji 1927, 1941a (as *Melanoxantherium*), Blackman 1986 (as *Plocamaphis*) [Japan] (based on n(♂) = 11 (Shinji 1927, 1941))
*Pterocomma sanguiceps* Richards, 1967	?	8		Blackman and Eastop 1994 [?]
*Pterocomma smithiae* (Monell, 1879)	P(c)	8		Sun and Robinson 1966, Robinson and Chen 1969a [Canada]
*Pterocomma tremulae* Börner, 1940	?	8		Kuznetsova and Shaposhnikov 1973 [St.Petersburg, Russia]
*Pterocomma yezoense* (Hori, 1929)	P(c)	8		Blackman and Eastop 1994 [?]
*Rhodobium porosum* (Sanderson, 1900)	P(c), P(o)	14		Kar et al. 1990 [India]
Rhopalomyzus (Judenkoa) lonicerae (Siebold, 1839)	P(c)	12		Sun and Robinson 1966, Robinson and Chen 1969a [Canada]
*Rhopalosiphoninus hydrangeae* (Matsumura, 1918)	P(c)	12		Shinji 1941a [Japan]
*Rhopalosiphoninus latysiphon* (Davidson, 1912)	P(o), ?P(c)	6 (+1)		Gut 1976 [Holland]
*Rhopalosiphoninus tiliae* (Matsumura, 1918)	P(c)	12		Shinji 1941a (as *Rhopalosiphoninus adenocauli*), 1941b (as *Rhopalosiphoninus nobukii*), Blackman 1986 [Japan] (based on n(♂) = 6 (Shinji 1941b)]
Rhopalosiphoninus (Neorhopalosiphoninus) staphyleae (Koch, 1854)	P(c), P(o)	10		Blackman and Eastop 2015 [?]
*Rhopalosiphum cerasifoliae* (Fitch, 1855)	P(c)	8		Robinson and Chen 1969a [Canada]
*Rhopalosiphum enigmae* Hottes & Frison, 1931	P(c)	10		Blackman and Eastop 2006 [?]
*Rhopalosiphum maidis* (Fitch, 1856)	P(o), ?P(c)	8		Sun and Robinson 1966, Robinson and Chen 1969a [Canada], Mayo and Starks 1972 [USA], Kurl 1978 [Jodhpur, Rajasthan, India], Kar and Khuda-Bukhsh 1989 [Kalimpong, West Bengal, India], Dutta and Gautam 1993, Gautam and Dhatwalia 2003 [Shimla, Himachal Pradesh, India], Samkaria et al. 2010 [Palampur, Himachal Pradesh, India]
		8, 9		Blackman 1980, Hales and Cowen 1990 [Australia], Kuznetsova and Gandrabur 1991 [Fergana, Uzbekistan], De Barro 1992 [Australia]
		8,10		Chattopadhyay et al. 1982 [India], Panigrahy and Patnaik 1991 [Chatrapur, Odisha, India]
		8, 9, 10		Blackman et al. 1990, Blackman and Brown 1991 [USA], Jauset et al. 2000 [Catalonia, Spain]
		8, 9, 10, 11		Blackman, Brown and Eastop 1987 [Europe; North America; Iran; Israel; Australia (Tasmania)], Brown and Blackman 1988 [all continents except Antarctica] ("there is an association between karyotype and host plant, the barley-colonizing form in the northern hemisphere having 2n = 10, whereas populations on maize and sorghum have 2n = 8")
		9, 10, 11		Kuznetsova and Gandrabur 1991 [St. Petersburg, Russia]
*Rhopalosiphum nymphaeae* (Linnaeus, 1761)	P(c)	8		Kuznetsova and Shaposhnikov 1973 [Tbilisi, Georgia], Blackman and Eastop 2006 [Italy]
		16		Behura and Bohidar 1978 [India] (cited after Blackman and Eastop 2015), Kurl 1978 [Meerut, Uttar Pradesh, India],
		16, 17		Kurl 1986 [Meghalaya, India]
*Rhopalosiphum oxyacanthae* (Schrank, 1801)	P(c)	10		Sun and Robinson 1966, Robinson and Chen 1969a, b (as *Rhopalosiphum fitchii* (Sanderson, 1902)) [Canada], Kuznetsova and Shaposhnikov 1973 (as *Rhopalosiphum insertum* Walk.) [St.Petersburg, Russia], Kuznetsova et al. 1988 (as *Rhopalosiphum insertum* Walk.) [St. Petersburg, Russia], Hales and Cowen 1990 (as *Rhopalosiphum insertum* Walk.) [Australia]
*Rhopalosiphum padi* (Linnaeus, 1758)	P(c), P(o)	8/7	XX/X0	Fox 1957 [Pennsylvania, USA]
		8		Sun and Robinson 1966, Robinson and Chen 1969a, b [Canada], Mayo and Starks 1972 [USA], Chen and Zhang 1985a [Beijing area, China] (cited after Blackman and Eastop 2015), Kurl and Misra 1979 [Rajasthan, India], Kar and Khuda-Bukhsh 1989 [Kalimpong, West Bengal, India], Kuznetsova and Gandrabur 1991 [St.Petersburg, Russia], De Barro 1992 [Australia], Valenzuela et al. 2009 [Victoria, Australia], Monti et al. 2010 [Italy]
		8, 9		Hales and Cowen 1990 [Australia]
*Rhopalosiphum padiformis* Richards, 1962	?P(c)	10		Blackman and Eastop 1984 [?]
*Rhopalosiphum rufiabdominale* (Sasaki, 1899)	P(c), P(o)	8		Gut 1976 [Holland], Khuda-Bukhsh and Kar 1990 [Kalyani, West Bengal, India], Hales and Cowen 1990 [Australia]
*Rhopalosiphum rufulum* Richards, 1960	P(c), ?P(o)	8		Gut 1976 [Holland]
*Rhopalosiphum* sp.	?	8		Bulman et al. 2004 [New Zealand]
*Rhopalosiphum* sp. ["undescribed species"]	?	9		Hales and Cowen 1990 (similar to *Rhopalosiphum padi*) [Australia], Valenzuela et al. 2009 [Victoria, Australia]
*Rhopalosiphum* sp.["near *insertum*"]	?	10		Valenzuela et al. 2009 [Victoria, Australia]
*Roepkea marchali* (Börner, 1931)	P(c)	12		Kuznetsova 1968 [Georgia]
*Sappaphis piri* Matsumura, 1918	P(c)	12		Kuznetsova 1968 [Vladivostok, Russia]
*Sappaphis sinipiricola* G. Zhang, 1980	?	12		Chen and Zhang 1985a [Beijing area, China] (cited after Blackman and Eastop 2015)
*Schizaphis graminum* (Rondani, 1847(1852))	P(c), P(o)	6, 8		Rubín de Celis et al. 1997 [Brazil]
		7, 8, 12		Mayo and Starks 1972 [USA]
		8		Sun and Robinson 1966, Robinson and Chen 1969a [Canada], Kuznetsova and Gandrabur 1991 [Ukraine]
		8	XX/X0	Mandrioli et al. 1999 [Modena, Italia]
*Schizaphis mali* Shaposhnikov, 1979	P(c)	8		Blackman and Eastop 2006 [?]
*Schizaphis piricola* (Matsumura, 1917)	P(c)	8		Chen and Zhang 1985a [Beijing area, China] (cited after Blackman and Eastop 2015)
*Schizaphis rotundiventris* (Signoret, 1860)	P(c), P(o)	8		Blackman and Eastop 2006 [?]
Schizaphis (Paraschizaphis) acori (Shinji)	P(c)	8		Blackman and Eastop 2006 [?]
Schizaphis (Paraschizaphis) rosazevedoi (Ilharco, 1961)	P(o)	8		Blackman and Eastop 2006 [?]
Schizaphis (Paraschizaphis) scirpi (Passerini, 1874)	P(c)	8		Gut 1976 (as *Paraschizaphis*) [Holland]
*Semiaphis heraclei* (Takahashi, 1921)	P(c)	8		Blackman and Eastop 1984 [?],Chen and Zhang 1985a [Beijing area, China] (cited after Blackman and Eastop 2015), Blackman 1986 [Japan], Gautam and Kapoor 2002 [Una, Himachal Pradesh, India]
		10		Pal and Khuda-Bukhsh 1983 [Garhwal, Uttarakhand, India]
*Shinjia orientalis* (Mordvilko, 1929)	P(c), ?P(o)	12		Shinji 1941b (as *Microtarsus pterydifoliae*), Blackman 1986 [Japan] (based on n(♂) = 6 (Shinji 1941b)), Blackman and Eastop 2006 [?]
*Sinomegoura citricola* (van der Goot, 1917)	P(o)	12		Kulkarni 1984 [Darjeeling, West Bengal, India], Dutta and Gautam 1993 [Shimla, Himachal Pradesh, India]
		16		Chen and Zhang 1985b (cited after Blackman and Eastop 2015)
		18		Kar and Khuda-Bukhsh 1986, Khuda-Bukhsh and Kar 1990 [Kalimpong, West Bengal, India]
*Sinomegoura photiniae* (Takahashi, 1936)	?	18		Khuda-Bukhsh and Kar 1990 [Kalyani, West Bengal, India]
*Sinomegoura pyri* A.K. Ghosh & D.N. Raychaudhuri, 1968	?	8		Kar et al. 1990 [India]
*Sinomegoura rhododendri* (Takahashi, 1937)	?	18		Gautam and Kumar 2006 [Shimla, Himachal Pradesh, India]
*Sitobion alopecuri* (Takahashi, 1921)	P(c)	18		Blackman and Eastop 2015 [British Columbia]
*Sitobion aulacorthoides* (David, Narayanan & Rajasingh, (1970) 1971)	?	18		Blackman and Eastop 2015 [?]
*Sitobion avenae* (Fabricius, 1775)	P(c), P(o)	18		Sun and Robinson 1966, Robinson and Chen 1969a (as *Macrosiphum*) [Canada], Kuznetsova and Shaposhnikov 1973 [Crimea, Ukraine], Kuznetsova and Gandrabur 1991 [St.Petersburg, Russia], Rubín de Celis et al. 1997 [Brazil]
*Sitobion fragariae* (Walker, 1848)	P(c)	18		Kuznetsova and Shaposhnikov 1973 [Crimea, Ukraine], Gautam and Kapoor 2002 [Una, Himachal Pradesh, India]
*Sitobion graminis* Takahashi, 1950	?P(o)	18		Kurl and Chauhan 1986a (as *Macrosiphum*) [Jwalaji, Himachal Pradesh, India]
*Sitobion gravelii* (van der Goot, 1917)	?P(c)	12		Khuda-Bukhsh and Basu 1987 (as *Sitobion spinotibium* on *Artemisia vulgaris*) (cited after Blackman and Eastop 2015)
*Sitobion ibarae* (Matsumura, 1917)	?P(o)	14/13		Shinji 1941a (as *Macrosiphum*) [Japan]
*Sitobion indicum* A.N. Basu, 1964	P(o)	17, 18		Kurl 1986 (as *Macrosiphum*) [Meghalaya, India]
		18		Kurl 1980b (as *Macrosiphum*) [Meghalaya, India]
*Sitobion luteum* (Buckton, 1876)	P(o)	12		Gut 1976 [Holland]
*Sitobion miscanthi* (Takahashi, 1921)	P(o), ?P(c)	14		Kurl and Chauhan 1986a (as *Macrosiphum*) [Solan, Himachal Pradesh, India]
		18		Kurl and Chauhan 1987a (as *Macrosiphum*) [Solan, Himachal Pradesh, India], Dutta and Gautam 1993 [Shimla, Himachal Pradesh, India]
		17, 18		Turak and Hales 1990 [Australia]
		17, 18, 20		Hales et al. 1990, Sunnucks and Hales 1996, Hales et al. 2010 [Australia]
		17, 18, 20, 21		Sunnucks et al. 1996, Hales et al. 1998 [Australia]
		17, 18, 20, 22		Wilson et al. 1999 [New Zealand]
*Sitobion nigrinectarium* (Theobald, 1915)	?	18		Blackman 1980 [Kenya]
*Sitobion ochnearum* (Eastop, 1959)	?	18		Blackman and Eastop 2006 [?]
*Sitobion pseudoluteum* A.K. Ghosh, 1969	?	18		Kar et al. 1990 [India]
*Sitobion rosaeiformis* (Das, 1918)	P(c)	14, 18		Gautam and Dutta 1994 [Shimla, Himachal Pradesh, India]
		16, 17,18		Kurl 1986 (as Macrosiphum (Sitobion)) [Meghalaya, India]
		18		Khuda-Bukhsh 1980 (as *Macrosiphum*) [Garhwal, Uttarakhand, India], Kulkarni and Kacker 1981a [Kursiong, West Bengal, India], Kurl and Misra 1983 (as Macrosiphum (Sitobion) rosaeformis) [Jodhpur, Rajasthan, India], Raychaudhuri and Das 1987 [India], Kar and Khuda-Bukhsh 1989 [Shillong, Meghalaya, India], Kar et al. 1990 [India]
*Sitobion rosivorum* (G. Zhang, 1980)	?	18		Chen and Zhang 1985a (as *Macrosiphum*) [Beijing area, China] (cited after Blackman and Eastop 2015)
*Sitobion takahashii* (Eastop, 1959)	?	18		Khuda-Bukhsh and Kar 1989a (cited after Blackman and Eastop 2015)
*Sitobion wikstroemiae* (Mamet, 1939)	?	16		Blackman 1980 [Kenya]
Sitobion sp. prope avenae (Fabricius, 1775)	?	12		Kapoor Gautam 1994 [Shimla, Himachal Pradesh, India]
*Sitobion luteum* (Buckton, 1876)	P(o)	12		Gut 1976 [Holland]
*Sitobion miscanthi* (Takahashi, 1921)	P(o), ?P(c)	14		Kurl and Chauhan 1986a (as *Macrosiphum*) [Solan, Himachal Pradesh, India]
		18		Kurl and Chauhan 1987a (as *Macrosiphum*) [Solan, Himachal Pradesh, India], Dutta and Gautam 1993 [Shimla, Himachal Pradesh, India]
		17, 18		Turak and Hales 1990 [Australia]
		17, 18, 20		Hales et al. 1990, Sunnucks and Hales 1996, Hales et al. 2010 [Australia]
		17, 18, 20, 21		Sunnucks et al. 1996, Hales et al. 1998 [Australia]
		17, 18, 20, 22		Wilson et al. 1999 [New Zealand]
*Sitobion nigrinectarium* (Theobald, 1915)	?	18		Blackman 1980 [Kenya]
*Sitobion ochnearum* (Eastop, 1959)	?	18		Blackman and Eastop 2006 [?]
*Sitobion pseudoluteum* A.K. Ghosh, 1969	?	18		Kar et al. 1990 [India]
*Sitobion rosaeiformis* (Das, 1918)	P(c)	14, 18		Gautam and Dutta 1994 [Shimla, Himachal Pradesh, India]
		16, 17,18		Kurl 1986 (as Macrosiphum (Sitobion)) [Meghalaya, India]
		18		Khuda-Bukhsh 1980 (as *Macrosiphum*) [Garhwal, Uttarakhand, India], Kulkarni and Kacker 1981a [Kursiong, West Bengal, India], Kurl and Misra 1983 (as Macrosiphum (Sitobion) rosaeformis) [Jodhpur, Rajasthan, India], Raychaudhuri and Das 1987 [India], Kar and Khuda-Bukhsh 1989 [Shillong, Meghalaya, India], Kar et al. 1990 [India]
*Sitobion rosivorum* (G. Zhang, 1980)	?	18		Chen and Zhang 1985a (as *Macrosiphum*) [Beijing area, China] (cited after Blackman and Eastop 2015)
*Sitobion takahashii* (Eastop, 1959)	?	18		Khuda-Bukhsh and Kar 1989a (cited after Blackman and Eastop 2015)
*Sitobion wikstroemiae* (Mamet, 1939)	?	16		Blackman 1980 [Kenya]
Sitobion sp. prope avenae (Fabricius, 1775)	?	12		Kapoor and Gautam 1994 [Shimla, Himachal Pradesh, India]
Sitobion sp. prope fragariae (Walker, 1848)	?	18		Turak and Hales 1990, Hales et al. 1990, De Barro 1992, Sunnucks et al. 1996, Hales et al. 1998 [Australia], Wilson et al. 1999 [New Zealand]
Sitobion sp. prope rosaeiformis (Das, 1918)	?	18		Dutta and Gautam 1993 [Shimla, Himachal Pradesh, India]
*Sitobion* sp.	?	12		Dutta and Gautam 1993 [Shimla, Himachal Pradesh, India]
*Sorbaphis chaetosiphon* Shaposhnikov, 1950	P(c)	38		Blackman 1986 [Japan]
*Staticobium limonii* (Contarini, 1847)	?	12		Blackman and Eastop 2006 [?]
*Titanosiphon neoartemisiae* (Takahashi, 1921)	P(c), P(o)	8		Blackman and Eastop 2006 [for specimens on *Staticobium dracunculus* in Iran]
*Tricaudatus polygoni* (Narzikulov, 1953)	P(c)	8		Kar et al. 1990 [India]
Trichosiphonaphis (Xenomyzus) polygoni (van der Goot, 1917)	?	12		Chen and Zhang 1985a (as *Trichosiphonaphis ishimikawae* (Shinji 1941)) (cited after Blackman and Eastop 2015)
Trichosiphonaphis (Xenomyzus) polygonifoliae (Shinji, 1944)	P(c)	12		Blackman and Eastop 2015 [?]
Trichosiphonaphis (Xenomyzus) tade (Shinji, 1927)	?	10/9	XX/X0	Shinji 1927, 1931, 1941a (as *Carolinaia*), Blackman 1986 [Japan] (based on n(♂) = 5 (Shinji 1927, 1931))
		12		Blackman 1986 [Japan]
*Tubaphis clematophila* (Takahashi, 1965)	?	12		Blackman 1986 [Japan]
Tuberocephalus (Trichosiphoniella) higansakurae (Monzen, 1927)	P(c)	12		Blackman 1986 [Japan]
Trichosiphonaphis (Trichosiphoniella) liaoningensis G. Zhang & Zhong, 1976	P(c)	12		Chen and Zhang 1985a [Beijing area, China] (cited after Blackman and Eastop 2015)
Trichosiphonaphis (Trichosiphoniella) misakurae Moritsu & Hamasaki, 1983	P(c)	12		Blackman and Eastop 1994 [?]
Trichosiphonaphis (Trichosiphoniella) momonis (Matsumura, 1917)	P(c)	12		Chen and Zhang 1985a [Beijing area, China] (cited after Blackman and Eastop 2015)
*Uroleucon achilleae* (Koch, 1855)	P(c)	12		Blackman 1980 [Great Britain]
*Uroleucon ambrosiae* (Thomas, 1878)	P(c), P(o)	12		Olive 1967 (as *Dactynotus*) [USA], Robinson and Chen 1969a (as *Dactynotus*) [Canada]
*Uroleucon chondrillae* (Nevsky, 1929)	P(c)	12		Blackman and Eastop 2006 [?]
*Uroleucon chrysopsidicola* (Olive, 1963)	P(c)	12		Olive 1967 (as *Dactynotus*) [USA]
*Uroleucon cirsii* (Linnaeus, 1758)	P(c)	10		Sun and Robinson 1966, Robinson and Chen 1969a (as *Dactynotus*) [Canada], Kuznetsova and Gandrabur 1991 [St.Petersburg, Russia]
*Uroleucon formosanum* (Takahashi, 1921)	?	12	XX/X0	Shinji 1927, 1931 (as *Macrosiphum sonchi*), Blackman 1986 [Japan] (based on n(♂) = 6 (Shinji 1927, 1931))
*Uroleucon fuscaudatum* Chakrabarti & D.N. Raychaudhuri, 1978	?	12		Pal and Khuda-Bukhsh 1980, Khuda-Bukhsh and Pal 1986b [Rambara, Uttarakhand, India]
*Uroleucon cichorii grossum* (Hille Ris Lambers, 1939)	P(c)	12		Gut 1976 [Holland]
*Uroleucon hypochoeridis* (Fabricius, 1779)	P(c)	12		Gut 1976 [Holland]
*Uroleucon jaceicola* (Hille Ris Lambers, 1939)	P(c)	12		Gut 1976 [Holland]
*Uroleucon longisetosum* Chakrabarti & Verma, 1975	P(c)	10		Pal and Khuda-Bukhsh 1980, Khuda-Bukhsh and Pal 1986b [Gobindoghat, Uttarakhand, India]
*Uroleucon macolai* (Blanchard, 1932)	P(c)	12		Blackman and Eastop 2006 [?]
*Uroleucon nigrotuberculatum* (Olive, 1963)	P(c)	12		Olive 1967 (as *Dactynotus*) [USA]
*Uroleucon paucosensoriatum* (Hille Ris Lambers, 1960)	P(c)	12		Robinson and Chen 1969a (as *Dactynotus*) [Canada]
*Uroleucon pseudambrosiae* (Olive, 1963)	?	12		Olive 1967 (as *Dactynotus*) [USA]
*Uroleucon pseudotanaceti* (Verma, 1969 (1970))	P(c)	12		Kurl and Chauhan 1986a,1987a [Kangra, Himachal Pradesh, India]
*Uroleucon reynoldense* (Olive, 1965)	?	12		Olive 1967 (as *Dactynotus*) [USA]
*Uroleucon rudbeckiae* (Fitch, 1851)	P(c)	12		Olive 1967 (as *Dactynotus*) [USA]
*Uroleucon russellae* (Hille Ris Lambers, 1960)	P(c)	12		Olive 1967 (as *Dactynotus*) [USA]
*Uroleucon simlaense* Chakrabarti, A.K. Ghosh & D.N. Raychaudhuri, 1971	?	12		Kurl and Chauhan 1987a [Kandaghat, Himachal Pradesh, India]
*Uroleucon sonchellum* (Monell, 1879)	P(c)	12		Olive 1967 (as *Dactynotus*) [USA]
*Uroleucon sonchi* (Linnaeus, 1767)	P(c), ?P(o)	12	XX/X0	Olive 1967 (as *Dactynotus*) [USA]
		12		Kulkarni and Kacker 1981a [Dadhau, Himachal Pradesh, India], Kurl and Chauhan 1986c, 1987a [Barog, Himachal Pradesh, India], Kar et al. 1990 [India], Dutta and Gautam 1993 [Shimla, Himachal Pradesh, India]
*Uroleucon tanaceti* (Linnaeus, 1758)	P(c)	12		Gut 1976 [Holland]
*Uroleucon tussilaginis* (Walker, 1850)	P(c)	8?		Kuznetsova 1974 (as *Dactynotus basalis* Walker?) [?] (Blackman and Eastop 2015 supposed that the karyotype in Kuznetsova 1974 illustrated resembles that of *Acyrthosiphon pisum*)
Uroleucon (Belochilum) inulae (Ferrari, 1872)	?	12		Blackman and Eastop 2006 [?]
Uroleucon (Lambersius) anomalae (Hottes & Frison, 1931)	?	12		Olive 1967 (as *Dactynotus*) [USA]
Uroleucon (Lambersius) bradburyi (Olive, 1965)	P(c)	12		Olive 1967 (as *Dactynotus*) [USA]
Uroleucon (Lambersius) erigeronense (Thomas, 1878)	P(c)	12		Blackman and Eastop 2015 [?]
Uroleucon (Lambersius) gravicorne (Patch, 1919)	P(c)	12		Olive 1967 (as *Dactynotus*) [USA]
Uroleucon (Lambersius) luteolum (Williams, 1911)	P(c)	12		Olive 1967 (as *Dactynotus tissoti* Boudreaux, 1948 (1949)) [USA]
Uroleucon (Lambersius) penderum Robinson, 1986	?	12		Blackman and Eastop 2006 [British Columbia, Canada]
Uroleucon (Lambersius) richardsi Robinson, 1964	P(c)	12		Robinson and Chen 1969a (as *Dactynotus*) [Canada]
Uroleucon (Uromelan) carthami (Hille Ris Lambers, 1948)	?	12		Blackman and Eastop 2006 [?]
		14		Khuda-Bukhsh and Kar 1990 [Kalyani, West Bengal, India]
Uroleucon (Uromelan) compositae (Theobald, 1915)	P(o)	12		Kurl and Misra 1983 (as *Dactinotus*) [Jodhpur, Rajasthan, India], Khuda-Bukhsh and Kar 1990 [Shillong, Meghalaya, India]
Uroleucon (Uromelan) gobonis (Matsumura, 1917)	P(c), P(o)	12		Blackman and Eastop 1984, Chen and Zhang 1985a [Beijing area, China] (cited after Blackman and Eastop 2015)
		14	XX/X0	Shinji 1927, 1931, 1941a (as *Macrosiphum*), Blackman 1986 [Japan] (based on n(♂) = 7 (Shinji 1931))
Uroleucon (Uromelan) helianthicola (Olive, 1963)	?	12		Olive 1967 (as *Dactynotus*) [USA]
Uroleucon (Uromelan) himachali L.K. Ghosh, 1975	?	14		Kar et al. 1990 [India]
Uroleucon (Uromelan) illini (Hottes & Frison, 1931)	P(c)	12		Blackman and Eastop 2006 [?]
Uroleucon (Uromelan) jaceae (Linnaeus, 1758)	P(c)	12		Blackman 1980 [Great Britain], Kar et al. 1990 [India]
Uroleucon (Uromelan) rurale (Hottes & Frison, 1931)	P(c)	10		Olive 1967 (as *Dactynotus*) [USA]
Uroleucon (Uromelan) taraxaci (Kaltenbach, 1843)	P(c)	12		Sun and Robinson 1966 (as *Dactynotus*), Robinson and Chen 1969a (as *Dactynotus*) [Canada]
Uroleucon (Uromelan) tuataiae Olive, 1963	?	12		Olive 1967 (as *Dactynotus*) [USA]
Uroleucon (Uromelan) verbesinae (Boudreaux, 1949)	?	10		Olive 1967 (as *Dactynotus*) [USA]
*Uroleucon* sp. 1	?	12		Robinson and Chen 1969a (as *Dactynotus*) [Canada] (five different taxonomic forms)
*Uroleucon* sp. 2	?	12		Chen and Zhang 1985a [Beijing area, China] (cited after Blackman and Eastop 2015)
*Utamphorophora crataegi* (Monell, 1879)	P(c)	10		Robinson and Chen 1969a [Canada]
*Utamphorophora humboldti* (Essig, 1941)	P(c), P(o)	20		Robinson and Chen 1969a (as *Myzodes physocarpi* Pepper, 1950) [Canada], Blackman 1980 [Great Britain]
*Vesiculaphis caerulea* Miyazaki, 1980	?	6		Blackman 1986 [Japan]
*Vesiculaphis cephalata* Miyazaki, 1971	P(c)	20		Blackman 1986 [Japan]
*Vesiculaphis theobaldi* Takahashi, 1930	P(c), P(o)	36		Blackman and Eastop 2006 [Great Britain, ?anholocyclic populations]
		38		Blackman and Eastop 2006 [Great Britain, ?anholocyclic populations]
		40		Gut 1976 [Holland]
*Vesiculaphis* sp.	?	24		Gautam and Kumar 2006 [Shimla, Himachal Pradesh, India]
*Wahlgreniella nervata* (Gillette, 1908)	?P(c), P(o)	12		Blackman 1980 [Great Britain]
*Wahlgreniella vaccinii* (Theobald, 1924)	P(c)	12		Blackman and Eastop 2015 [?]
*Xerobion cinae* (Nevsky, 1928)	P(c)	8		Blackman 1980 [Iran]
